# Design, Development, and Evaluation of an Automated Solution for Electronic Information Exchange Between Acute and Long-term Postacute Care Facilities: Design Science Research

**DOI:** 10.2196/43758

**Published:** 2023-02-17

**Authors:** Madhu Gottumukkala

**Affiliations:** 1 College of Business & Information Systems Dakota State University Madison, SD United States

**Keywords:** information exchange, interoperability, care transition, health information technology, health information exchange, open standards, long-term and postacute care, LTPAC, design science research

## Abstract

**Background:**

Information exchange is essential for transitioning high-quality care between care settings. Inadequate or delayed information exchange can result in medication errors, missed test results, considerable delays in care, and even readmissions. Unfortunately, long-term and postacute care facilities often lag behind other health care facilities in adopting health information technologies, increasing difficulty in facilitating care transitions through electronic information exchange. The research gap is most evident when considering the implications of the inability to electronically transfer patients’ health records between these facilities.

**Objective:**

This study aimed to design and evaluate an open standards–based interoperability solution that facilitates seamless bidirectional information exchange between acute care and long-term and postacute care facilities using 2 vendor electronic health record (EHR) systems.

**Methods:**

Using the design science research methodology, we designed an interoperability solution that improves the bidirectional information exchange between acute care and long-term care (LTC) facilities using different EHR systems. Different approaches were applied in the study with a focus on the relevance cycle, including eliciting detailed requirements from stakeholders in the health system who understand the complex data formats, constraints, and workflows associated with transferring patient records between 2 different EHR systems. We performed literature reviews and sought experts in the health care industry from different organizations with a focus on the rigor cycle to identify the components relevant to the interoperability solution. The design cycle focused on iterating between the core activities of implementing and evaluating the proposed artifact. The artifact was evaluated at a health care organization with a combined footprint of acute and postacute care operations using 2 different EHR systems.

**Results:**

The resulting interoperability solution offered integrations with source systems and was proven to facilitate bidirectional information exchange for patients transferring between an acute care facility using an Epic EHR system and an LTC facility using a PointClickCare EHR system. This solution serves as a proof of concept for bidirectional data exchange between Epic and PointClickCare for medications, yet the solution is designed to expand to additional data elements such as allergies, problem lists, and diagnoses.

**Conclusions:**

Historically, the interoperability topic has centered on hospital-to-hospital data exchange, making it more challenging to evaluate the efficacy of data exchange between other care settings. In acute and LTC settings, there are differences in patients’ needs and delivery of care workflows that are distinctly unique. In addition, the health care system’s components that offer long-term and acute care in the United States have evolved independently and separately. This study demonstrates that the interoperability solution improves the information exchange between acute and LTC facilities by simplifying data transfer, eliminating manual processes, and reducing data discrepancies using a design science research methodology.

## Introduction

### Research Problem

According to the Office of the National Coordinator for Health Information Technology, the most frequently reported barrier to the electronic exchange of patient health information was difficulty in exchanging data across different electronic health record (EHR) vendor platforms [1]. More than 50% of the hospitals indicated that their exchange partners could not receive data because their EHR system lacked the capability to electronically transfer data or because their exchange partners lacked a system to electronically receive the data [1]. Nearly 5 million patients transition to and from hospitals and skilled nursing facilities (SNFs) each year [2]. From an SNF perspective, which traditionally provides short-term, subacute care for persons recuperating from hospitalization or an acute condition [3], transition problems stem from inadequate hospital discharge handoff communication, such as missing information, medication order problems, or misleading behavioral status information [2]. SNFs reported substantial shortcomings in the completeness, timeliness, and usability of the information provided by hospitals to support patient transitions [4]. The deficiencies in all 3 areas are linked to a poor transition experience, indicating that hospitals have not put enough effort into identifying and meeting the information needs of SNFs to facilitate the transfer of care from the acute setting [4]. Clinicians and staff at postacute care (PAC) facilities receiving hospital transfers reported substantial deficits in the completeness and timeliness of the information received upon hospital discharge [5]. These deficits suggest an opportunity to improve the timeliness of the information provided to PAC facilities during this critical time [5]. A study [4] published in the *American Medical Association Open Network Journal* highlighted the data exchange challenges associated with acute care and long-term and postacute care (LTPAC) facilities fraught with silos of data and different maturity levels [6]. The LTPAC providers have grown accustomed to receiving paper documents and faxes or calling the sending provider to obtain patient-specific information [6]. Unfortunately, this information may be insufficient or contain so much superfluous data that the receiving physician must spend additional time locating and examining the information required for treatment [6]. Most nursing facilities use an EHR, but many respondents cannot send electronic information to other organizations or receive, integrate, or search for electronic information from other organizations [7]. The most commonly identified barrier to sharing clinical information among nursing facilities with an EHR was a reported absence of interoperability [7].

### Research Problem as Addressed in Extant Literature

According to the Office of the National Coordinator for Health Information Technology, hospitals adopt different methods to exchange patient information [1]. These include methods used without intermediaries—such as mail or fax, eFax using EHR, provider portals with view-only access to an EHR system, interface connection between EHR systems, and direct access to EHRs via remote or terminal access [1]—and methods used with intermediaries—such as third-party health information service provider or health information exchange (HIE) enabling secure messaging, EHR vendor–based network enabling exchange with the vendor’s other users, and national networks that enable exchange across different EHR vendors [1].

Although some LTPAC organizations leverage EHR systems, the systems are often very specific to the line of service provided [8]. Hospitals, SNFs, home health services, and assisted living health information systems (IS) have minimal interfaces that integrate the respective systems, essentially making each system its own silo [8]. Therefore, the continuity of care also suffers because transferring pertinent patient information is often done manually through phone calls, faxes, email, and even paper documents [8]. The US health care system, LTPAC in particular, still relies upon a fax machine to communicate summaries of care when a patient is discharged, which is acknowledged as not an effective, efficient, and sustainable communication plan [9]. The study found that when lengthy summaries of care are delivered or faxed, it is very likely for some pages to go missing or be out of order and for clinicians and staff to be unable to find pertinent medical information efficiently [9]. The prevalence of inadequate information in the summaries of care that are delivered when patients are transitioning from a hospital has underscored the problems that clinicians and staff face when trying to communicate and clarify questions on medications and the care plan [9]. HIE is a small subset of health information technology (HIT) [10]. It has emerged as an information-based approach with a potential to expand the amount of information exchanged at patient handoff [11]. Very little is known about the adoption of HIE among hospitals and long-term care (LTC) facilities at the national level [10]. The most common barrier to HIE adoption is a lack of interoperability and missing, incomplete, or inaccurate data associated with HIE [10]. The other barriers cited were a lack of data standards and privacy and security concerns [10]. A study [12] assessed 3 types of information exchange, namely secure messaging, provider portals, and the use of an HIE, and concluded that only some methods consistently provided high levels of usable, integrated health information. Instead, adopting additional methods was associated with a greater likelihood that hospitals could routinely access and integrate patient information electronically [12]. Although progress has been made in HIE, the complexity of engaging in widespread exchange has also increased, leading to a patchwork of connectivity that requires providers to seek multiple solutions to engage in HIE [12]. According to another study [13], HIE is adversely associated with mortality. The lack of interoperability standards is cited as a barrier to seamless HIE [13]. Mismatch of patient records during data transfers between health systems and lack of cooperation and consensus among providers are other reasons cited as barriers to information exchange using HIE [13]. Another study [5] proposed the use of internet-based portal access to improve information exchange. However, they suspected the need for an additional multifaceted intervention to address the issues reported that are not fully addressed by providing staff and clinicians with read-only access to EHR [5]. The authors recommended that an optimal intervention for addressing the completeness and timeliness issues is the implementation of bidirectional information exchange and communication between the hospital and PAC setting that is integrated into the clinical workflow for users in both the hospital and PAC facility [5].

### Health Intervention Technologies and Research Gap

Difficulty with cross-vendor exchange, difficulty integrating information into EHR systems, timeliness of data, and usability issues are among the top reasons cited as barriers to the electronic exchange of information between hospitals [1]. Furthermore, the lack of a software designed for unique LTC needs is a common barrier to technology adoption [14]. In addition, PAC providers want the EHR platforms to be tailored to their area but continue to face care coordination challenges [15]. A key outcome in health care stakeholders’ adoption of health information and technology is the capability to exchange data seamlessly among different health care IS [6]. Interoperability would enable information transfer during transitions and lay the groundwork for numerous opportunities for HIT to support care transition [16]. However, the lack of interoperability between vendor EHR systems is a barrier to developing new HIT tools for effective information exchange [16]. Research suggests that there is a need for innovative HIT tools to facilitate information exchange between acute care and LTC facilities. However, most organizations use case managers and nurses for care transitions [16], and only a few HIT-supported intervention studies were found in the biomedical literature listed subsequently. Although HIT is used for information exchange, there are considerable gaps owing to the need for interoperability.

Gray et al [17] developed a digital bridge solution, a novel approach to improve interprofessional communication in hospitals supporting patient-centered health care transitions for older adults with complex care needs. However, one of the limitations of this study was that the digital bridge could only partially integrate with the hospital-based EHR owing to a lack of interoperability [17].

Austin et al [18] implemented a complex health system intervention to improve the care transition for patients with complex medical conditions from hospitals to PAC facilities. The design components included a tailored risk calculator, a comprehensive screening tool, a multidisciplinary team with the capacity to address complex barriers to safe transitions, and enhanced discharge workflows. This approach had limitations and needed modifications to address broader populations [18].

Saeed [19,20] envisioned a custom design of HIT and established a web portal that connected emergency departments and remote psychiatric providers, allowing them to share electronic health information and delivering the necessary data for administrators to operate the program. Without a viable HIE, this solution satisfied most use cases using secure messaging to communicate clinical information attached to the electronic health information [19,20].

Yeaman et al [14] studied the role of HIT and information exchange across care settings and its impact on readmission rates by implementing a pilot project facilitating electronic information exchange between LTC and acute care facilities using HIT. This study implemented a lightweight electronic clinical documentation tool to be mounted outside the resident’s room and used secure messaging to communicate clinical information and exchange clinical documents. However, this approach had limited applicability and allowed only basic information exchange. In addition, it is uncertain whether the same approach would work for other care settings and could be generalized to the broader population [14].

### Problem Opportunity and Research Purpose

HIT helps health care practitioners gather, store, and communicate essential health information reliably and securely across care settings to promote coordinated and patient-centered care, especially during shifts from one care setting to the other. When a patient from an acute care EHR system is transferred to an LTC EHR system and vice versa, the patient’s chart will follow the patient. However, most of the salient information following the patient during the transfer, including demographics, allergies, medications, and discharge orders, is in paper form. The problem discussed in this study is the lack of seamless information exchange between acute care and LTPAC facilities that use different EHR systems. This problem results in discharge and admit information being manually documented twice in each system, which encumbers transferring patients between these facilities, resulting in delayed admission and medical errors. Overall, it is essential to ensure that information follows the patient across care settings in an interoperable way so that data can be moved from one system to another without putting too much strain on resources. Both acute care and LTPAC providers are more likely to meet the triple aim if they use an efficient and streamlined method to access data [6].

### Research Question

The research question driving this design science research (DSR) study is as follows: how can a new interoperability solution facilitate seamless electronic information exchange between acute and LTC facilities using different EHR systems?

Following the DSR framework, we designed a new interoperability and information exchange solution by adopting open standards consisting of multiple components that can be deployed to meet complex health care integration and workflow needs. This study demonstrates that the proposed solution enhances the information exchange process between acute and LTC facilities by simplifying data transfer, eliminating manual processes, and reducing data discrepancies.

## Methods

### Overview

**Figure 1:**
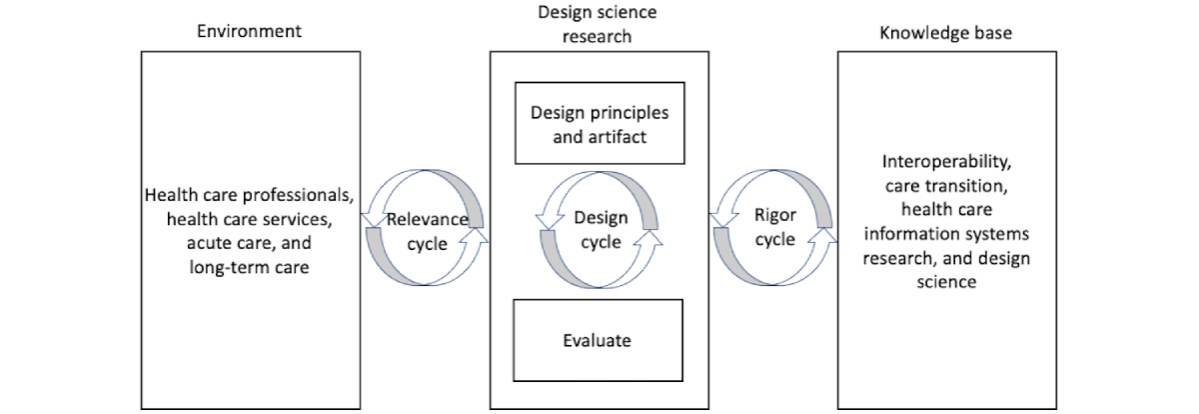
A design science research approach for automated electronic information exchange.

The research design and methodology adopted for this study is DSR, which focuses on creating and evaluating IT artifacts to solve identified organizational problems [21]. Design science is, by nature, a problem-solving process. DSR necessitates the development of an innovative, useful artifact for a specified domain [21]. DSR strongly emphasizes application domain relevance while focusing on the IT artifact [22]. A novel and useful artifact contributes to design knowledge [23]. IT artifacts are broadly defined as constructs (vocabulary and symbols), models (abstractions and representations), methods (algorithms and practices), and instantiations (implemented and prototype systems) [21]. Two clearly identifiable artifacts were produced in this study. The first is the architecture design showcasing the method as a set of steps used to facilitate the information exchange process. The second research artifact in this study is an instantiation of the proposed architecture design, which involved its deployment in a health care setting to facilitate automated electronic information exchange between acute and LTC EHR systems. We adopted the guidelines provided by Hevner et al [22] for implementing the DSR and applied them to our study, as shown in Figure 1. The environment defines the problem space where the phenomena of interest reside. Research relevance is ensured by framing research activities to address the business requirements. Design science addresses research by creating and evaluating artifacts that are intended to meet the identified business needs. Utility is the goal of DSR. The knowledge base offers general guidelines and pathways to IS research. Foundations and methodologies constitute the knowledge base. Prior IS research and results from reference disciplines help develop and build foundational theories, frameworks, instruments, constructs, models, methods, and instantiations. Methodologies guide justification or evaluation. Applying established foundations and methods fosters rigor [21,22].

Different approaches were applied in the study with a focus on the relevance cycle, including eliciting detailed requirements from stakeholders in the health system who understand the complex data formats, constraints, and workflows associated with transferring patient records between 2 different EHR systems. The design and the architecture were continuously revised, working with other health care personnel (administrators) and IT professionals, including system administrators and expert vendor associates. Literature reviews were conducted and experts in the health care industry from different organizations, including NextGen, PointClickCare (PCC), and Epic, were sought with a focus on the rigor cycle to identify the components relevant to the interoperability solution. The design cycle focused on iterating between the core activities of implementing and evaluating the proposed artifacts.

### Requirements Elicitation

**Figure 2:**
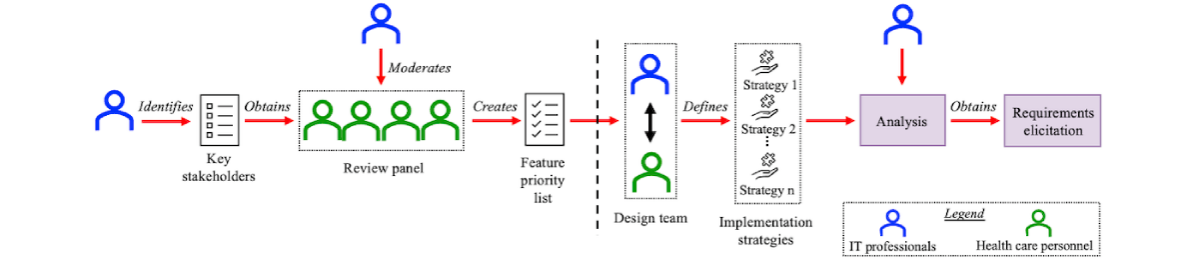
Requirements elicitation framework.

HITs have great potential to change how health care is delivered and enhance patient outcomes. However, the implementation of any health intervention technology requires improvements in data quality and reliability issues, patient safety, usability, and privacy issues [24]. Close and early engagements across stakeholders will be essential to ensure that health technologies improve outcomes, contribute value to health care systems, cut costs, and improve care quality [24]. In addition, health systems are composed of multiple components. Therefore, developing and deploying health intervention technologies may involve the interaction of one or more components of a health system, making the requirement elicitation process an important and complex activity. Requirement elicitation is a collaborative process between users and stakeholders that reveals the software’s required features, functions, and properties [25]. We used the requirements elicitation framework shown in Figure 2 for this solution inspired by dissemination and implementation research [26] and the dissemination approach proposed by Steensma et al [27] and Márquez et al [28] to strategically elicit requirements from the stakeholders following the guidelines [29] that support the interoperability solution development.

The first step in this process is the stakeholder and feature priority identification. We adopted the guidelines proposed by Anwar et al [30] in arriving at the stakeholder selection process. The stakeholder selection process involved understanding and documenting the context in which the interoperability solution will be developed. Once the context was outlined, we identified the stakeholders who engage with the health intervention technology. The framework used a review panel composed of already identified stakeholders, who identified and prioritized the interventions that the interoperability solution should address. This analysis helped HIT professionals to support technology decisions. The IT professional in the role of a moderator recorded the rationale and priorities to arrive at a feature priority list for the solution. The design team consisted of a multifunctional group of IT professionals and health care personnel who converted those priorities into possible implementation strategies that enabled the interoperability solution to address the feature priorities. Once the implementation strategy was finalized for the targeted and prioritized intervention, the IT professionals analyzed the prioritized features and implementation strategy to elicit requirements from the relevant stakeholders by following the guidelines proposed by Tiwari et al [29]. On the basis of the requirements, feature priority list, and recommendations from the design team and the review panel, we finalized an interoperability solution design as an implementation strategy to enable a seamless exchange of data between the acute care EHR system and the PAC EHR system.

### Problem Exploration and Context

Most acute care hospital systems today use Epic as their EHR platform, and many LTC facilities use PCC as their EHR platform [31]. Epic and PCC use different interoperability standards, which prevents data from being electronically exchanged between the 2 systems. The current workflow between a typical acute care (Epic) facility and LTC (PCC) facility during care transition involves a discharge summary in a paper document sent along with the patient transferring from one facility to the other. Salient information from the paper document, such as demographics, allergies, and medications, are re-entered manually into the receiving facility’s EHR system. This manual process results in delayed admission and human errors.

High-level technical requirements have emerged from interviews with the subject matter experts that will allow patient information to flow automatically and electronically to improve the bidirectional information flow between acute care and LTC facilities operating on Epic and PCC EHR systems, respectively. The interoperability system should be able to do the following:

1. Integrate with the acute care Epic EHR system and the PAC PCC EHR system
2. Receive patient information including the discharge summary and other critical patient information such as medications, allergies, problem lists, and diagnoses from Epic via Health Level Seven International (HL7) and Fast Healthcare Interoperability Resources (FHIR) application programming interface (API) standards
3. Send discharge information received from PCC to Epic via Consolidated Clinical Document Architecture or FHIR APIs
4. Send patient information received from Epic to the PCC system via APIs
5. Receive patient information from PCC via APIs

### Solution Design and Implementation

#### High-Level Solution Overview

##### Outline

In this section, we present an overview of a pilot interoperability solution that will facilitate bidirectional information exchange for patients transferring between acute and LTC facilities using different EHR systems. This open standards–based solution will consume HL7 version 2 admission, discharge, transfer (ADT) information; HL7 FHIR information; and PCC’s APIs and will be able to populate HL7 version 3 Continuity of Care Documents (CCDs) and PCC’s APIs. The solution is hosted in the acute care facility network facilitating the information exchange between the 2 EHR systems. This pilot study will serve as a proof of concept for bidirectional data exchange between Epic and PCC for medications. However, it is easily expandable to additional data elements such as allergies, problem lists, and diagnoses.

##### Transfer From Acute Care to LTC Facilities

**Figure 3:**
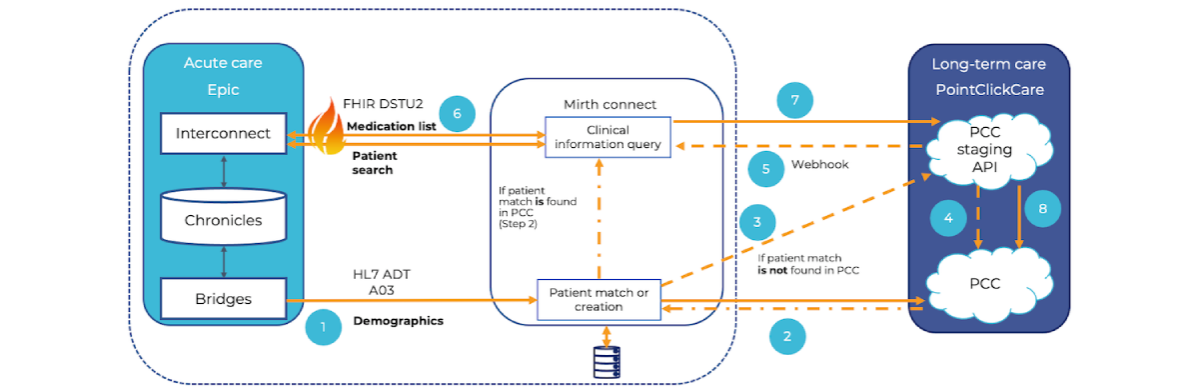
Information flow for patients transferred from acute care to long-term care facilities. ADT: admission, discharge, transfer; API: application programming interface; DSTU2: Second Draft Standard for Trial Use; FHIR: Fast Health Interoperability Resources; HL7: Health Level Seven International; PCC: PointClickCare.

Figure 3 illustrates the high-level design that facilitates information exchange from acute care facility to LTC facility. The enumerated flow of information is as follows:

1. When a patient is discharged in Epic, an ADT A03 message is generated on the outgoing ADT interface. The ADT messages will be forwarded to Mirth Connect based on PV1-37, the discharge location. Mirth Connect is configured to listen for these ADT messages from acute care Epic and to send an acknowledgment message back to acute care upon receiving a message.
2. Mirth Connect will parse the ADT A03 message and create a POST PatientMatch API call to PCC using the demographic information. PCC will return the patient ID if a patient match is found, and the process will skip to step 6.
3. If a matching patient is not found, a POST pending patient message will be sent to PCC. This will create a pending patient in PCC that can be searched or merged with an existing patient.
4. The pending patient created by step 3 will be confirmed or merged by a user in PCC. Before the patient is admitted, a patient record will be created in the Customer Relationship Management system for administrative and referral purposes. Once the patient has been created in the Customer Relationship Management and the details have been confirmed, the patient will be moved from the *Pended* status to *Received* status. (This may require the patient arriving at the actual facility.)
5. The patient being merged or confirmed will trigger a webhook from PCC to Mirth Connect. This webhook will contain the new patient ID as well as the resource ID, which allows Mirth Connect to link the patient with the original pending patient API call.
6. Once the PCC patient ID is known (after a patient match or a pending patient webhook), Mirth Connect will create an FHIR query to find the patient’s FHIR ID in Epic. With the patient’s FHIR ID, an FHIR MedicationStatement query is used to gather the patient’s medications.
7. With the medications gathered from Epic, they can now be pushed to PCC. To submit historical medication into PCC, a “Care Period” must be created first. The admission and discharge dates will be used to construct the care period. The POST CarePeriod API will be called to generate a care period, and then the POST HisoricalMedications API will be used to push the medications gathered from Epic to PCC. While the MedicationStatement FHIR query returns multiple medications, the PCC API can only accept one medication at a time. Hence, PCC Medication API (POST HistoricalMedications) will be called as many times as the number of medications returned by the Epic FHIR API call.
8. The end user will then reconcile the medications and activate them to add them to the patient’s chart in PCC.

##### Transfer From LTC to Acute Care Facilities

**Figure 4:**
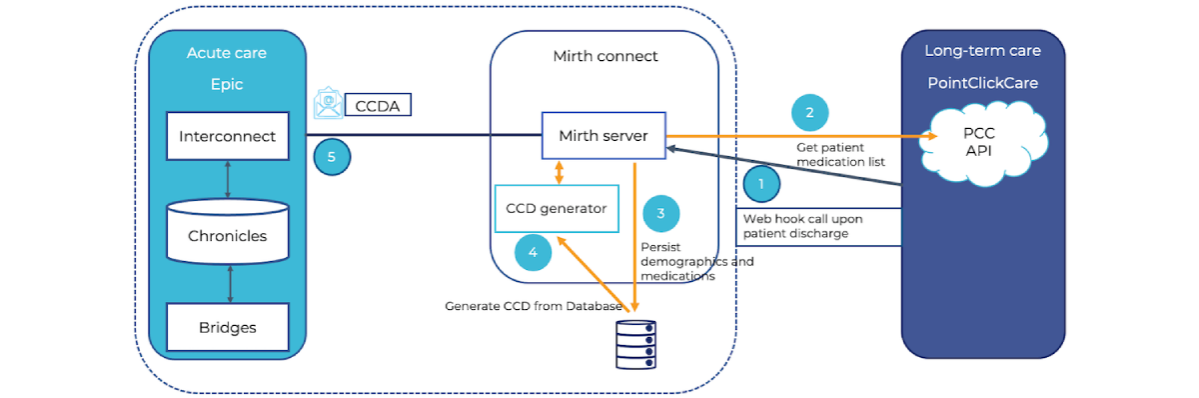
Information flow for patients transferred from long-term care to acute care facilities. API: application programming interface; CCD: Continuity of Care Document; CCDA: Consolidated Clinical Document Architecture; PCC: PointClickCare.

Figure 4 illustrates the high-level design that facilitates information exchange from long-term to acute care facilities. The enumerated flow of information is as follows:

1. When a patient is discharged, a webhook message will be sent to Mirth Connect. This webhook serves as the trigger event for Mirth Connect to begin information exchange. Only patients who have a relationship with acute care will generate a webhook event when discharged. In other words, only patients who have either been admitted from or are being discharged to an acute care hospital will generate a webhook message.
2. Mirth Connect will use the GET Patient and GET PatientMedication calls to gather demographic and medication data for the discharged patient from PCC.
3. The demographics and medication list are stored in SQL server.
4. Mirth Connect calls CCD generator (stand-alone app), which, in turn, generates a CCD from the information stored in the database.
5. The Generated CCD is pushed to Epic via the eXternal Data Representation protocol. Using the Care Everywhere app, providers can then reconcile the medication information received from PCC into Epic.

#### Overall Solution Architecture

##### Outline

**Figure 5:**
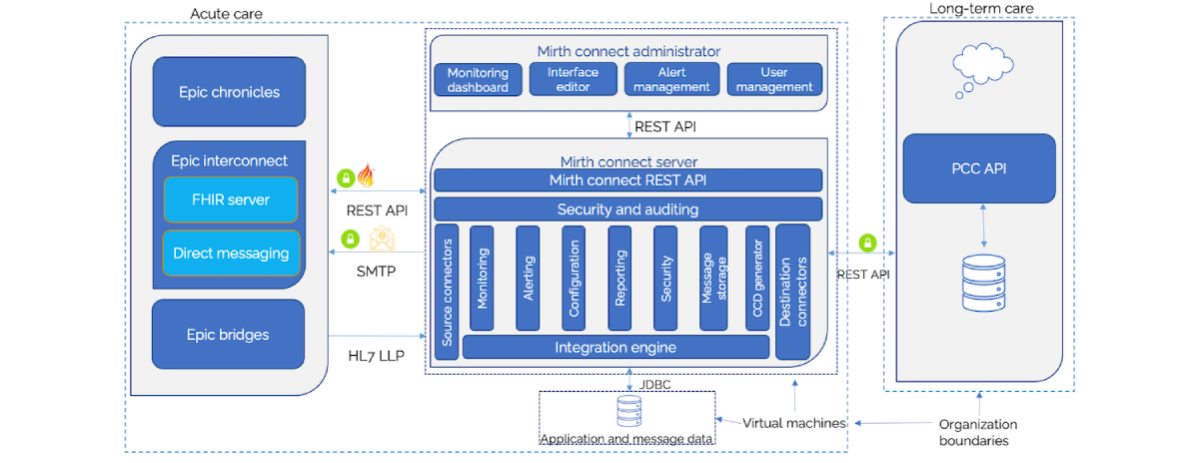
Solution architecture—physical and deployment. API: application programming interface; FHIR: Fast Health Interoperability Resources; HL7: Health Level Seven International; JDBC: Java Database Connectivity; LLP: Lower Layer Protocol; PCC: PointClickCare; REST: REpresentational State Transfer; SMTP: Simple Mail Transfer Protocol.

The 3 systems involved in this design are Epic EHR Suite, Virtual Machine (VM) in the demilitarized zone environment for hosting Mirth Connect Integration tool and SQL server, and PCC-hosted solution, as shown in Figure 5.

Different communication and security protocols are used in this architecture:

1. HTTPS/basic authentication for communication between Mirth Connect and Epic FHIR end points
2. Transmission Control Protocol/Internet Protocol and HL7 Lower Layer Protocol for communication between Epic Bridges and Mirth Connect
3. HTTPS, Secure Sockets Layer (SSL), and Open Authorization 2.0 2-legged authentication for communication between Mirth Connect and PCC
4. HTTPS/SSL connection for communication between Mirth Connect and PCC’s APIs

The Mirth Connect Integration tool offers many features for various requirements that are common to HIT. The following features were used for the scope of this implementation:

1. Interface editor—to create and manage the channels that contain the source and destination configurations and the transformation logic.
2. Monitoring dashboard—to monitor the different channels’ activities; the dashboard provides details regarding the number of messages received, transformed, failed, sent, etc
3. Message storage—to store the incoming messages; we can store raw messages and parsed messages to a persistent layer, which for this implementation is done using SQL server

**Figure 6:**
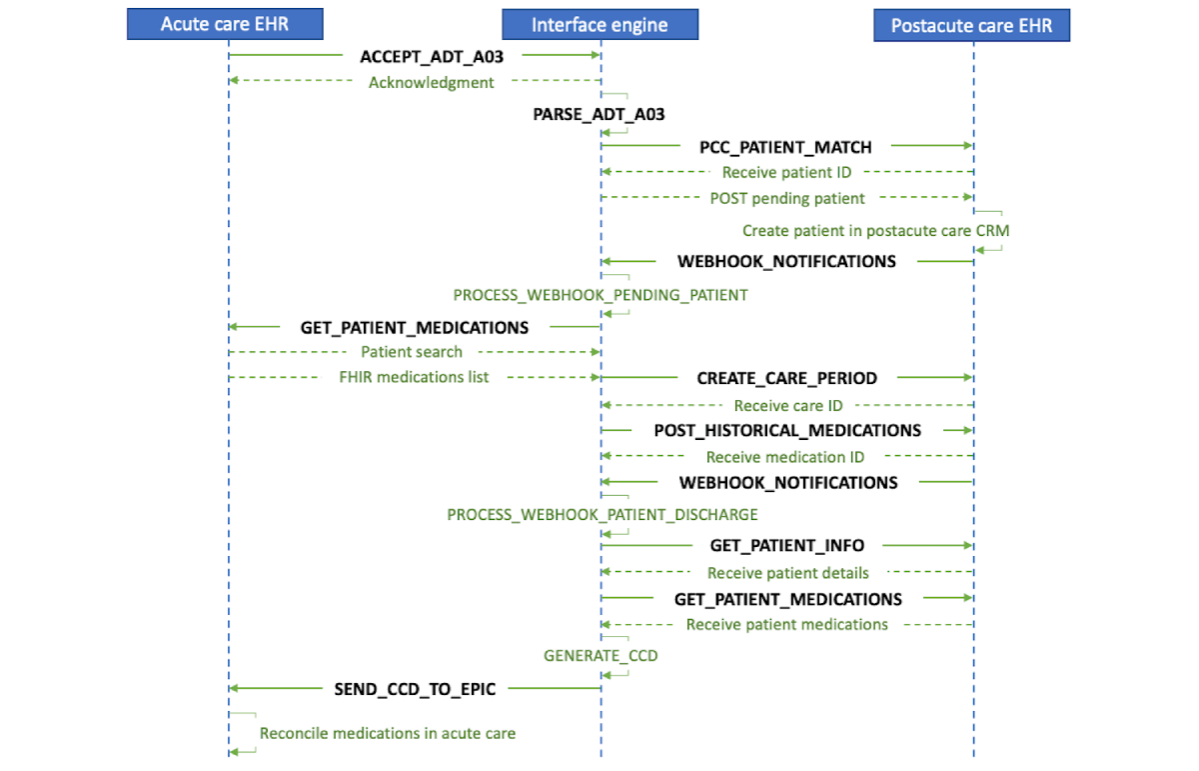
Unified Modeling Language action diagram of interactions between systems. ADT: admission, discharge, transfer; CCD: Continuity of Care Document; CRM: Customer Relationship Management; EHR: electronic health record; FHIR: Fast Health Interoperability Resources; PCC: PointClickCare.

A Unified Modeling Language–based action diagram of the interaction between components is depicted in Figure 6, followed by a brief narrative of the interaction. The sequence diagram shows the flow of data for the 2 scenarios of information exchange—from acute care facility to PAC facility and from PAC facility to acute care facility.

##### Patient Transfer From Acute Care to PAC Facilities

1. ACCEPT_ADT_A03 and PARSE_ADT_A03: these 2 channels are responsible for receiving and parsing the ADT03 message that is sent from Epic Bridges when a patient is discharged in the hospital system. Messages are sent via Transmission Control Protocol/Internet Protocol and HL7 Lower Layer Protocol to the interface engine from the Epic Bridges interface.
2. PCC_PATIENT_MATCH: once the message is parsed, this channel will send patient details to PCC API using the Open Authorization 2.0 authentication protocol to determine whether a patient match is found. If a patient match is found, then PCC API responds with a PCC patient ID, which will be used in subsequent calls.
3. PCC_PATIENT_MATCH: if the patient is not found in PCC, then this channel will submit a new patient record request.
4. WEBHOOK_NOTIFICATIONS: webhook notifications for the pending patient are received when the patient has been created in the PCC system and a patient ID is generated.
5. GET_PATIENT_MEDICATIONS: this channel is responsible for obtaining patient medication information by connecting to the acute care facility’s Epic Interconnect FHIR end point using basic authentication, that is, sending client_ID and user credentials with every request.
6. CREATE_CARE_PERIODS, POST_HISTORICAL_MEDICATIONS: in the PCC system, the medications are associated with a care period. The care period is a notional period that begins when a patient is admitted to an acute care facility and ends when the patient is discharged. This information is gathered from the ADT A03 message and is used to create a care period in PCC using the HTTP POST API.
7. Reconciliation of patient medications to the patient record in PCC is done by the user.

##### Patient Transfer From PAC to Acute Care Facilities

1. WEBHOOK_NOTIFICATIONS: this channel receives a webhook notification from PCC when the patient is discharged from a long-term facility and admitted to an acute care facility.
2. GET_PATIENT_INFO: for the patient in step 1, this channel calls the PCC API to obtain patient demographic details and stores them in the database.
3. GET_PATIENT_MEDICATIONS: this channel collects the patient medications from PCC and stores this information in the database.
4. The Java component on the interface engine VM is scheduled to look for new patients who are discharged and generate a CCD from the saved demographics and medications for the transferred patient.
5. SEND_CCD_TO_EPIC: the CCD is transferred to Epic’s Interconnect system’s eXternal Data Representation end point using the SSL authentication with certificates established between the Interconnect system and the interface engine VM.

More details on CCD generation and the associated class diagram are provided in Multimedia Appendix 1.

#### Data Model

The tables that are used to satisfy the goals of this pilot have been grouped by subject areas. Each subject area contains related tables. The subject areas that have been created for this solution include the following:

1. Common message table: these tables are used for housekeeping of the raw messages and state transition of messages.
2. HL7 interface–related tables: these tables are used for storing the information parsed from HL7 ADT A03 message.
3. EPIC interface–related tables: these tables are used to store the information required for to or from communication with Epic.
4. PCC interface–related tables: these tables are used to store the information required for to or from communication with PCC.

The data model for each subject area and the associated tables are shown in Multimedia Appendix 2.

### Evaluation

The artifact was evaluated at a health care organization with a combined footprint of acute care and PAC operations using 2 different EHR systems. To further address the relevance and utility of the artifacts produced in this research, a Framework for Evaluation in Design Science was used to determine their functional purpose. The components identified for the interoperability solution were reviewed by experts in the health care industry from different organizations, including health care personnel at acute care, LTC, NextGen, PCC, and Epic, relevant to the information exchange, patient data formats, standards, and associate protocols in the context of acute and LTC facilities. A formative evaluation was conducted through an expert architecture design review to mitigate technical risks. The design was evaluated against the elicited requirements to ensure that the underlying system design was robust enough to support the features of the automated information exchange process. The system and subject matter experts reviewed the solution architecture, technical architecture, data models, and underlying database structure. The essential elements for reviews, such as the system’s functional and nonfunctional requirements, design considerations, performance, and compliance that are needed for this design, were captured. We ran a system test on our solution to ensure a reliable formative evaluation of the product in a nonproduction scenario. A team of skilled software testers with health care domain expertise conducted system testing. In addition, the team created system test scenarios and test cases based on the solution’s requirements and design. Throughout the implementation phase, we adopted an agile approach to system testing in which issues were detected and resolved iteratively, and stakeholders were informed through regular feature demonstrations. The connections configured to satisfy this interoperability solution’s business and technical needs were validated (Multimedia Appendix 3) according to the interaction and sequencing of the Mirth channels, as described in the Unified Modeling Language action diagram in Figure 6. As the solution was deployed in a live environment, all the features were thoroughly tested. All Epic and PCC EHR user interface screens, actions, integrations, and end-to-end functionality, including positive and negative scenarios, were tested to ensure maximum test coverage.

We conducted a formative field-based usability testing involving the health care domain experts who were part of the design and implementation team throughout the development process [32]. We adopted “Think Aloud” and “Near Live” usability testing techniques to generate insights into the workflow, navigation, content, and ease of understanding of the solution [33]. We provided “Think Aloud” testers with written user stories and acceptance criteria to gather feedback while interacting with the new Epic and PCC workflow screens. “Near Live” testers interacted with a test patient while using the solution. Both the techniques generated unique insights into the proposed solution. However, whereas the “Think Aloud” testing provided insights into improving the ease of use and understanding of the new solution features for the end users, the “Near Live” testing was instrumental in eliciting barriers to and facilitators of the proposed solution [33] from the stakeholders and end users, which led to workflow changes and adoption. The systems’ detailed screens, messaging channels’ configurations, messaging standards and protocols, message sequencing, and the corresponding storage of the final documents were tested and validated step by step to ensure seamless information exchange according to the design and the specifications required for transferring patients between the facilities. The detailed steps are presented in Multimedia Appendix 4.

The solutions’ effectiveness from a human perspective was also assessed by performing usability testing [34] with health care administrators at acute and LTC facilities under the guidance of EHR vendor system experts to address user-centric concerns. The final walkthrough of the user interface for patient transfer with automated information exchange process steps and the associated screenshots of the systems are shown in Multimedia Appendix 5. Furthermore, end-to-end system testing was performed on the systems to evaluate the resilience of the artifact in a summative manner. The strategy involved an agile method of system testing to address any stakeholder concerns based on the requirements and design of the artifact.

## Results

### Pilot Rollout

The proposed interoperability solution successfully replaced the manual process by allowing health care professionals to electronically transfer patients’ information upon their discharge from a health care setting. A roundabout mock-up use case was executed on the design, which involved the discharge of a test patient from an acute care facility using the Epic EHR system and admission of the patient to the PAC facility using the PCC EHR system. To complete the loop, the test patient was discharged from the PAC facility and admitted to the acute care facility. During the pilot rollout phase, patients were enrolled because of their presence in the systems where the program was used. They were naturally pulled into the process when a transfer need arose. We used the existing standards and regular procedures for involving the legal and privacy teams to ensure that patient health information was shared as needed. The end user journey of the aforementioned scenarios is documented (Multimedia Appendix 5). Overall, there was positive feedback on the design and implementation from all the stakeholders involved in the patient discharge process who typically engage with the different components of the systems. The primary feedback came from group discussions during the pilot phase. The people involved were IT application owners, clinical informatics staff, end users, and leadership in the LTC hospital system. Once the use case of medication exchange was successfully demonstrated, we prioritized to finalize the workflow for other scenarios, including allergies, problem lists, and diagnoses. The pilot program started with 1 LTC facility in Luverne, Minnesota. Since then, it was expanded to be deployed in 9 more facilities in the Midwest region, namely 3 in Sioux Falls, 4 in Bismarck, 1 in Blackduck, and 1 in Canton.

### Opportunities

The study results were positive and helped save time and design more accurate data transfer. However, during the transfer process, some types of data can be directly added to the patient chart, whereas others require a manual review by a clinician before being added to the patient chart. Therefore, an area of improvement would be to continue improving the amount of data that can be automatically added to the chart compared with the amount of data that must be manually reviewed. The metrics were straightforward in the sense that any patient information or data transferred between the 2 vendor EHR systems must be 100% accurate. We did not calculate the percentage of data that were added directly to the patient chart or the percentage of data that had to be manually reviewed. Data transfer was successful for the data types that could be mapped between systems. For example, allergy data have a simple structure with limited variables. The simple structure allows for the complete mapping of expected data elements and will enable allergies to be automatically added to the patient chart. However, medications are a more complicated data set, given the fact that many medications are available, the variety of units and frequencies that can describe a medication dose, and the complications that could be associated with a medication. In the case of complex data sets such as medications, it is common to place them in a reconciliation area for a clinical user to review and either manually add them to the patient chart or discard them if the information is unclear. The pilot allowed us to continue to expand the number of facilities using this information exchange process and benefit from the seamless automated and accurate electronic data transfer.

## Discussion

### Principal Findings

In the United States, the components of the health care system that offer acute care and LTC have evolved independently and separately [31]. Until recently, the information exchanges between these 2 health care system components were manual and labor intensive [31]. Shareable EHRs and interoperability are crucial for improving patient health outcomes and saving health care system expenditures [31]. The proposed interoperability solution will benefit the health care community during the transition of care with coordination between acute and LTPAC providers. Although IS research in health care interoperability has surged recently, only a few studies have addressed the challenges of information exchange between acute and LTC facilities. The literature review provided insights into the existing research on the information exchange and HIT challenges at LTPAC facilities, which set the path to establishing the components required for the initial artifact design. The components identified were reviewed by the experts in the health care industry from different organizations relevant to the information exchange, patient data formats, standards, and associated protocols in the context of acute care and LTC facilities.

This research’s artifacts will contribute theoretically and practically through their exploration, evaluation, and validation within the acute care and LTC context of information exchange. From a theoretical perspective, this study divided the design knowledge into problem and solution spaces [35]. The solution space addressed technical viability and product usability, and the problem space validation addressed the relevance of the research study. From a practice perspective, the proposed interoperability solution demonstrates a novel approach facilitating seamless information exchange between acute care and LTPAC facilities. In addition, since the study involved a health care artifact, care was taken to comply with the Health Insurance Portability and Accountability Act of 1996 laws to safeguard privacy, and the study adhered to Health Insurance Portability and Accountability Act laws by using test data provided by health care facility administrators. The scope of this study could be expanded if the researchers investigate how the automated electronic information exchange solution between these facilities can incorporate decision support into the information exchange process. This would imply that a patient discharged from an acute care hospital with specific problems and transferred to an LTPAC facility could automatically trigger the decision support suggestion that certain medications be ordered before the patient’s arrival. The study acknowledges that any IT artifact embodies both social and technical elements of system development and that the interactions between business and technical activity systems determine the success of that artifact [34]. Although the study focused on the seamless transfer of patient information between acute care and LTC facilities, it may contribute to health care by building on this solution to develop an innovative digital health platform with the aim of collaboration across the health care ecosystem. This study demonstrates the viability of such an application. It also contributes to how DSR can be used as a strategy to find useful solutions to problems by refining an artifact to fit the needs of the context in which it is used [36].

### Limitations

The pilot interoperability solution was designed, implemented, and deployed at a health care organization with a combined footprint of acute care and LTC operations. The artifact was evaluated and tested for seamless information exchange between Epic and PCC EHR systems. There might be variations in how other health care organizations have their EHR systems configured and workflows defined, which may require some modifications and tuning of the design accordingly. The pilot study has only verified the medication exchange between these 2 facilities. However, the design supports and can be easily extended to allergies, problem lists, diagnoses, and other data elements.

### Conclusions

Most acute care hospital systems today use Epic as their EHR platform, and many LTC facilities use PCC as their EHR platform. Both platforms are proprietary, use different interoperability standards, and serve a similar purpose; however, they do not communicate, preventing data from being electronically exchanged between the 2 systems. Information exchange is essential for the transition of high-quality care between care settings. Unfortunately, LTPAC organizations such as nursing homes and SNFs are considerably behind other health care settings in EHR adoption and health data exchange. Approximately 86% of LTC administrators have reported that their facilities are not exchanging health data electronically with referring hospitals, physicians, and home health providers. The research showed that although LTPAC providers are receiving more attention, this has yet to be converted into a robust, bidirectional information exchange between LTPAC providers and major trading partners such as hospitals and medical organizations. Secure messaging, provider portals, and HIEs are the most commonly used information exchange techniques in these settings, but only some methods consistently provide high levels of usable, integrated health information. The pilot interoperability solution adopted open standards consisting of multiple components that facilitate seamless information exchange between EHR systems and can be deployed to meet complex health care integration and workflow needs. This study provides valuable insights into how the proposed design might be enhanced to deliver optimal patient care by providing real-time access to patient details. The findings of this study will be published in journal articles and presented at health care conferences. In addition, this research also establishes a foundation based on which HIT practitioners and future researchers can build a systematic approach to improve an essential health care operational process.

## Appendix

Multimedia Appendix 1 Continuity of Care Document generation.

Multimedia Appendix 2 Data models and tables.

Multimedia Appendix 3 Interface channels and interactions.

Multimedia Appendix 4 Design review validation.

Multimedia Appendix 5 End user journey.

## Abbreviations

ADT: admission, discharge, transfer
API: application programming interface
CCD: Continuity of Care Document
DSR: design science research
EHR: electronic health record
FHIR: Fast Health Interoperability Resources
HIE: health information exchange
HIT: health information technology
HL7: Health Level Seven International
IS: information systems
LTC: long-term care
LTPAC: long-term and postacute care
PAC: postacute care
PCC: PointClickCare
SNF: skilled nursing facility
SSL: Secure Sockets Layer
VM: virtual machine

## Data Availability

All data generated or analyzed during this study are included in this paper and its supporting Multimedia Appendix files.
