# Supplementary material for: Design, Development, and Evaluation of an Automated Solution for Electronic Information Exchange Between Acute and Long-term Postacute Care Facilities: Design Science Research
Source: JMIR Form Res. 2023 Feb 17;7:e43758. doi: 10.2196/43758 (PMC9985001; doi:10.2196/43758)
Supplement: Multimedia Appendix 1 [file formative_v7i1e43758_app1.docx]

# **Appendix- 1**

## **CCD Generation**

CCD for a given patient is generated as a part of a Java block of code running parallelly to the Mirth tool.

### **CCD Generator**

### CCD Generator is a java with hibernate application which generates the CCD document. This application uses MDHT (Model Driven Health Tools library) Run-time libraries for generation of the Continuity of Care Document.

This application runs as a batch scheduler parallelly to the Mirth tool and checks the common database every 1 minute to check if any patient / message is ready for CCD generation. Once a patient / message is found the application would read all the relevant data points of that patient / message and generate the CCD.

The CCD XML output of the CCD Generator is stored in the common database in table PatientCCD and the CCD is also created as an XML file on the machine that it is running.

The class diagram for CCD Generator is depicted below.

**Figure 1:** CCD Generator Class Diagram


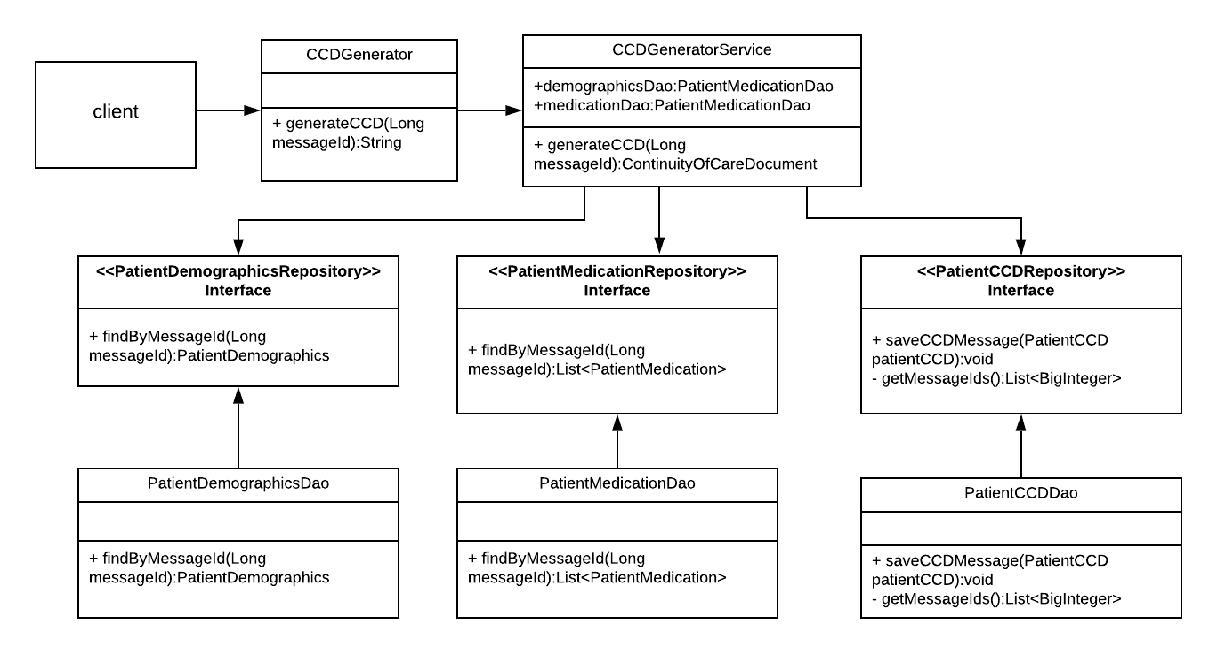


The invocation of the CCD Generator is done by the Task Manager with the following Syntax: CCDGenerator.generateCCD(Long messageId).

The CCDGenerator class generateCCD() method receives messageId and reads all the relevant data points of that patient / message and generates the CCD and persists the generated CCD document in the database and in the file system.
