# Supplementary material for: Design, Development, and Evaluation of an Automated Solution for Electronic Information Exchange Between Acute and Long-term Postacute Care Facilities: Design Science Research
Source: JMIR Form Res. 2023 Feb 17;7:e43758. doi: 10.2196/43758 (PMC9985001; doi:10.2196/43758)

# **Appendix- 2**

## **Data Model**

The tables that are used to satisfy the goals of this project, have been grouped by Subject Areas. Each Subject Area contains related tables. The subject areas that have been created for this solution include:

- Common Message Table: These tables are used for housekeeping of the raw messages and state transition of messages.
- HL7 Interface Related Tables: These tables are used for storing the information parsed from HL7 ADT A03 message
- EPIC Interface related tables: These tables are used to store the required information for to / from communication with Epic.
- PCC Interface related tables: These tables are used to store the required information for to/from communication with PCC.

The data model of each of the subject areas and the associated tables are detailed below.

## **Common Message Tables:**

The data model for the common message table along with the description and details of these tables are described in this section.

**Figure 1:** Common Message Subject Area


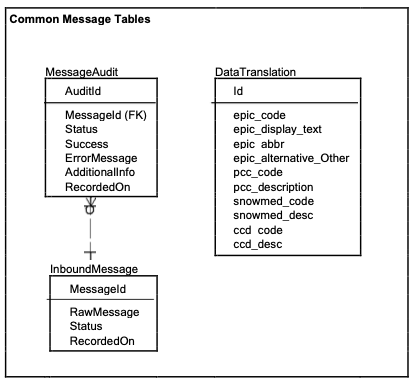


### **Table Name: InboundMessage**

This table holds all the incoming ADT A03 message. Every A03 message is associated with a MessageId which will be used in all the subsequent tables where ever applicable. The current status of the A03 message is available in the status column and is updated when the message moves through the work flow in the system.


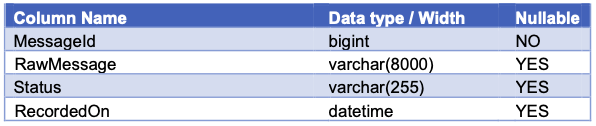


### **Table Name: DataTranslation**

This table holds the translation of data between different coding systems and different entities available in the system. It helps in data translation / codification when data has to be submitted to respective end points.


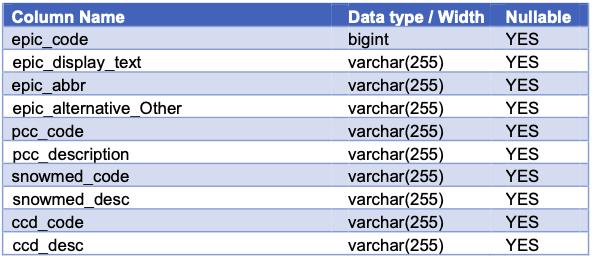


### **Table Name: MessageAudit**

This table is used for auditing the message (ADT A03), when the message moves between the components in the process flow. It can also provide information on the current status of the message and any errors that occurred when the message is being transformed between states.


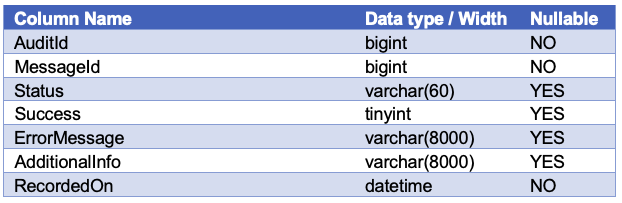


## **HL7 Interface related Tables**

The data model and the associated description of the HL7 related tables are documented in this section.

**Figure 2:** HL7 Interface Tables Subject Area

**
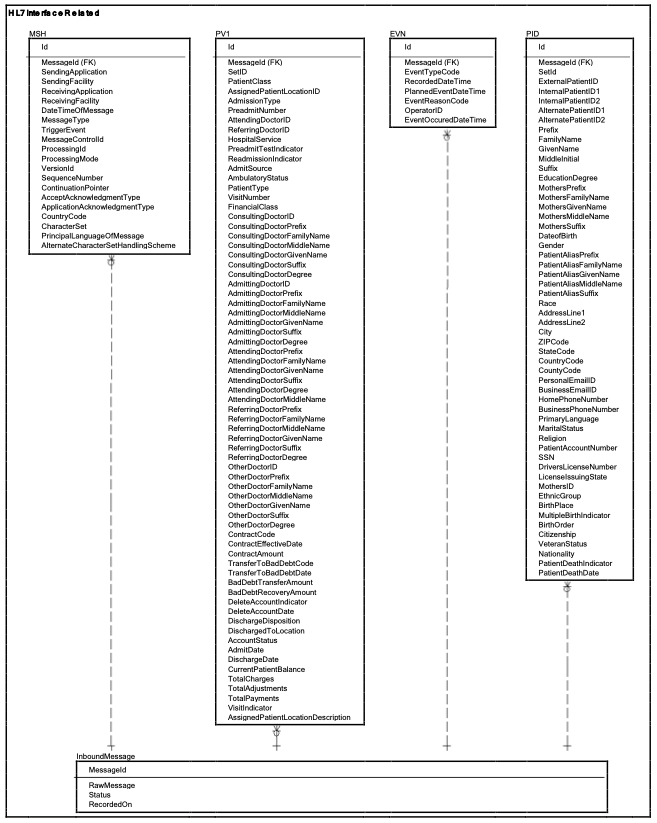
**

### **Table Name: MSH**

The ADT A03 message received, is parsed & the respective MSH segment and its elements are stored in this table for further processing / usage.

**
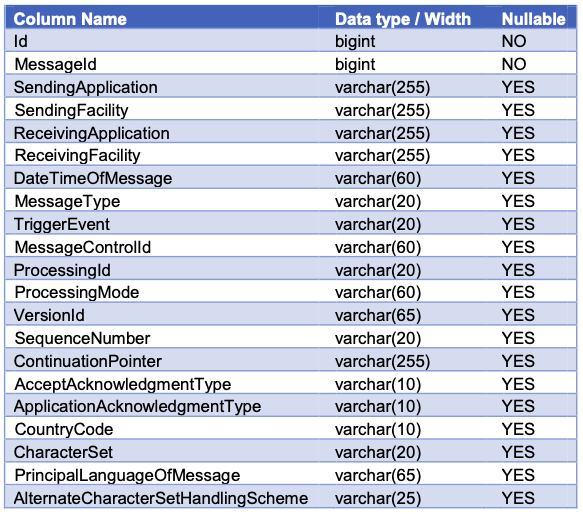
**

### **Table Name: EVN**

The ADT A03 message received, is parsed & the respective EVN segment and its elements are stored in this table for further processing / usage.

**
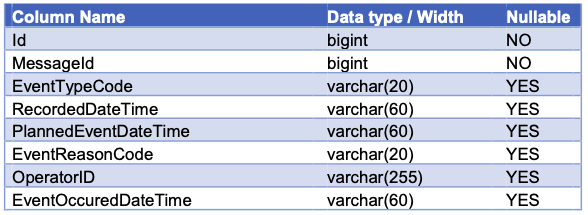
**

### **Table Name: PID**

The ADT A03 message received, is parsed & the respective PID segment and its elements are stored in this table for further processing / usage.


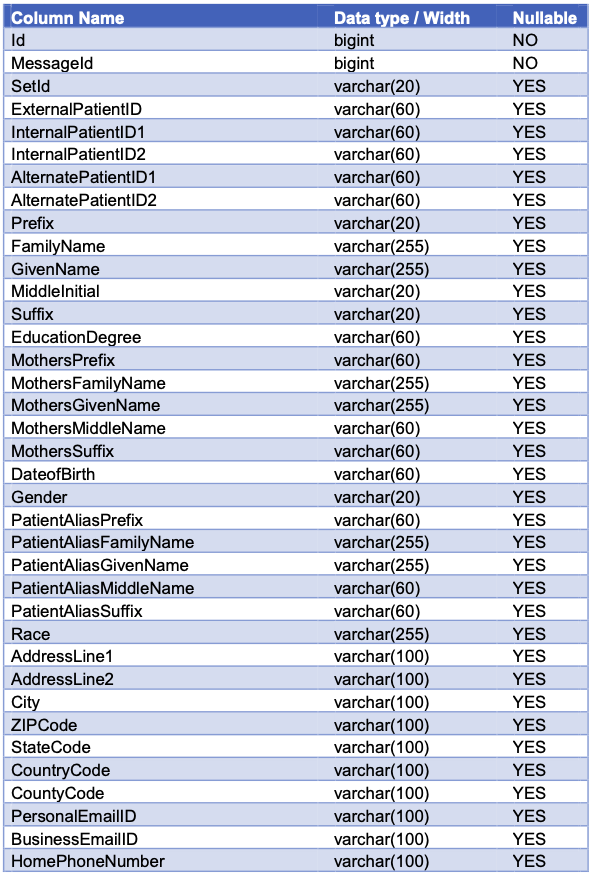


### **
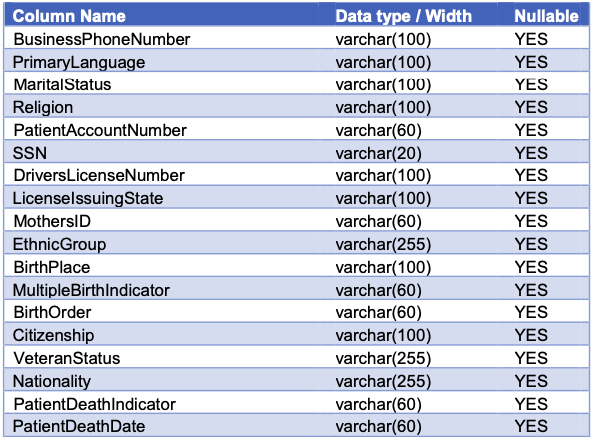
**

### **Table Name: PV1**

The ADT A03 message received, is parsed & the respective PV1 segment and its elements are stored in this table for further processing / usage.


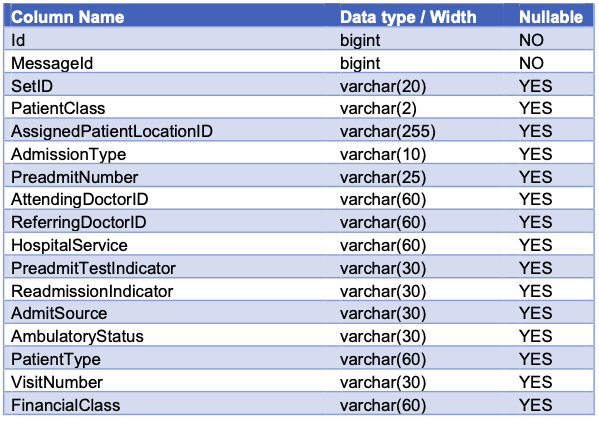


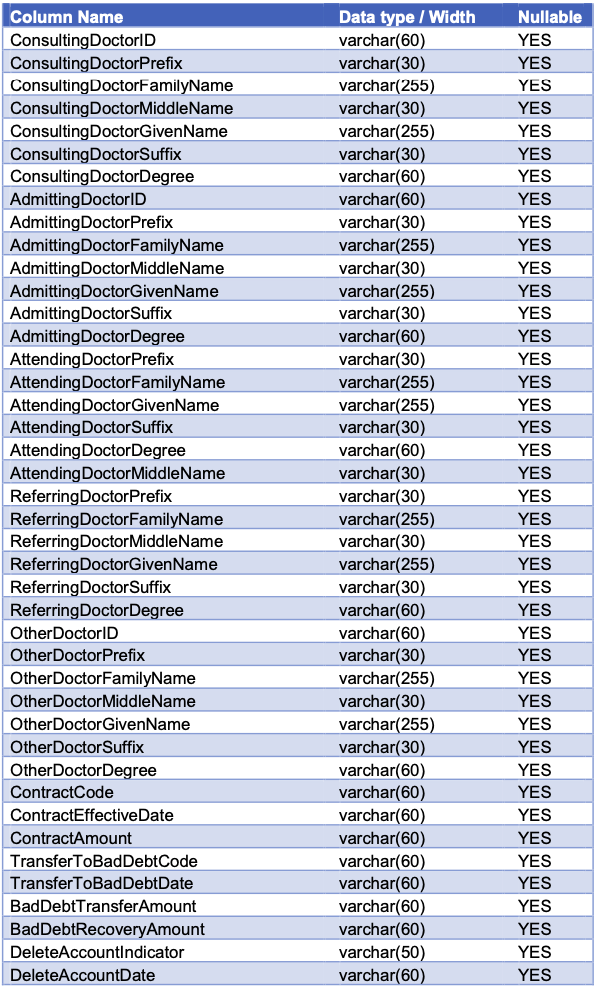


## **
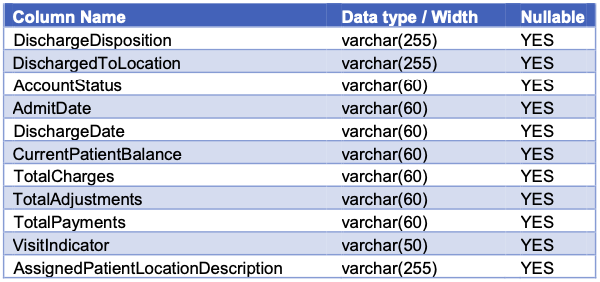
**

## **EPIC Interface related tables**

This section gives additional information on the tables that are used to exchange messages between Mirth Connect and Epic. This section contains the data model along with the description of tables.

**Figure 3:** HL7 Interface Tables Subject Area


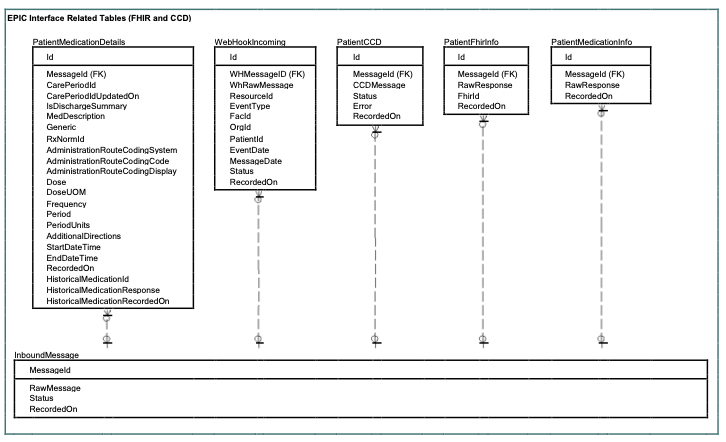


### **Table Name: PatientFhirInfo**

This table holds the FHIR id of the patient once retrieved.


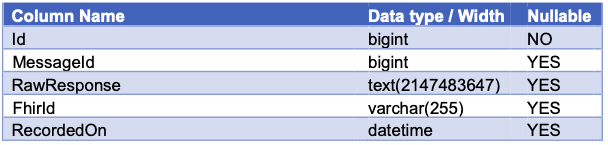


### **Table Name: PatientMedicationInfo**

This table stores the Raw FHIR Medications message response received.


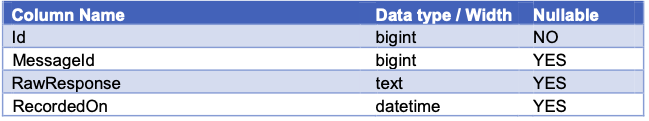


### **Table Name: PatientMedicationDetails**

The FHIR medications pf the patient are parsed & the respective elements are stored in this table. It also stores the Historical medication id that is received as response when the medications are posted to Post Historical Medications End point.


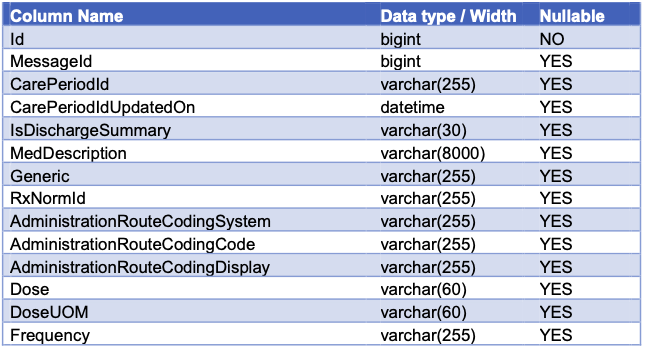


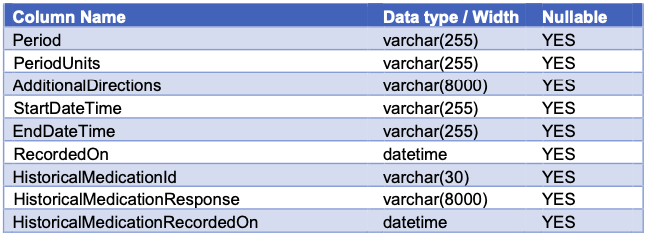


### **Table Name: PatientCCD**

This table holds the CCD document generated for a given patient


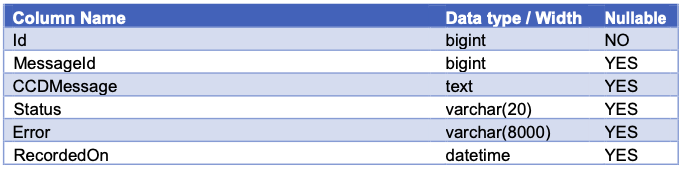


## **PCC Interface related tables**

This section gives additional information on the tables that are used to exchange messages between Mirth Connect and PCC. This section contains the data model along with the description of tables.

**Figure 4:** PCC Interface Subject Area


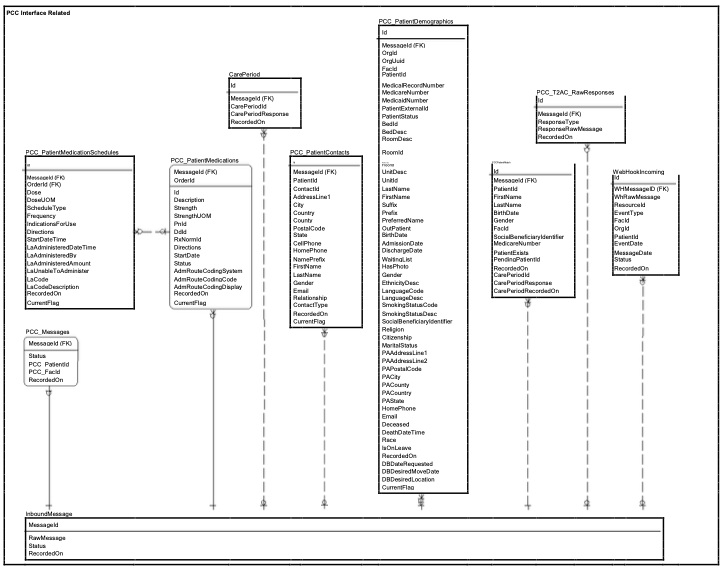


### **Table Name: PCCPatientMatch**

This table holds the patient details as received, from the PCC system based on the patient search request submitted. If the patient is found, then all the respective patient details are stored, else the patient information is submitted as pending patient and the respective Pending patient id acknowledgement is stored for further mapping once the patient is synchronized. It also holds the respective care period of the patient which is updated when the information is available at the respective logical step in the work flow process.

###
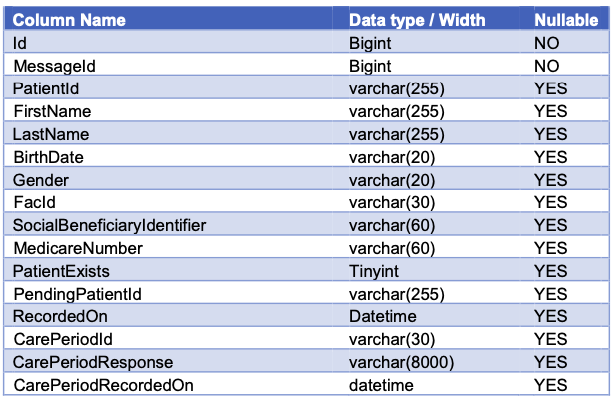


### **Table Name: WebHookIncoming**

This table holds the incoming data received based on the web hook call. Based on the Event Type of the incoming web hook message respective work flow process is triggered.


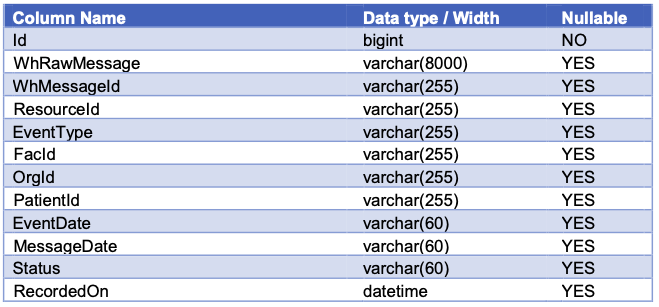


### **Table Name: PCC_Messages**

This table hold information of the incoming web hook messages where the event type = patient.discharge and is used for further processing in the work flow


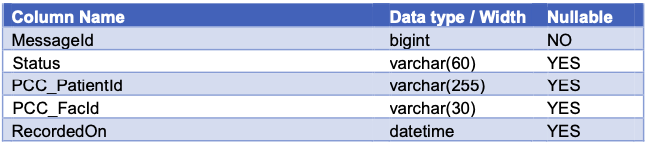


### **Table Name: PCC_PatientDemographics**

This table holds the patient demographics as retrieved from the PCC System


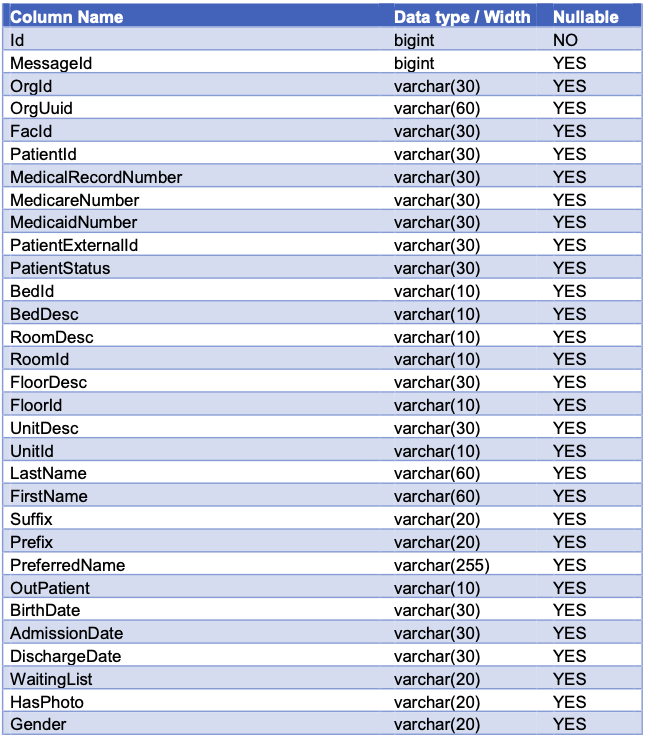


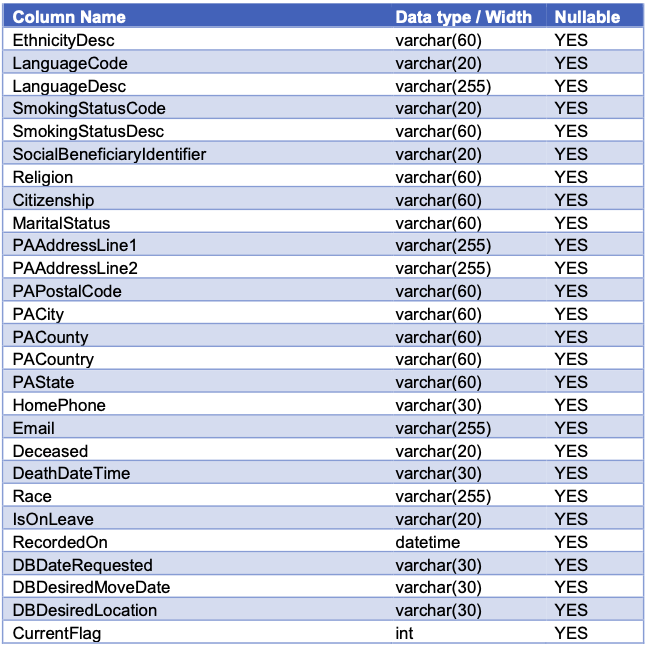


### **Table Name: PCC_PatientContacts**

This table holds the patient contact information as retrieved from the PCC System


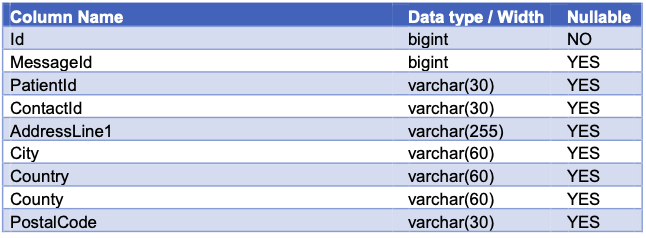


### **
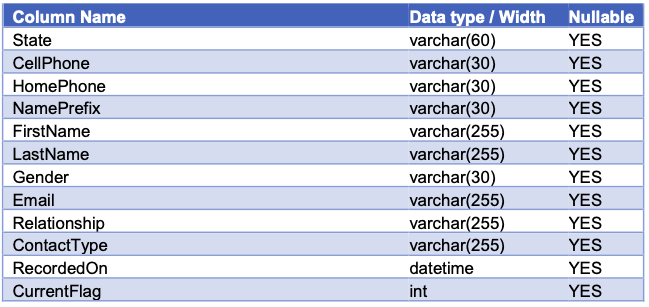
**

### **Table Name: PCC_PatientMedications**

This table holds the patient medications as retrieved from the PCC system


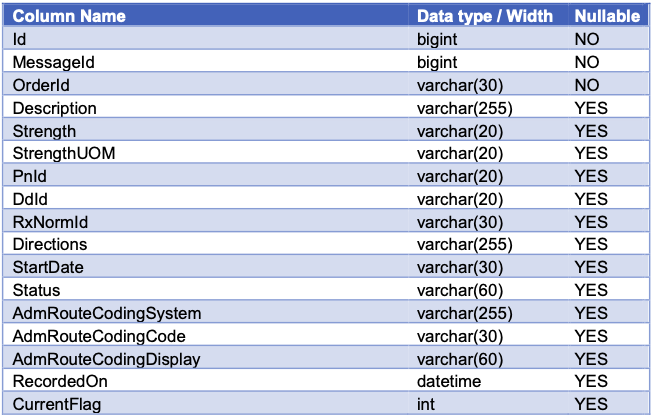


### **Table Name: PCC_PatientMedicationSchedules**

This table holds the patient medications scheduled as retrieved from the PCC system


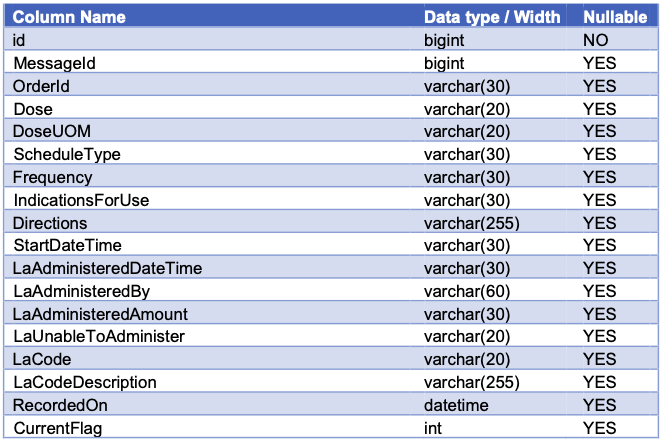


### **Table Name: PCC_T2AC_RawResponses**

This table holds the raw / actual response received from the PCC system when queried for demographics / medications of a patient.


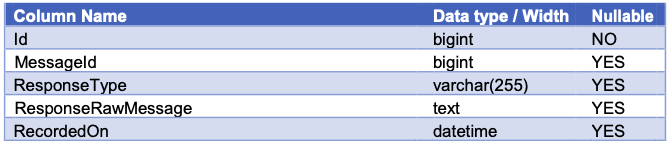

Supplement: Multimedia Appendix 2 [file formative_v7i1e43758_app2.docx]
