# Supplementary material for: Design, Development, and Evaluation of an Automated Solution for Electronic Information Exchange Between Acute and Long-term Postacute Care Facilities: Design Science Research
Source: JMIR Form Res. 2023 Feb 17;7:e43758. doi: 10.2196/43758 (PMC9985001; doi:10.2196/43758)

# **Appendix- 3**

## **Mirth Channel Description**

This section describes the Mirth Channels that have been configured to satisfy the business and technical needs of this interoperability solution. The interaction and sequencing of the Mirth Channels are described in the UML Action Diagram section.

The Dashboard view of all channels is depicted below, followed by description of all the channels used in the solution.

**Figure 1:** Mirth Connect Dashboard of All Channels Created for this Solution


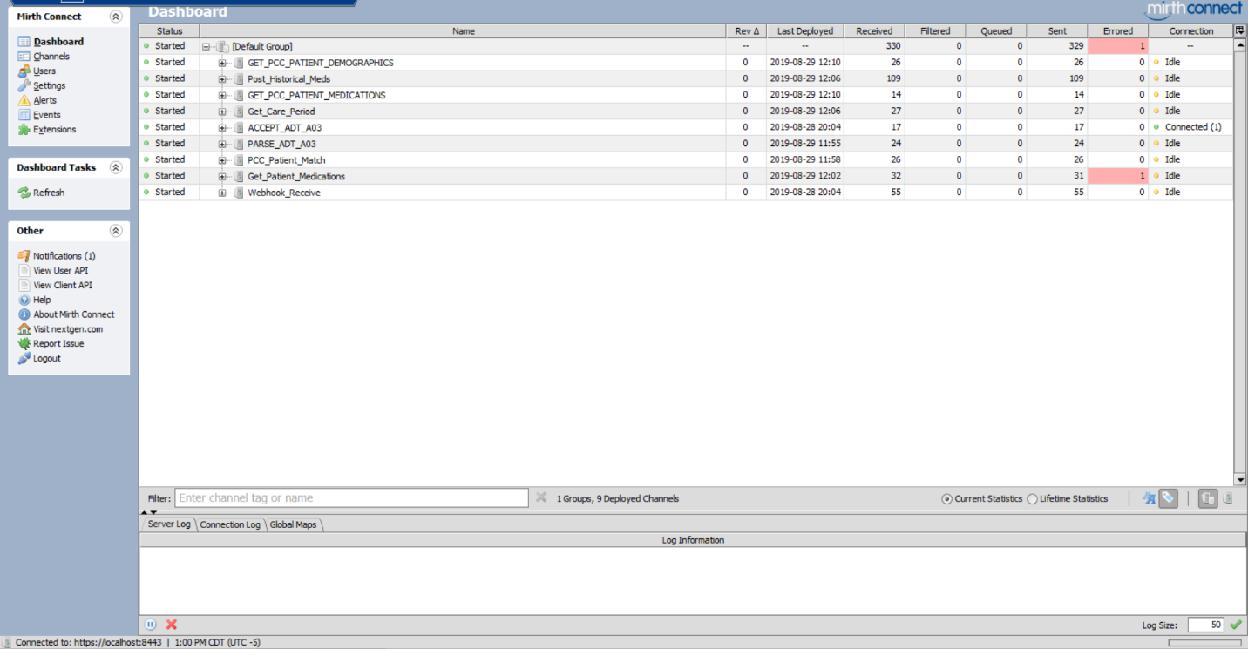


### **Channel Name: ACCEPT_ADT_A03**

This channel listens on TCP/IP port 7575 and accepts all incoming HL7 messages. It accepts the message and stores in the Database for further processing. If it receives a valid ADT A03 event type an “AA” (Accepted) acknowledgement is sent back to the submitter else “AR” (Rejected) acknowledgement is sent back to the submitter. If there are any errors encountered while accepting the message an “AE” (Error) acknowledgement is sent back. Transformations are used to identify the event type of the message.

|  | Source | Incoming HL7 Message |
| --- | --- | --- |
|  | Destination | SQL Server Database |
|  | Tables Used | InboundMessage |

**Figure 2:** ACCEPT_ADT_A03 Channel – Source Definition


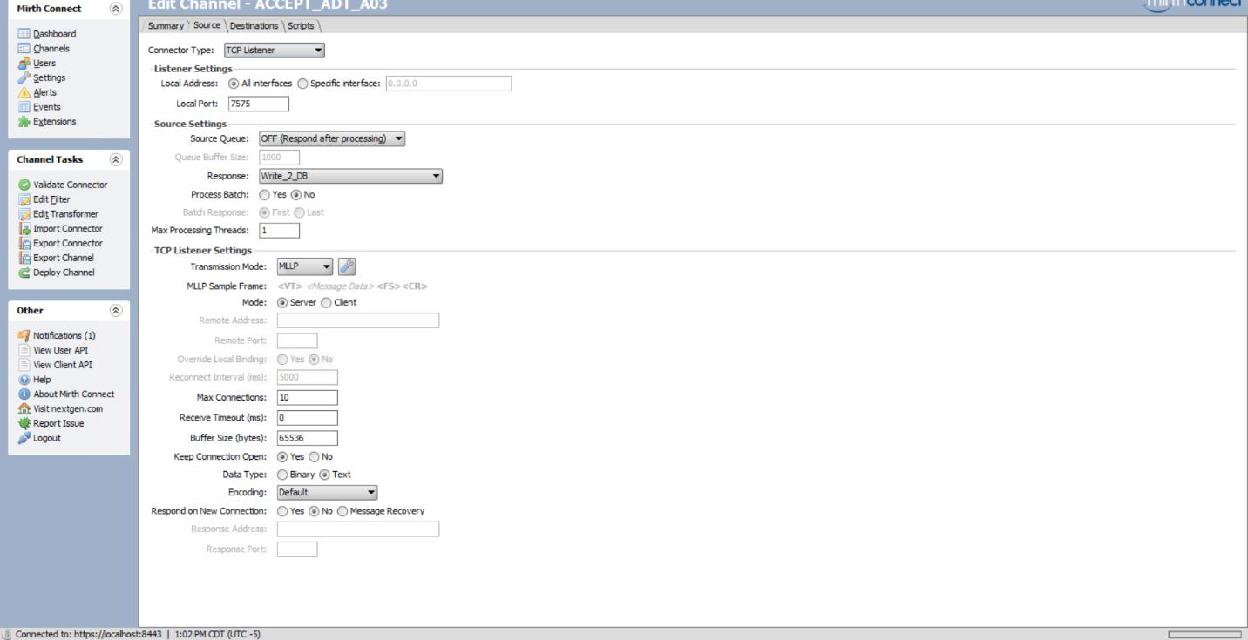


**Figure 3:** ACCEPT_ADT_A03 Channel – Destination Definition


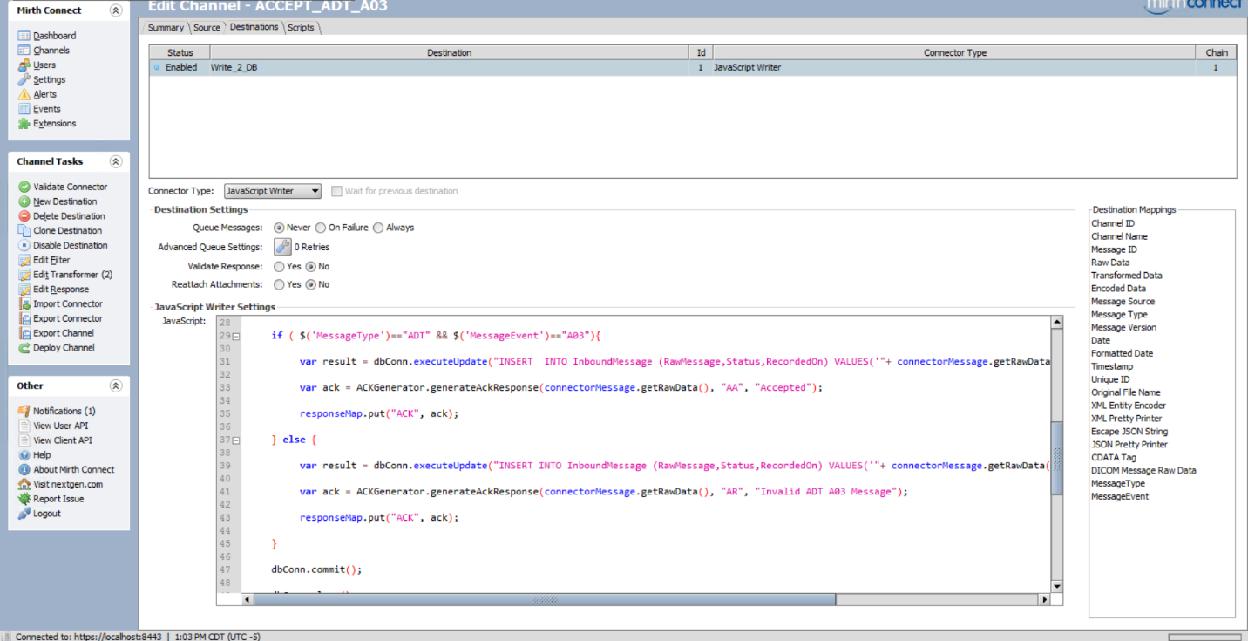


### **Channel Name: PARSE_ADT_A03**

This channel polls the database at regular intervals and checks for HL7 ADT A03 messages that are received successfully. It picks up such messages and parses them to store the HL7 segments and their elements into respective tables for further processing.

|  | Source | SQL Server Database Table InboundMessage |
| --- | --- | --- |
|  | Destination | SQL Server Database Tables (MSH, PVN, PID, PV1, MessageAudit) |
|  | Tables Used | InboundMessage, MSH, EVN, PID, PV1, MessageAudit |

**Figure 4:** PARSE_ADT_A03 Channel – Source Definition


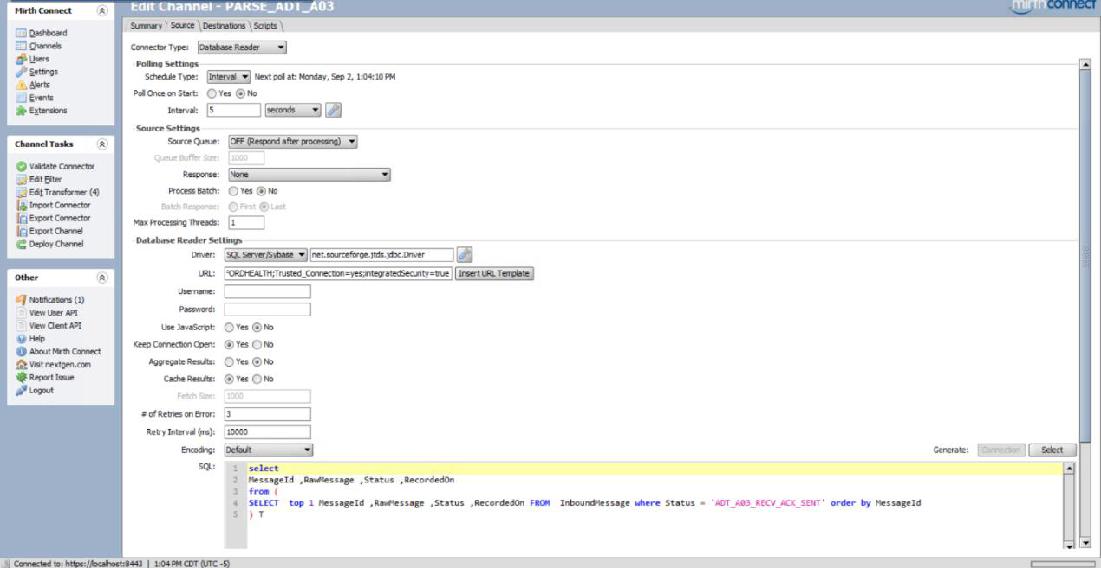


**Figure 5:** PARSE_ADT_A03 Channel – Destination Definition


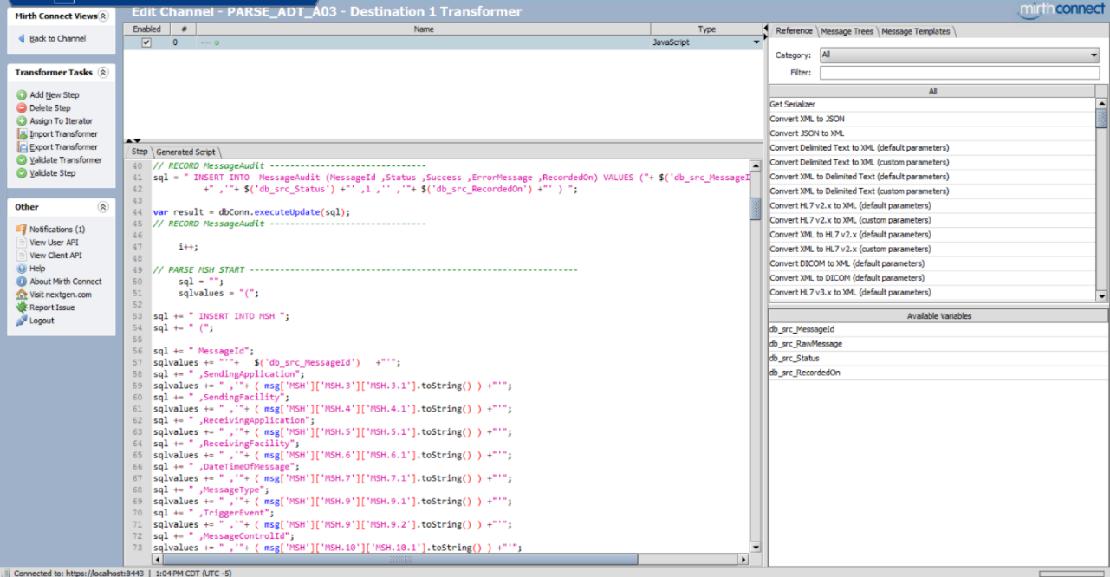


### **Channel Name: PCC_PATIENT_MATCH**

This channel checks if the patient is existing in PCC, if the patient exists it would store the response match information else it would post the patient information to pending patients’ queue and also store the pending Patient Id for further reference.

|  | Source | SQL Server Database Table InboundMessage | | | |
| --- | --- | --- | --- | --- | --- |
|  | Destination | SQL Server Database Tables (PCCPatientMatch, MessageAudit) | | | |
|  | Tables Used | InboundMessage, PCCPatientMatch, MessageAudit | | | |
|  | Endpoints Used | Get Token |  | | |
|  |  | Post Patient Match | |  | |
|  |  | Post Pending Patients | | | |
|  |  |  |  |  |  |

**Figure 6:** PCC_PATIENT_MATCH Channel – Source Definition


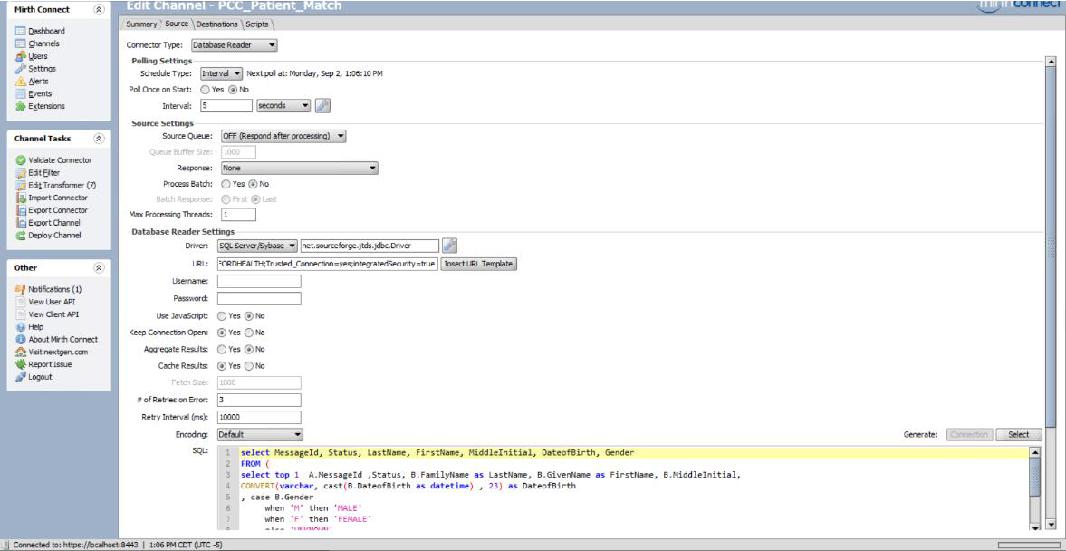


**Figure 7:** PCC_PATIENT_MATCH Channel – Destination Definition


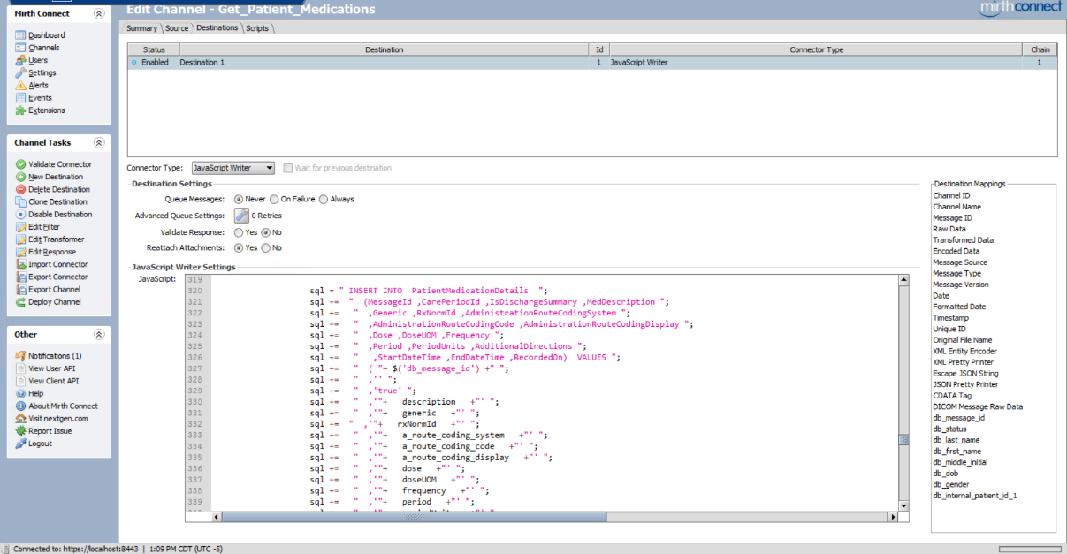


### **Channel Name: GET_PATIENT_MEDICATIONS**

This channel retrieves the respective FHIR Id of the patient & also the respective medications of the patient from the FHIR resource.

|  | Source | SQL Server Database Table InboundMessage | | |
| --- | --- | --- | --- | --- |
|  | Destination | SQL Server Database Tables (PatientFHIRInfo, PatientMedicationInfo, | | |
|  |  | PatientMedicationDetails, MessageAudit) | | |
|  | Tables Used | InboundMessage, PatientFhirInfo, PatientMedicationInfo, | | |
|  |  | PatientMedicationDetails, MessageAudit | | |
|  | Endpoints Used | Get Patient FHIR ID | | |
|  |  |  |  |  |
|  |  | Get Medication Statement | | |
|  |  |  |  |  |

**Figure 8:** Get_Patient_Medications Channel – Source Definition


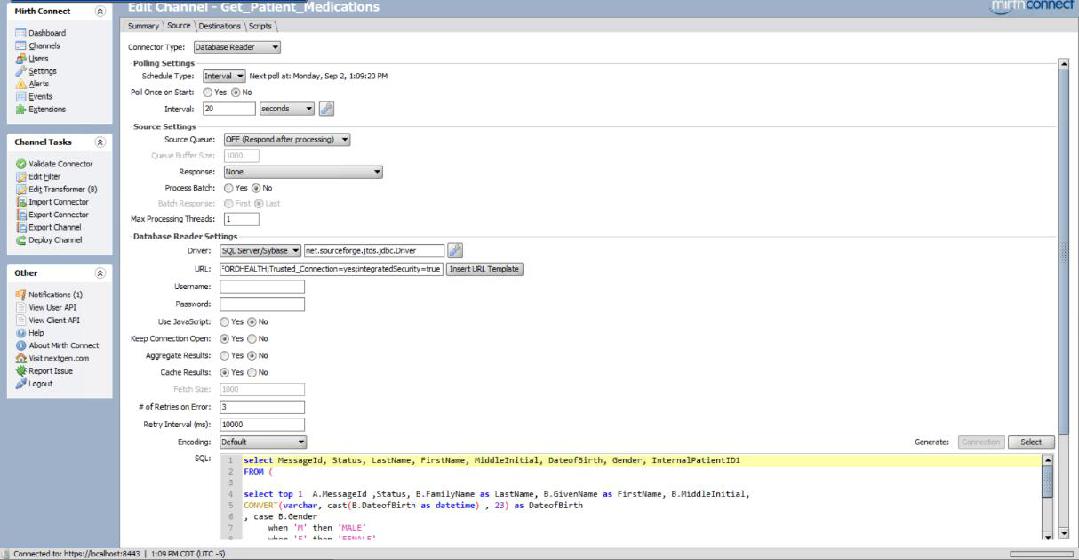


**Figure 9:** Get_Patient_Medications Channel – Destination Definition


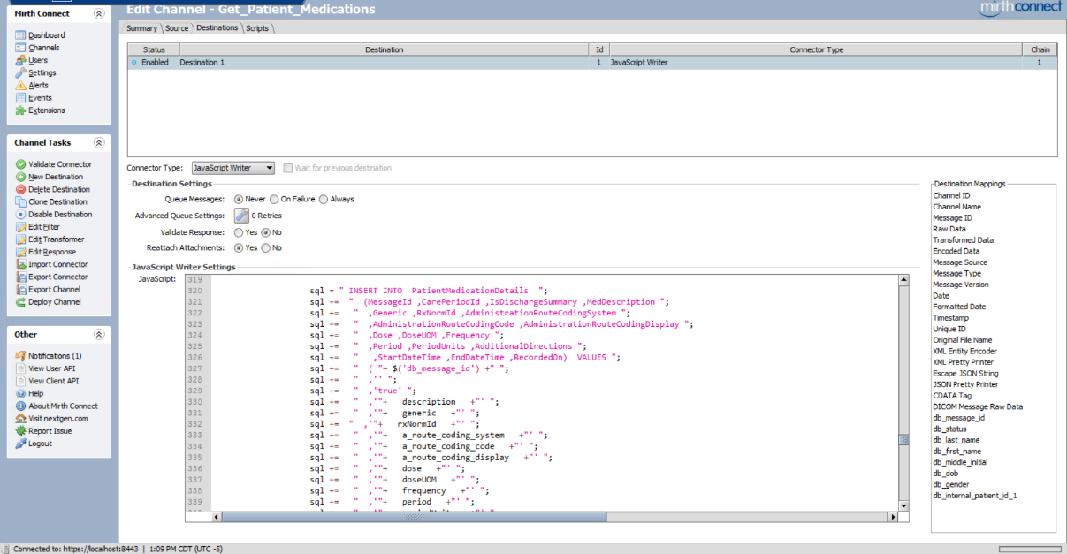


### **Channel Name: GET_CARE_PERIOD**

This channel retrieves the care periods of the respective patient and stores them against each patient information.

|  | Source | SQL Server Database Table InboundMessage | |
| --- | --- | --- | --- |
|  | Destination | SQL Server Database Tables (PCCPatientMatch, | |
|  |  | PatientMedicationDetails, MessageAudit) | |
|  | Tables Used | InboundMessage, PCCPatientMatch, PatientMedicationDetails, | |
|  |  | MessageAudit | |
|  | Endpoints Used | Create Care Period in PCC for a Patient | |
|  |  |  |  |

Figure 10: GET_CARE_PERIOD Channel – Source Definition


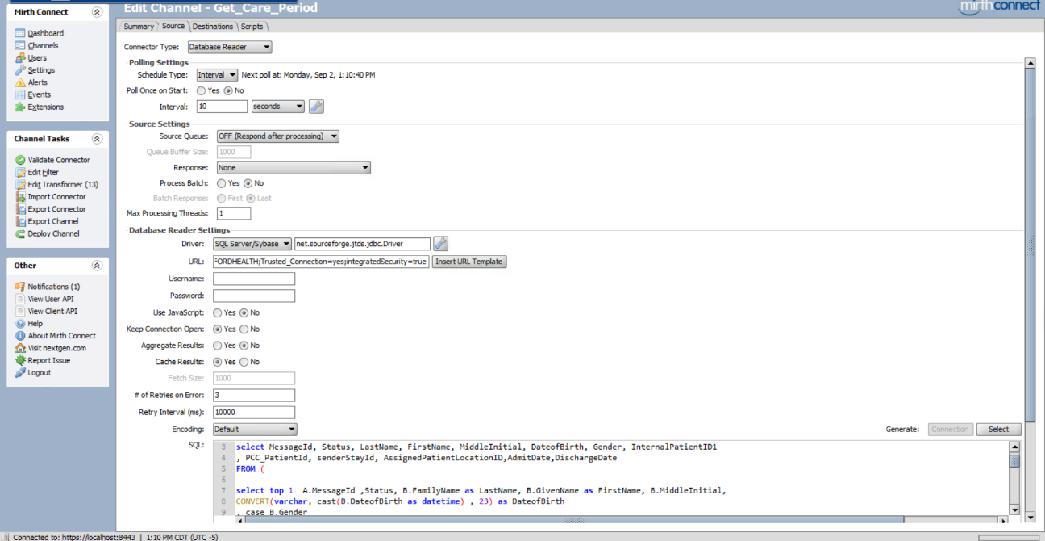


Figure 11: GET_CARE_PERIOD Channel – Destination Definition


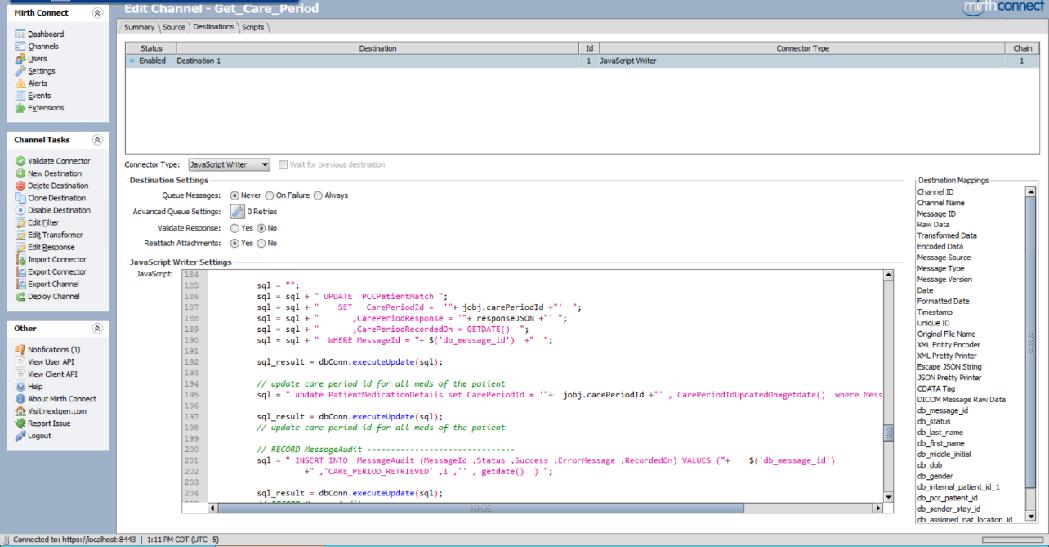


### **Channel Name: POST_HISTORICAL_MEDS**

This channel submits the Medications of the patient with their respective care period Ids. Once submitted the historicalMedicationId received in response is stored against respective submission of the medications.

|  | Source | SQL Server Database Table InboundMessage | |
| --- | --- | --- | --- |
|  | Destination | SQL Server Database Tables (PatientMedicationDetails, | |
|  |  | MessageAudit) | |
|  | Tables Used | InboundMessage, PatientMedicationDetails, MessageAudit | |
|  | Endpoints Used | Post Historical Medication for a Patient in PCC | |
|  |  |  |  |

Figure 12: Post_Historical_Meds Channel – Source Definition


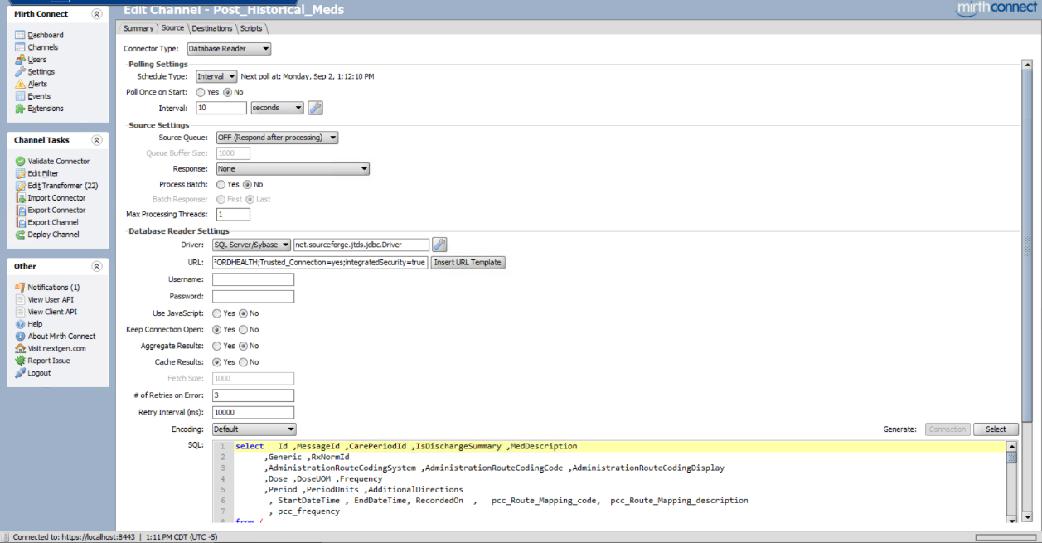


Figure 13: Post_Historical_Meds Channel – Destination Definition


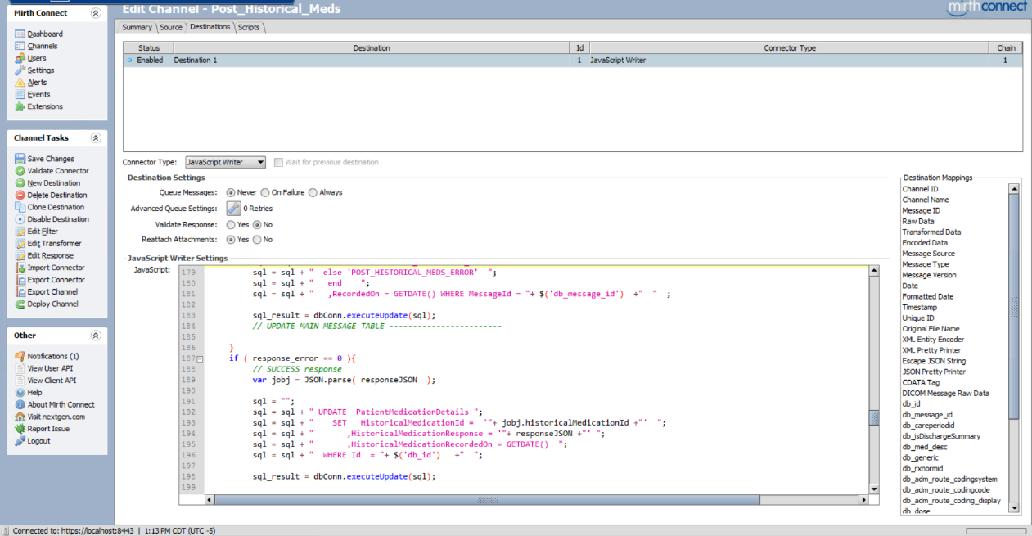


### Channel Name: GET_PCC_PATIENT_DEMOGRAPHICS

This channel retrieves the Patient Demographics from the PCC system, all the attributes are stored and used in CCD generation.

|  | Source | SQL Server Database Table InboundMessage | |
| --- | --- | --- | --- |
|  | Destination | SQL Server Database Tables (PCC_Messages, | |
|  |  | PCC_PatientDemographics, PCC_PatientContacts, | |
|  |  | PCC_T2AC_RawResponses, MessageAudit) | |
|  | Tables Used | InboundMessage, PCC_Messages, PCC_PatientDemographics, | |
|  |  | PCC_PatientContacts, PCC_T2AC_RawResponses, MessageAudit | |
|  | Endpoints Used | Get Patient Demographics from PCC | |
|  |  |  |  |

Figure 14: GET_PCC_PATIENT_DEMOGRAPHICS Channel – Source Definition


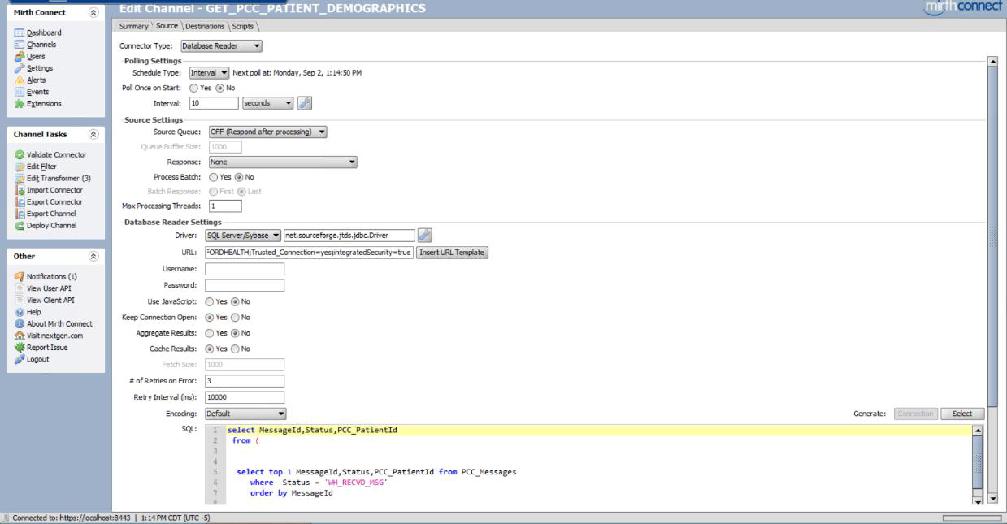


Figure 15: GET_PCC_PATIENT_DEMOGRAPHICS Channel – Destination Definition


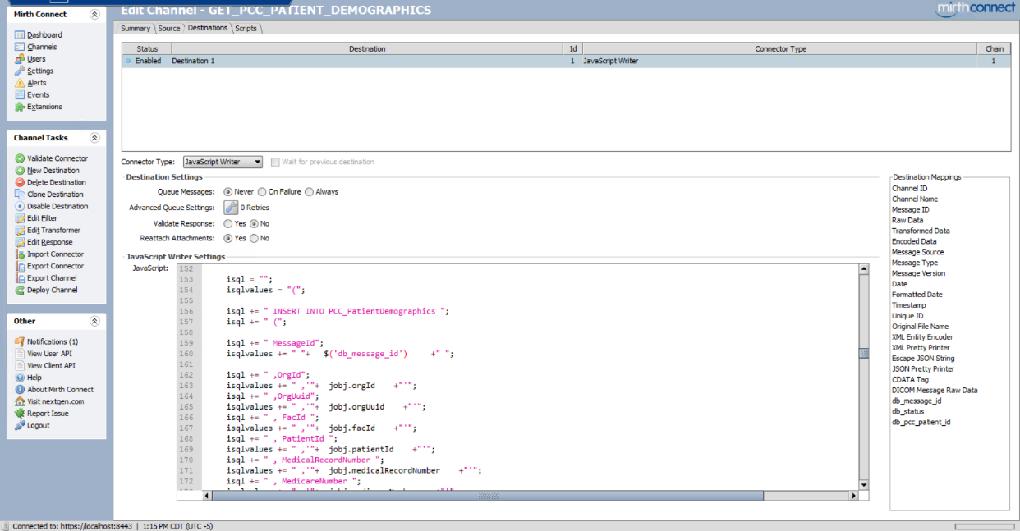


### Channel Name: GET_PCC_PATIENT_MEDICATIONS

This channel retrieves the Patient Medications from the PCC system, all the attributes are stored and used in CCD generation.

|  | Source | SQL Server Database Table InboundMessage | |
| --- | --- | --- | --- |
|  | Destination | SQL Server Database Tables (, PCC_Messages, | |
|  |  | PCC_PatientMedications, PCC_PatientMedicationSchedules, | |
|  |  | PCC_T2AC_RawResponses, MessageAudit) | |
|  | Tables Used | InboundMessage, , PCC_Messages, PCC_PatientMedications, | |
|  |  | PCC_PatientMedicationSchedules, PCC_T2AC_RawResponses, | |
|  |  | MessageAudit | |
|  | Endpoints Used | Get Medications from PCC | |
|  |  |  |  |

Figure 16: Get_Patient_Medications Channel – Source Definition


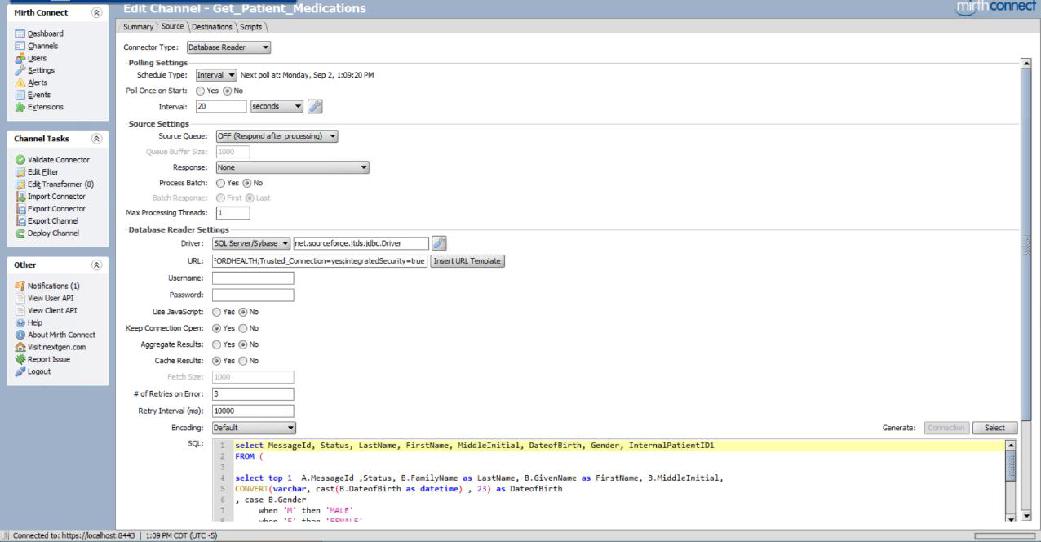


Figure 17: Get_Patient_Medications Channel – Destination Definition


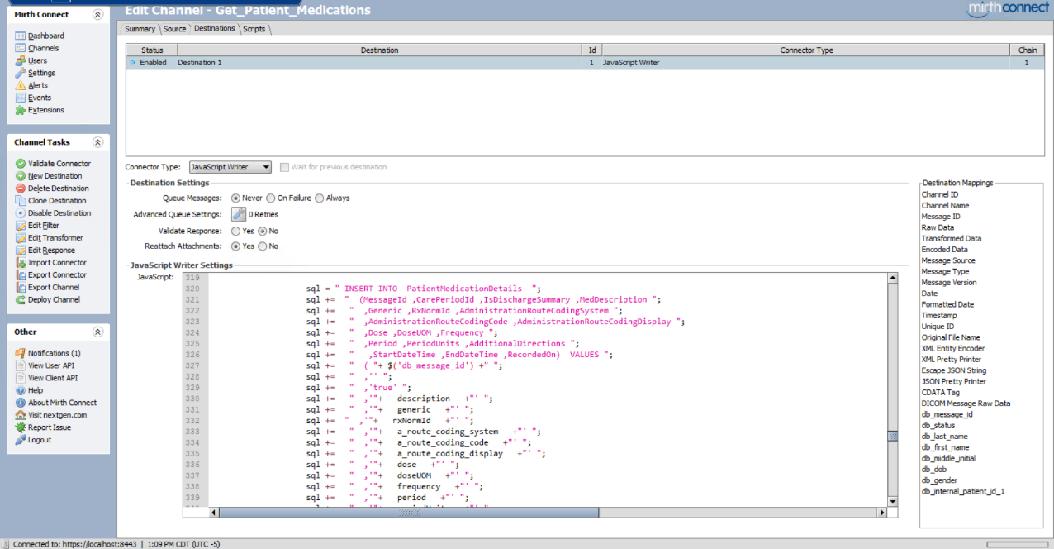


### Channel Name: WEBHOOK_RECEIVE

This channel listens on http port 3444 and accepts incoming webhook messages. A proxy software runs parallel to mirth which listens on http port 3443 which is secure and acts as the interface to the outside world. The webhook messages get submitted to the proxy software on 3443 and the proxy software redirects the messages internally to mirth web port 3444. The incoming web hook message is parsed and stored in DB for further processing, appropriate tables are updated based on the EventType value retrieved from the incoming webhook message.

|  | Source | Incoming Webhook Message |
| --- | --- | --- |
|  | Destination | SQL Server Database Tables WebHookIncoming, InboundMessage, |
|  |  | PCCPatientMatch, MessageAudit, PCC_Messages) |
|  | Tables Used | WebHookIncoming, InboundMessage, PCCPatientMatch, |
|  |  | MessageAudit, PCC_Messages |
|  | Endpoints Used | N/A |
|  |  |  |

Figure 18: Webhook_Receive Channel – Source Definition


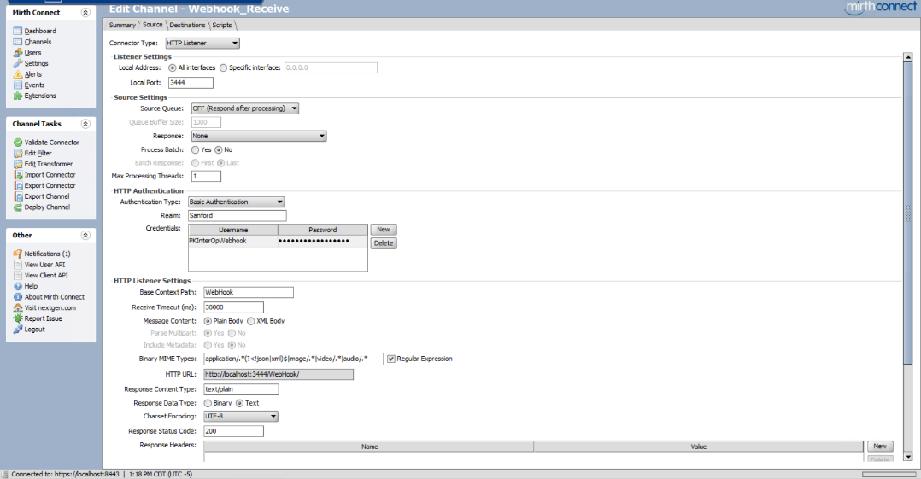


Figure 19: Webhook_Receive Channel – Destination Definition


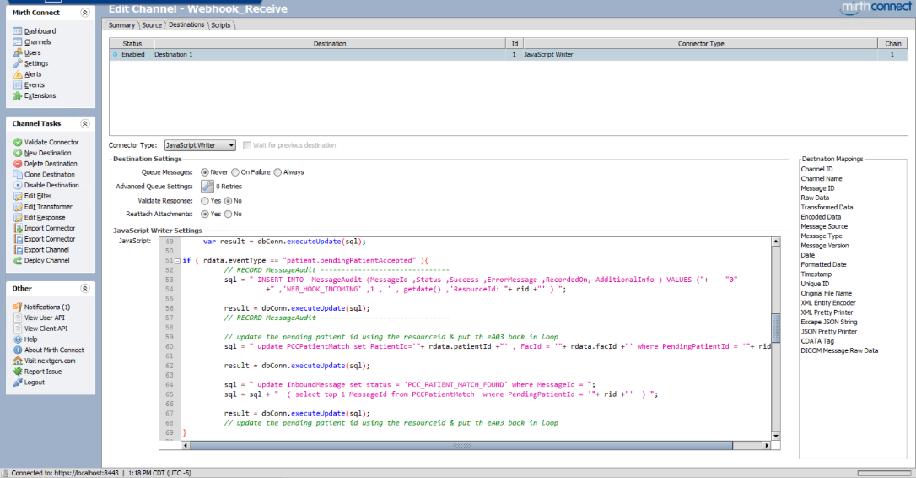

Supplement: Multimedia Appendix 3 [file formative_v7i1e43758_app3.docx]
