# Supplementary material for: Design, Development, and Evaluation of an Automated Solution for Electronic Information Exchange Between Acute and Long-term Postacute Care Facilities: Design Science Research
Source: JMIR Form Res. 2023 Feb 17;7:e43758. doi: 10.2196/43758 (PMC9985001; doi:10.2196/43758)
Supplement: Multimedia Appendix 4 [file formative_v7i1e43758_app4.pdf]

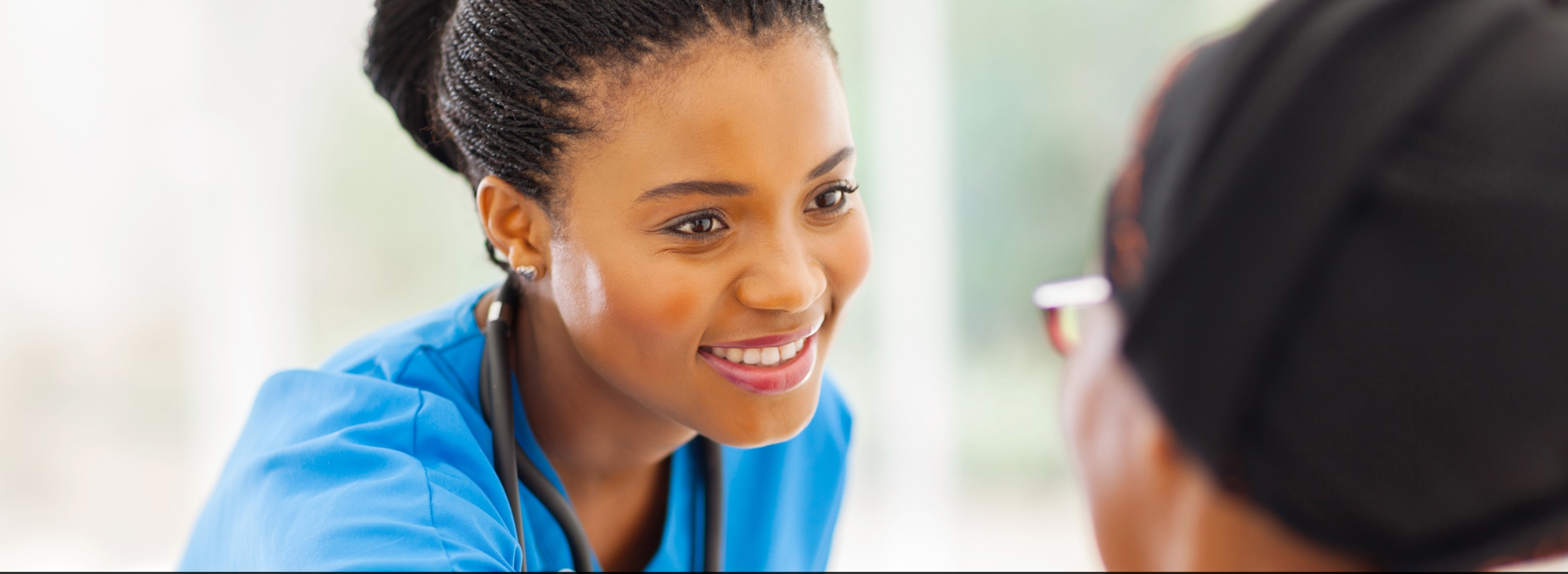

Epic Discharge Workflow

A patient at a acute care hospital is discharged to a skilled nursing home.

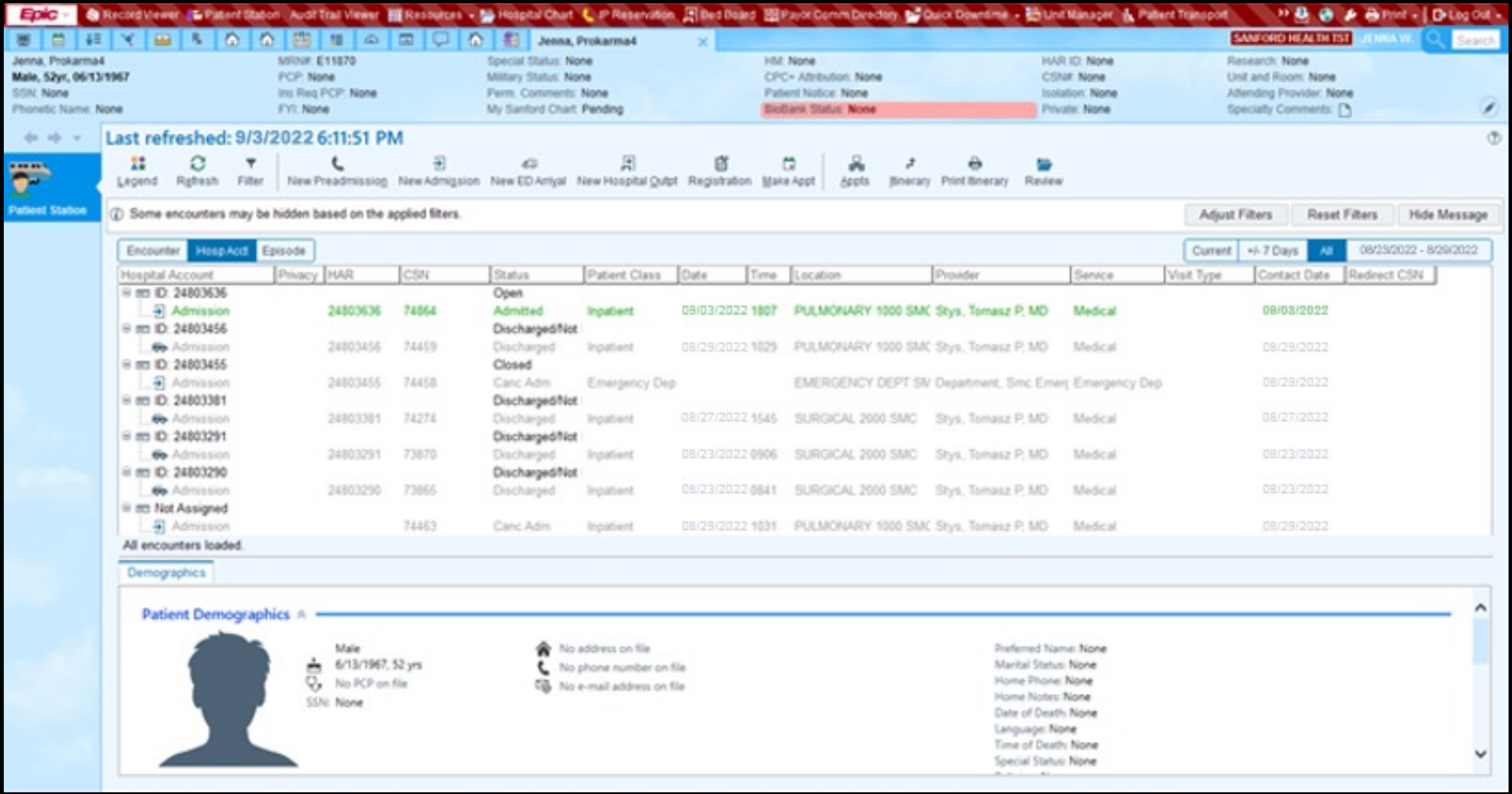

Patient is in acute care facility

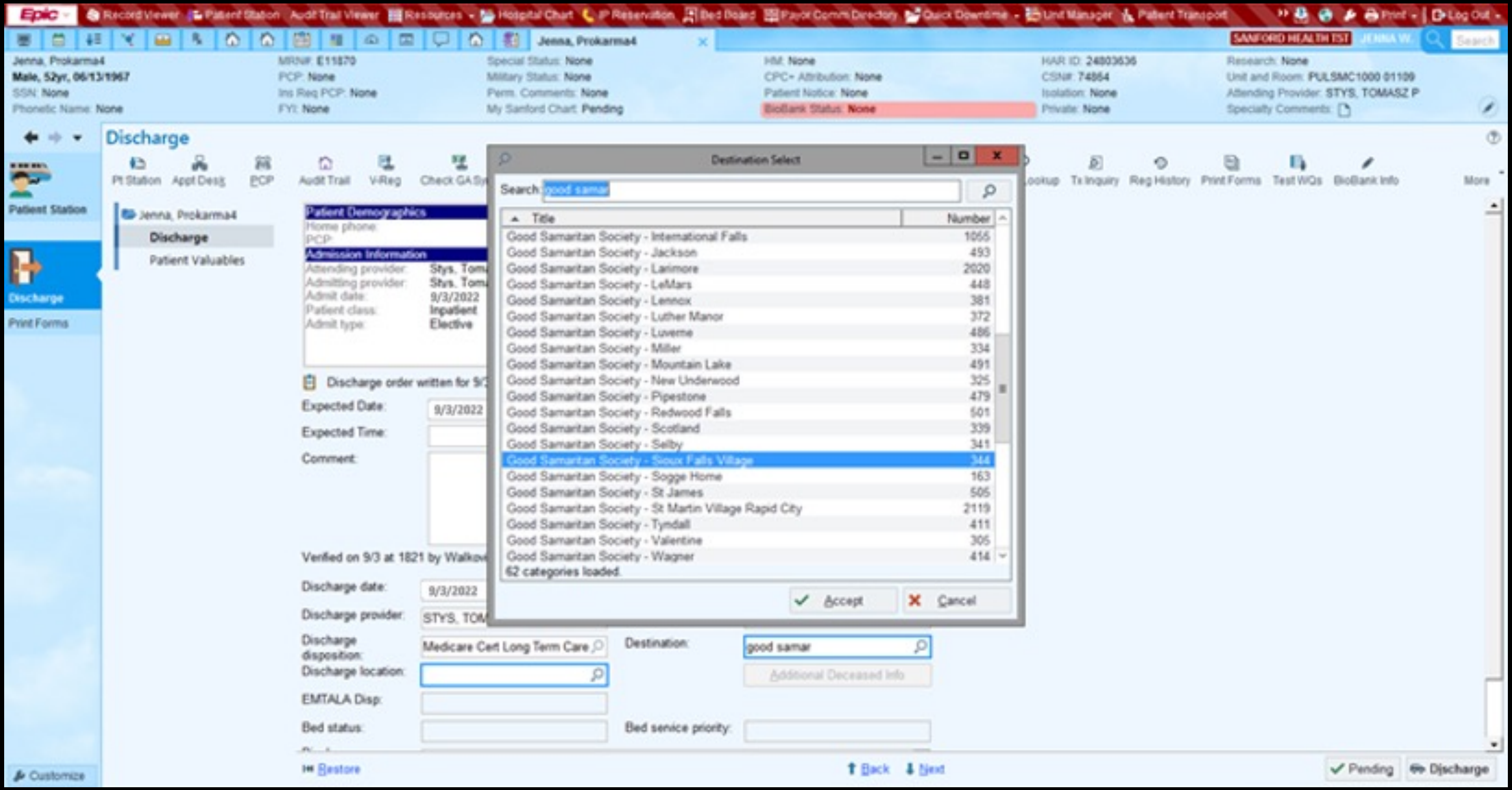

Discharge date, time, disposition, and destination are entered.

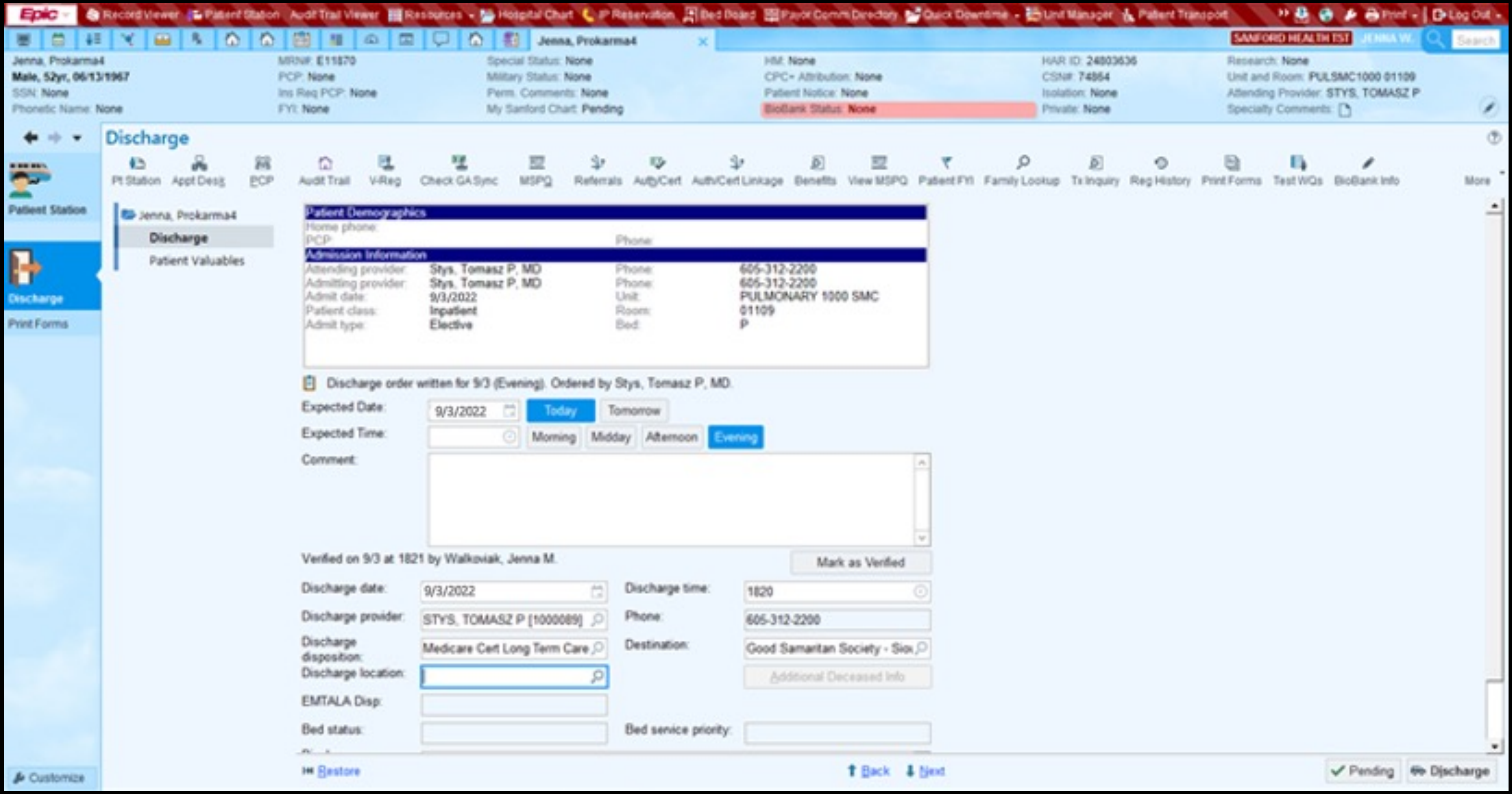

Patient has been discharged from acute care hospital and is enroute to skilled nursing facility

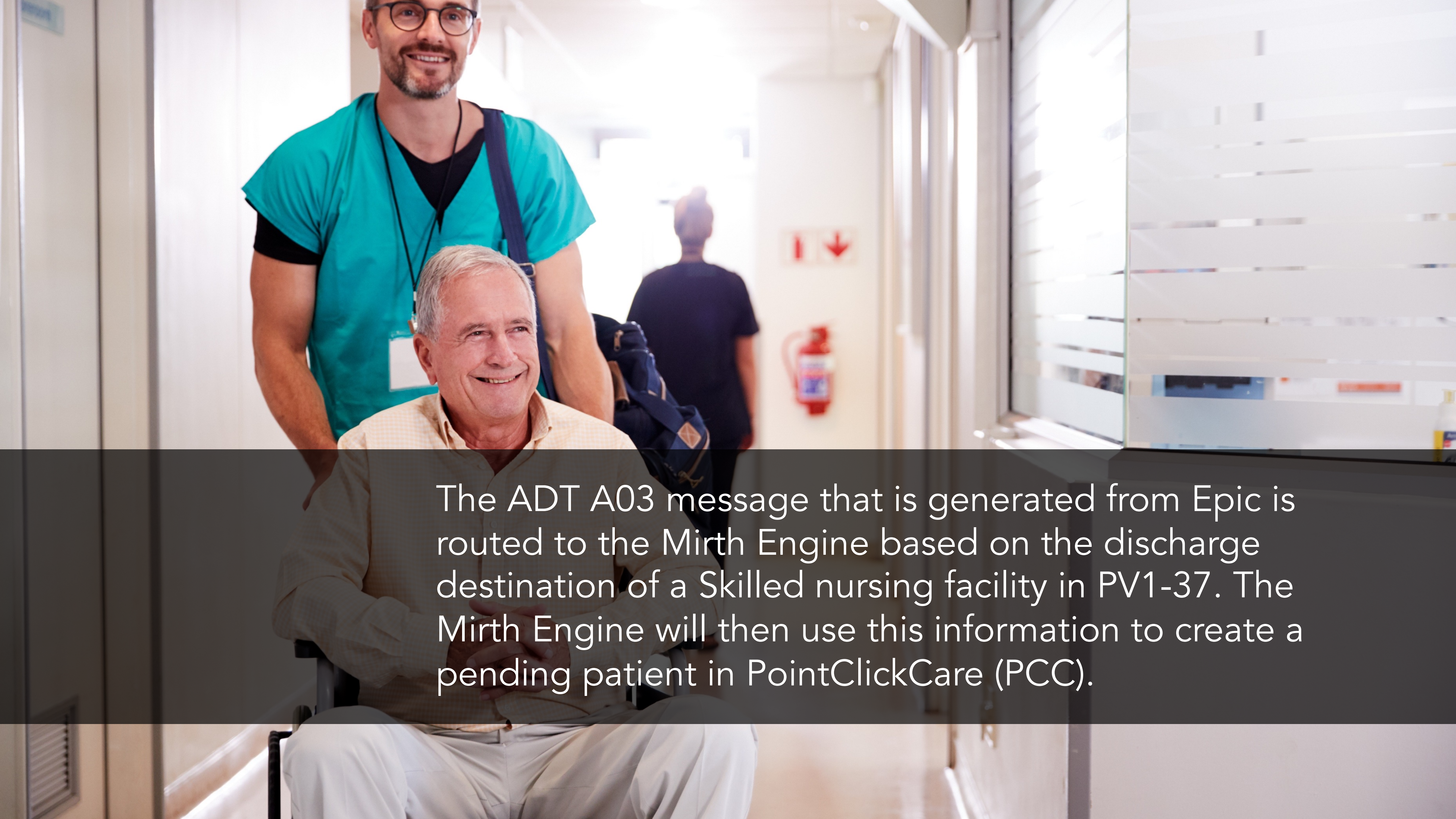

The ADT A03 message that is generated from Epic is routed to the Mirth Engine based on the discharge destination of a Skilled nursing facility in PV1-37. The Mirth Engine will then use this information to create a pending patient in PointClickCare (PCC).

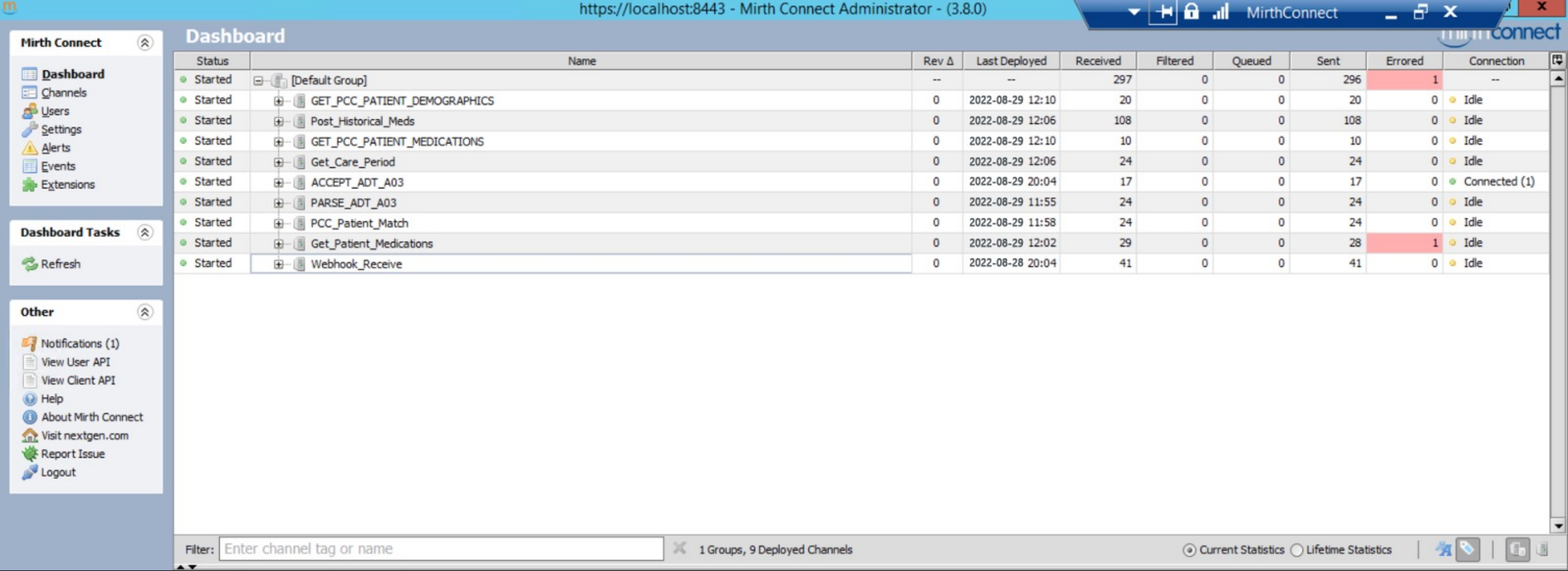

Status	Name	Rev Δ	Last Deployed	Received	Filtered	Queued	Sent	Errored	Connection
Started	[Default Group]	--	--	297	0	0	296	1	--
Started	GET_PCC_PATIENT_DEMOGRAPHICS	0	2022-08-29 12:10	20	0	0	20	0	Idle
Started	Post_Historical_Meds	0	2022-08-29 12:06	108	0	0	108	0	Idle
Started	GET_PCC_PATIENT_MEDICATIONS	0	2022-08-29 12:10	10	0	0	10	0	Idle
Started	Get_Care_Period	0	2022-08-29 12:06	24	0	0	24	0	Idle
Started	ACCEPT_ADT_A03	0	2022-08-29 20:04	17	0	0	17	0	Connected (1)
Started	PARSE_ADT_A03	0	2022-08-29 11:55	24	0	0	24	0	Idle
Started	PCC_Patient_Match	0	2022-08-29 11:58	24	0	0	24	0	Idle
Started	Get_Patient_Medications	0	2022-08-29 12:02	29	0	0	28	1	Idle
Started	Webhook_Receive	0	2022-08-28 20:04	41	0	0	41	0	Idle

Behind the curtain, a quick glimpse. There are multiple Mirth Channels that are configured as part of the solution. Each channel has a specific purpose, a source and destination. For instance, ACCEPT_ADT_03 channel receives the HL7 ADT message from acute care EHR system, stores the message in raw form and sends back an acknowledgement.

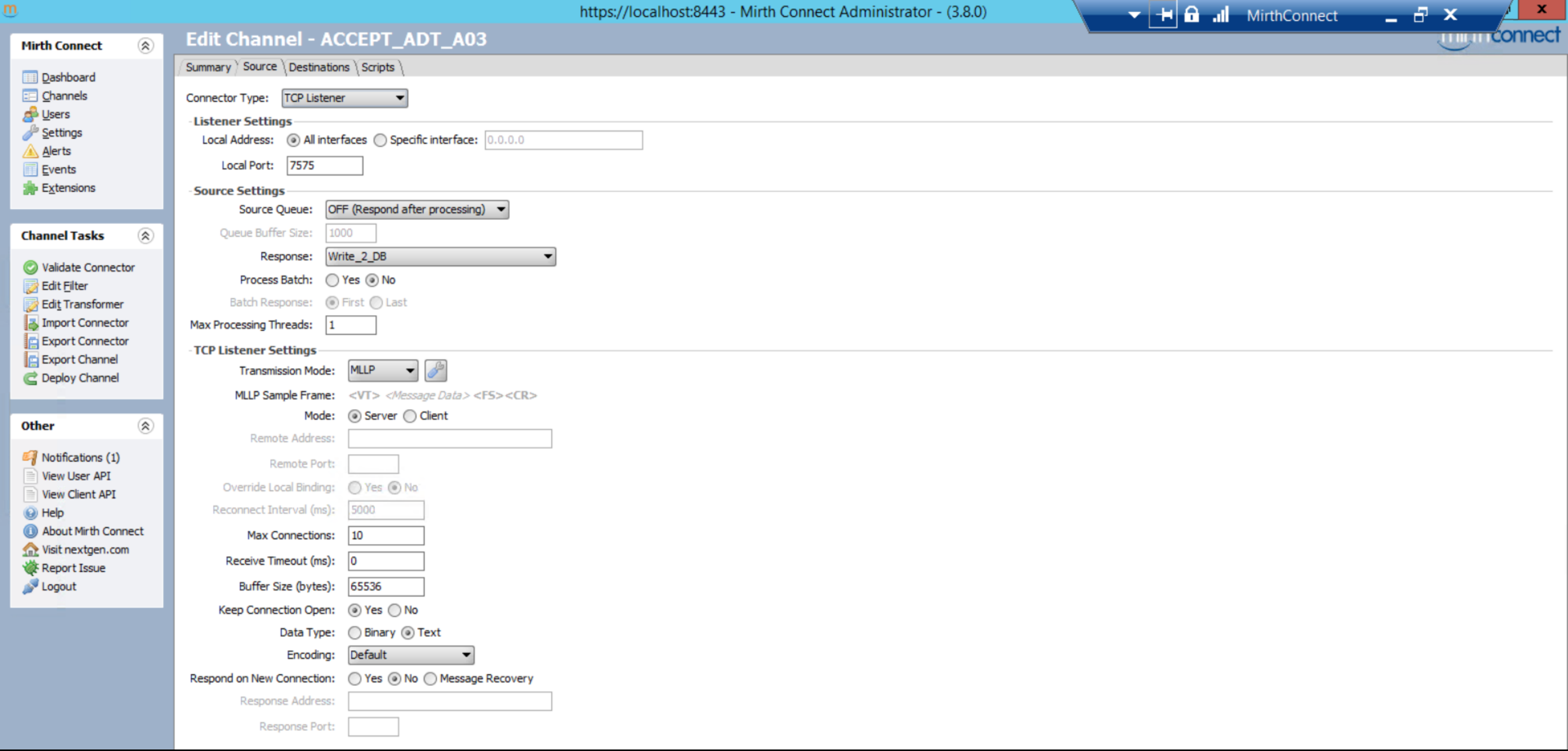

For instance, for a deeper look, the Source definition of ACCEPT_ADT_03 channel listens for messages on a socket and then writes to a database as the destination.

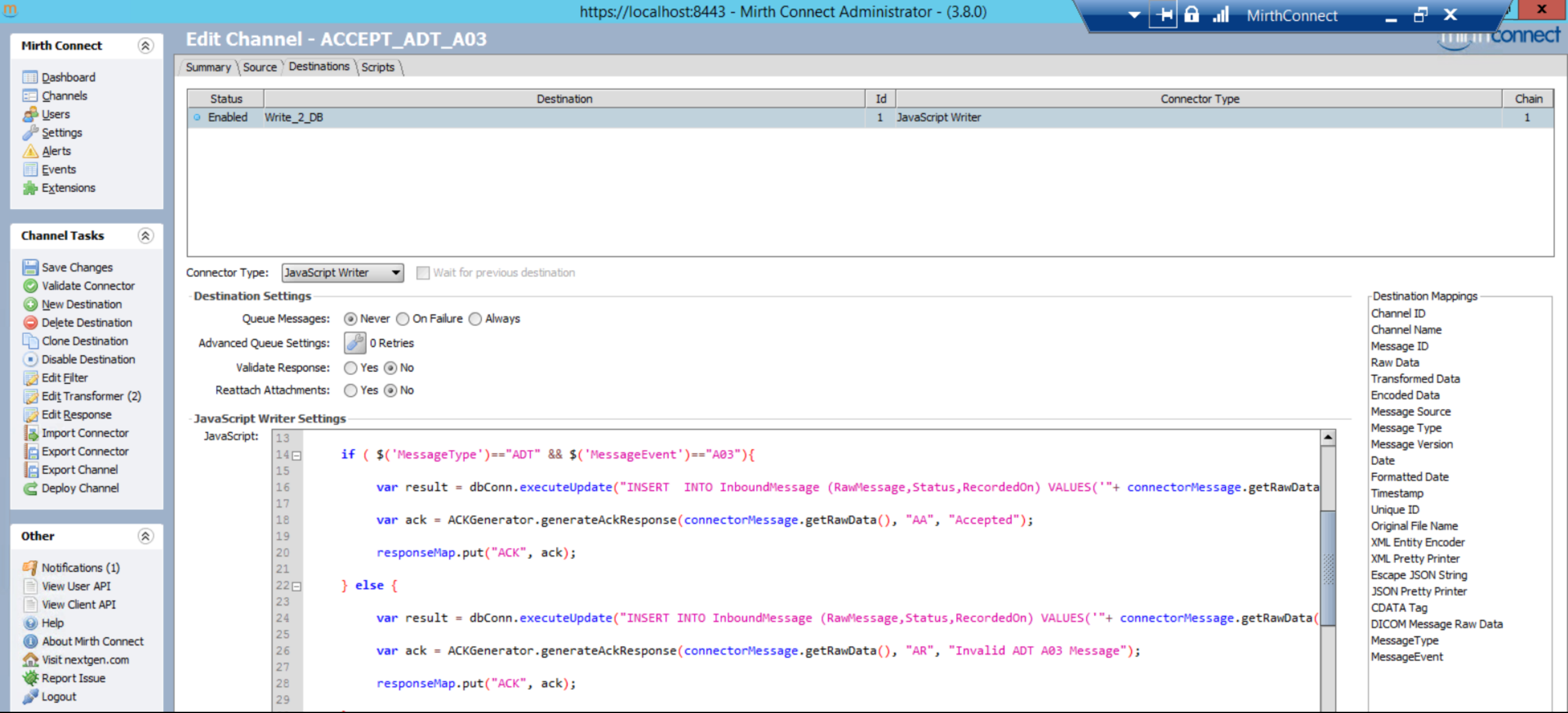

Similarly, here is an example of destination editor. As an example, destination editor for ACCEPT_ADT_03 performs validations and then writes the message to the database in the Destination editor.

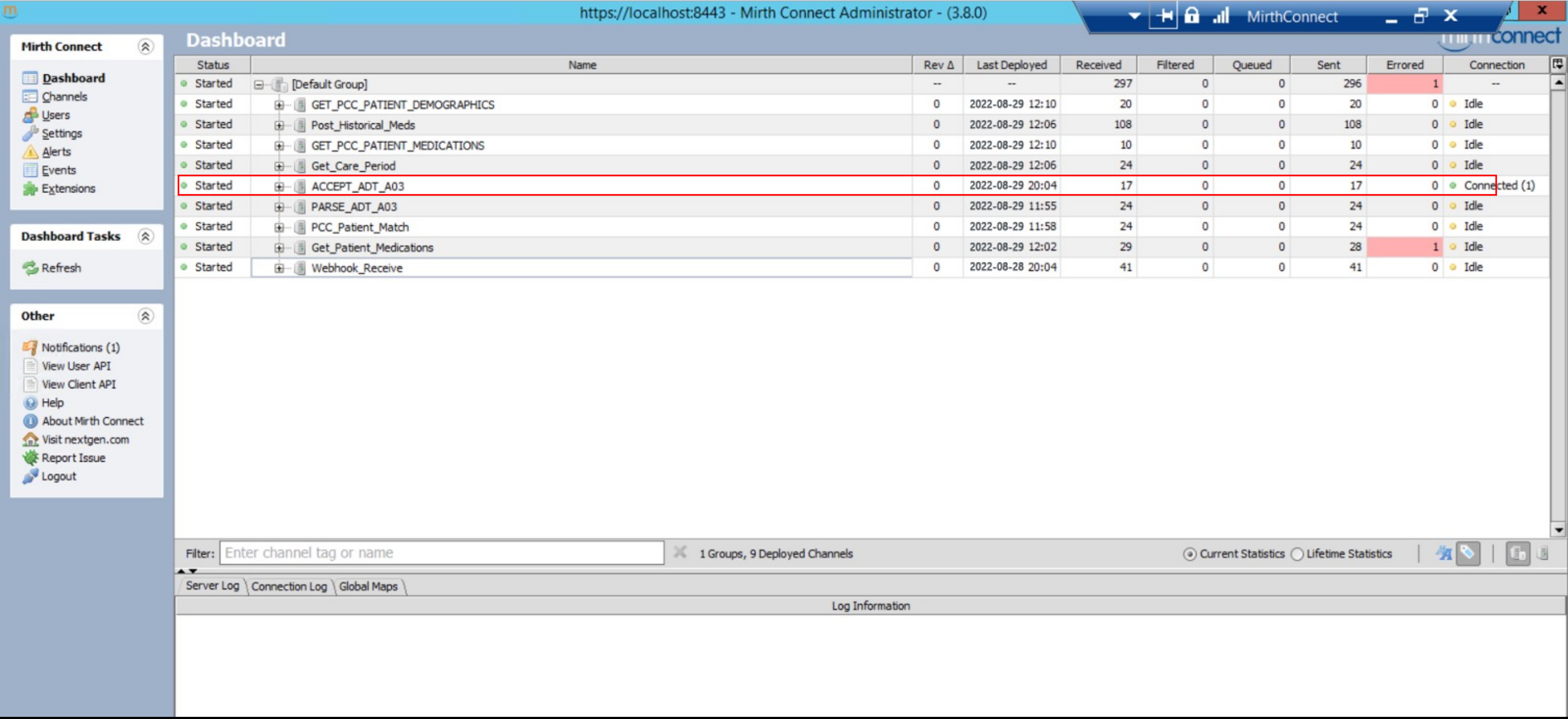

Now some more information on the channels that get to work when the patient is transferred to skilled nursing facility. The ACCEPT_ADT_A03 Mirth Connect channel receives the A03 discharge message from Epic and Sends an Acknowledgement message (ACK)

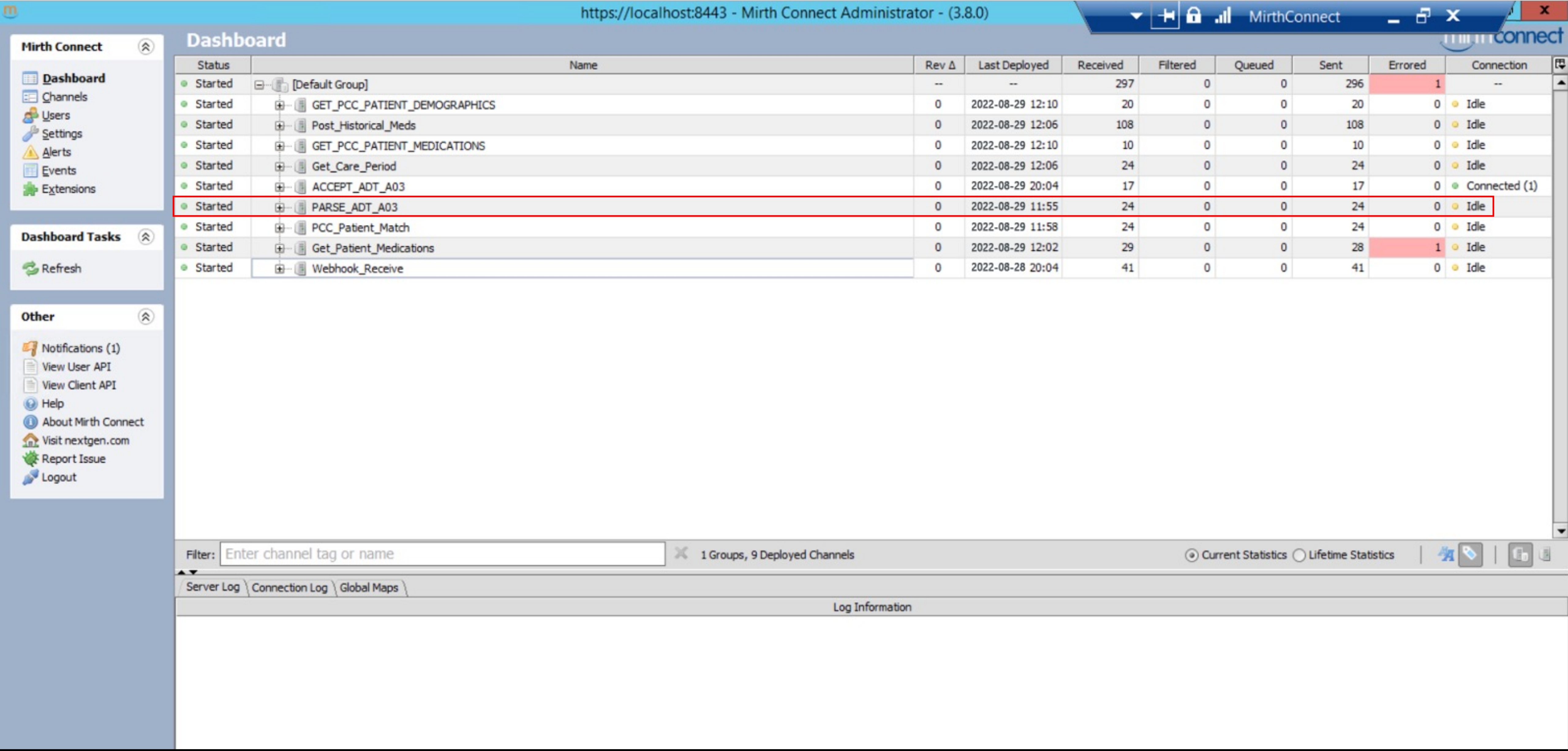

The PARSE_ADT_A03 Mirth Connect channel parses the information from the A03 discharge message and stores the information in the database.

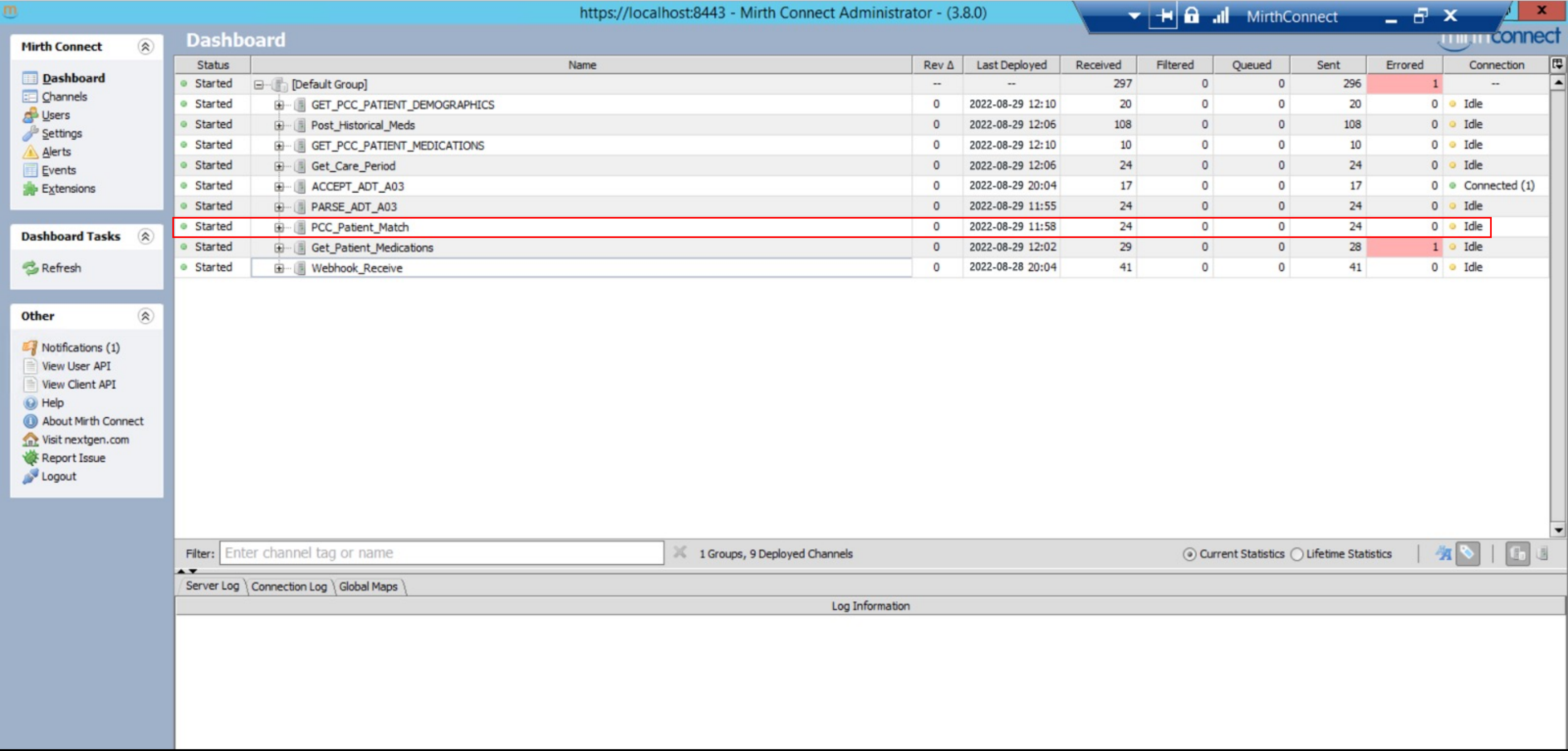

The data stored in the database will then be used by the PCC_Patient_Match mirth channel to call the POST PatientMatch API in Point Click Care.

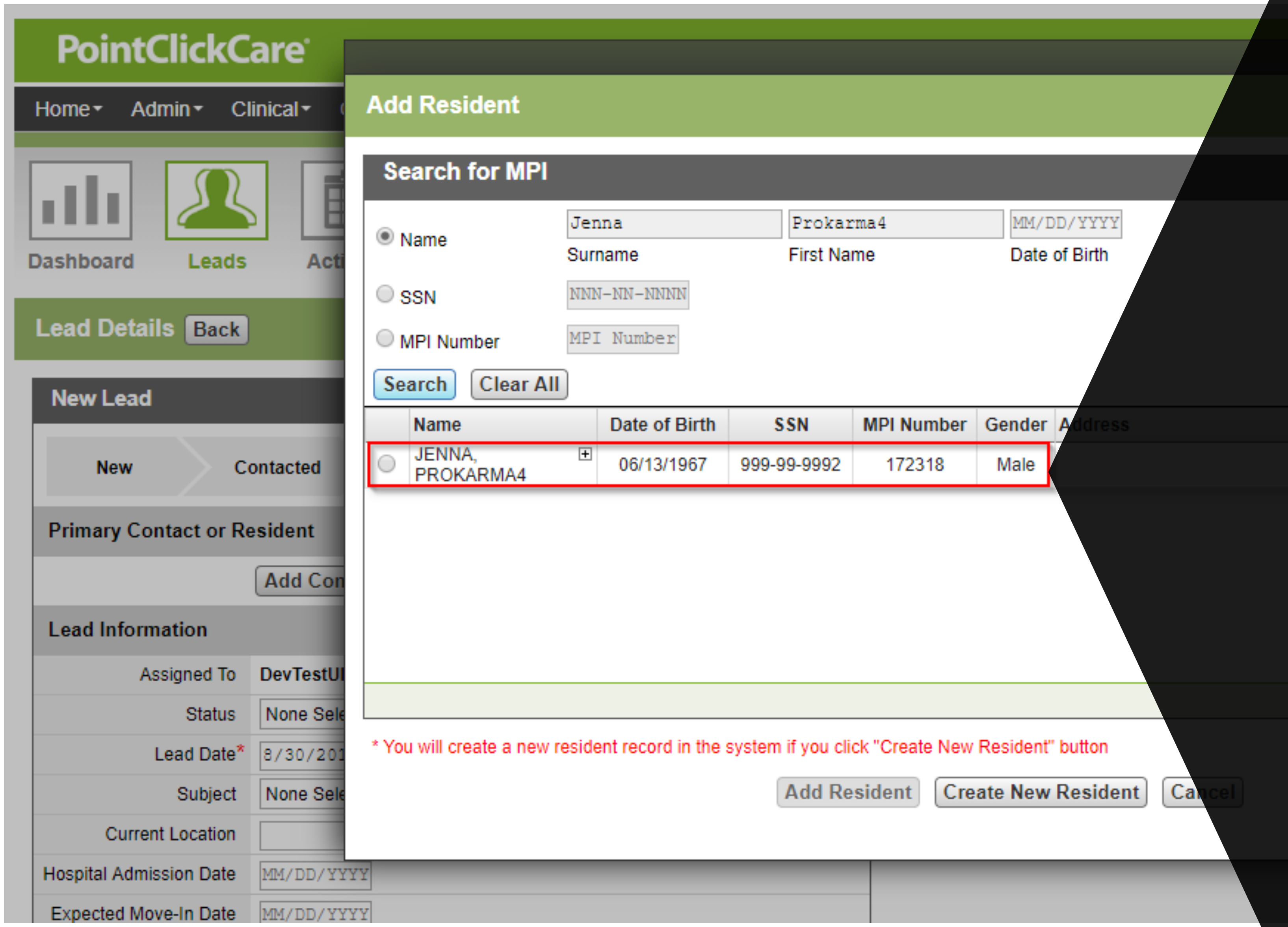

Now let's move onto User Experience in PointClickCare (PCC).
In PCC , the patient can be searched, selected, and waitlisted or admitted to a facility. End user workflow for a PCC user is identical, regardless of whether patient is new or existing.

In case of new patient, behind the scenes, a webhook message automatically notifies Mirth Connect with a patient identifier. For an existing patient, Mirth Connect will already have a Patient ID.

Mirth Connect then uses a FHIR query to gather the patient's medications from EPIC and sends the medication list to PCC

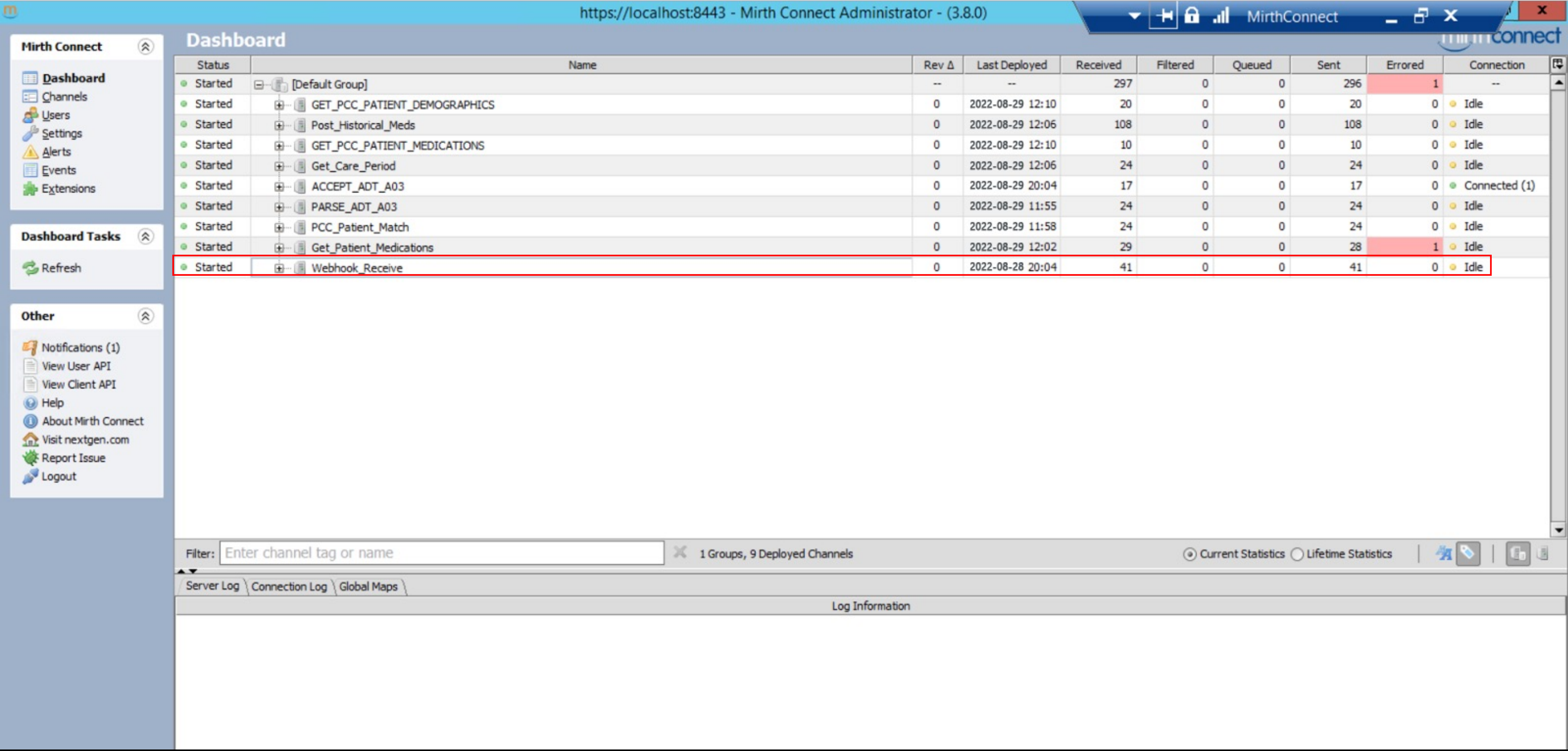

Once the patient has an ID assigned in PointClickCare, Mirth Connect will receive a webhook message on the Webhook_Recieve channel.

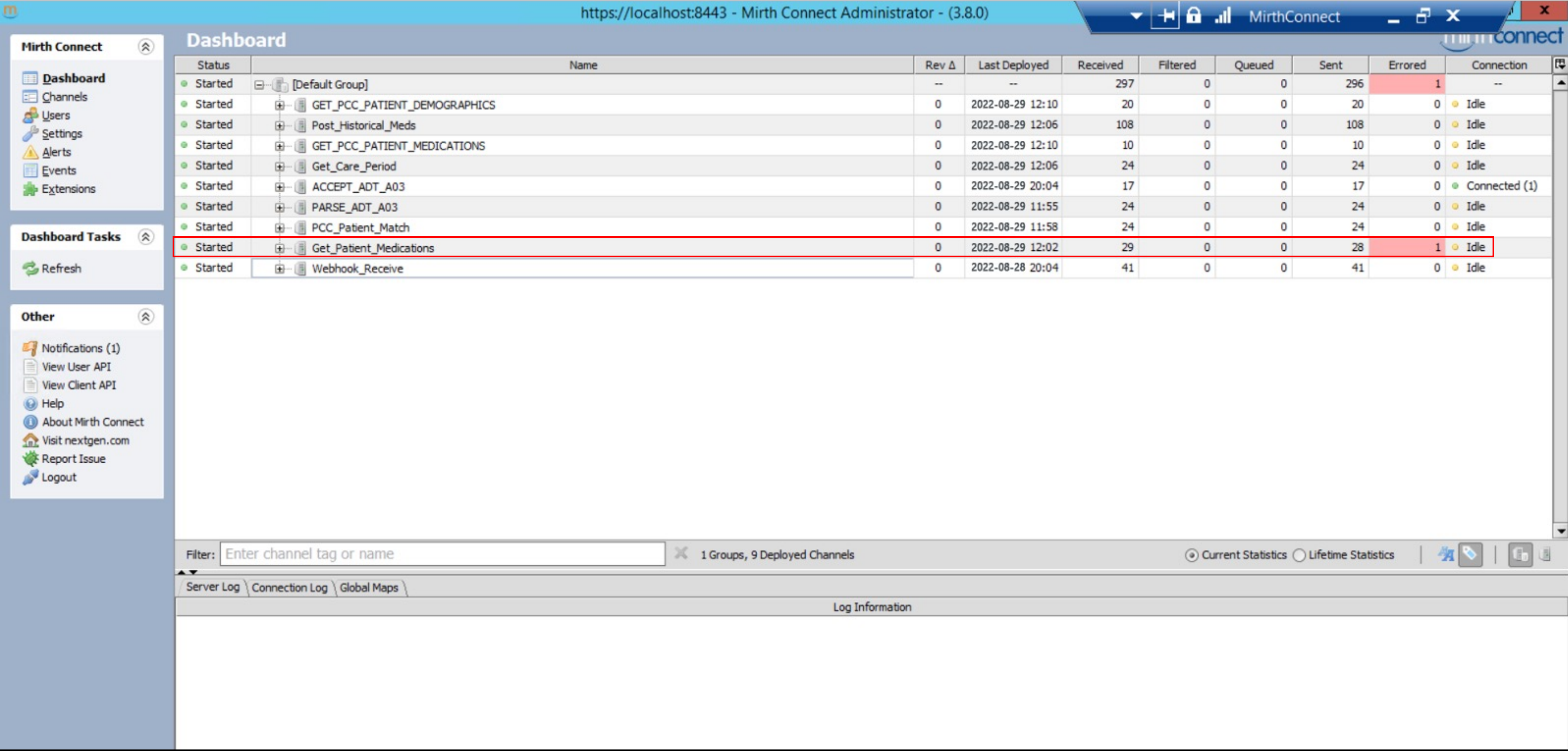

The Get_Patient_Medication channel will then create FHIR queries to gather the patient's FHIR ID and the patient's medications.

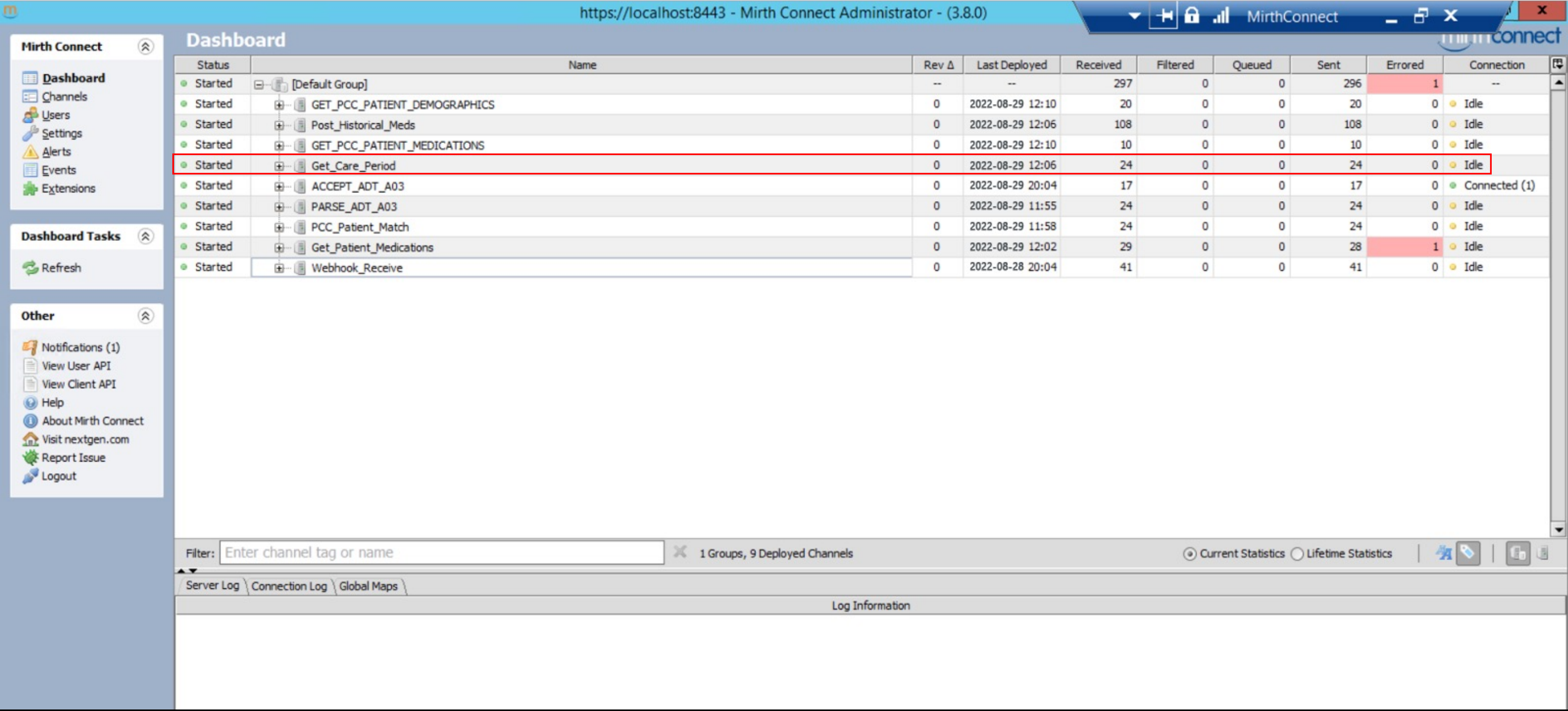

Once the medication information is gathered, Mirth Connect will use the GET_Care_Period to collect the admit and discharge dates from the stored A03 data. A care period is constructed with this information and pushed to PointClickCare through the POST CarePeriod API.

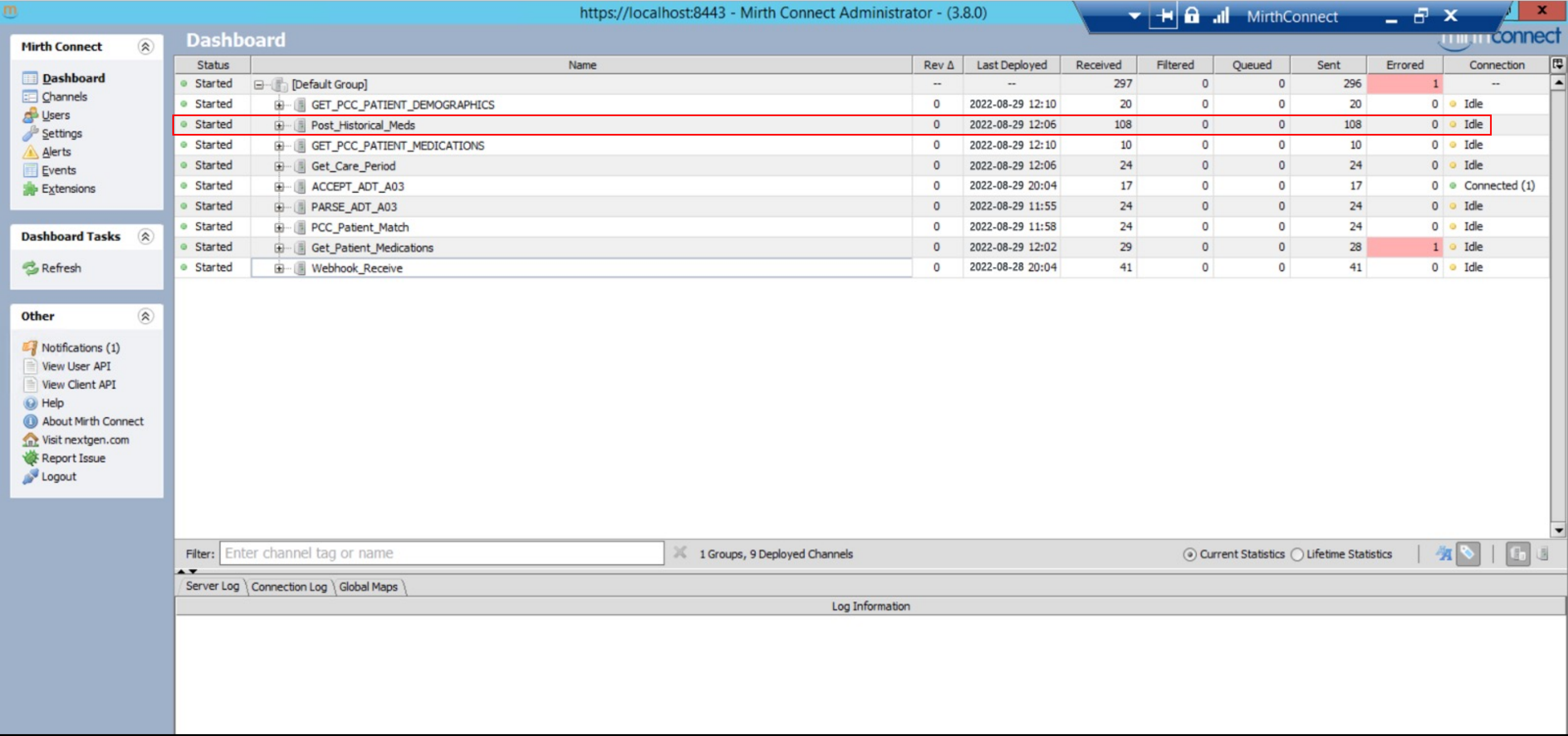

Once the care period is created, Mirth Connect will use the Post_Historical_Medications to push the medications to PointClickCare through a POST HistoricalMedications API call.

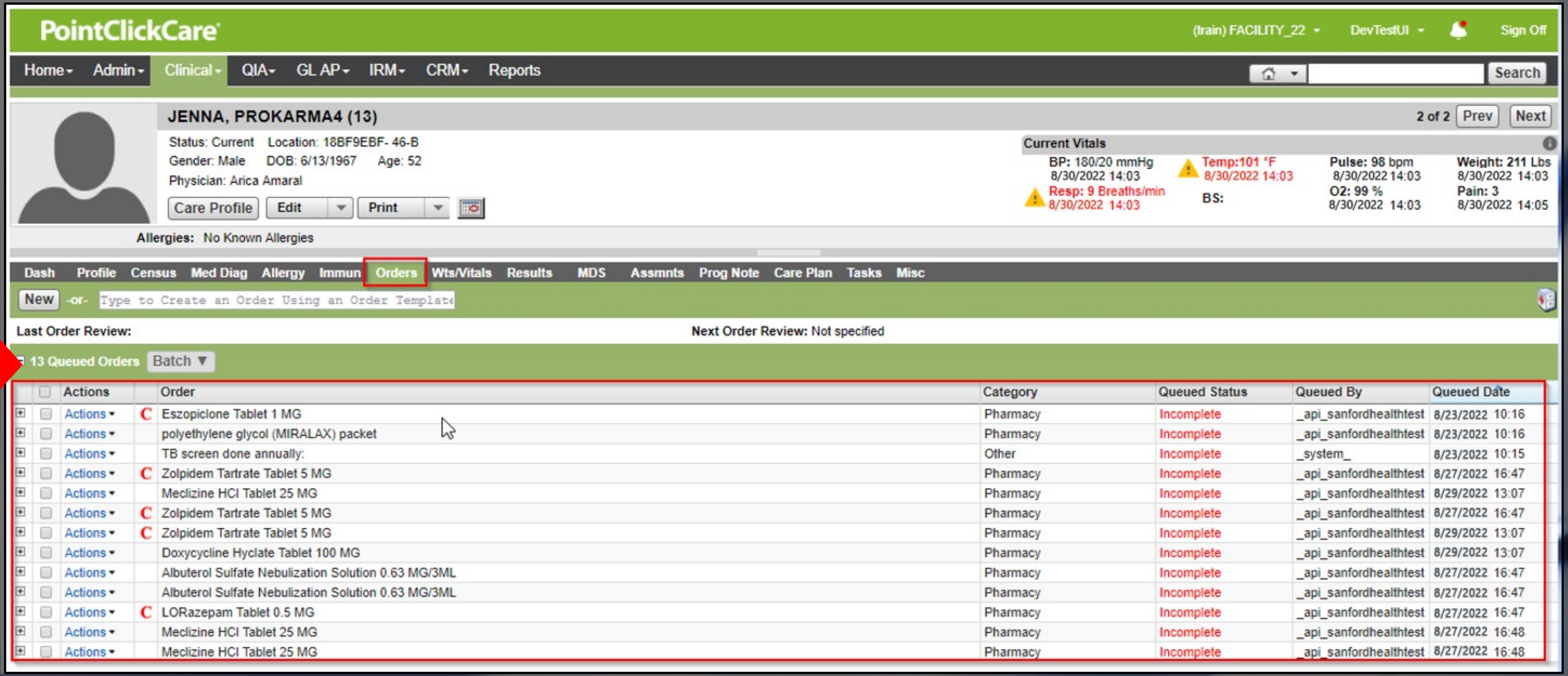

The medications gathered from Epic are then pushed into PCC (when the patient is found after the initial search) . They will show up under the orders tab as "queued orders"

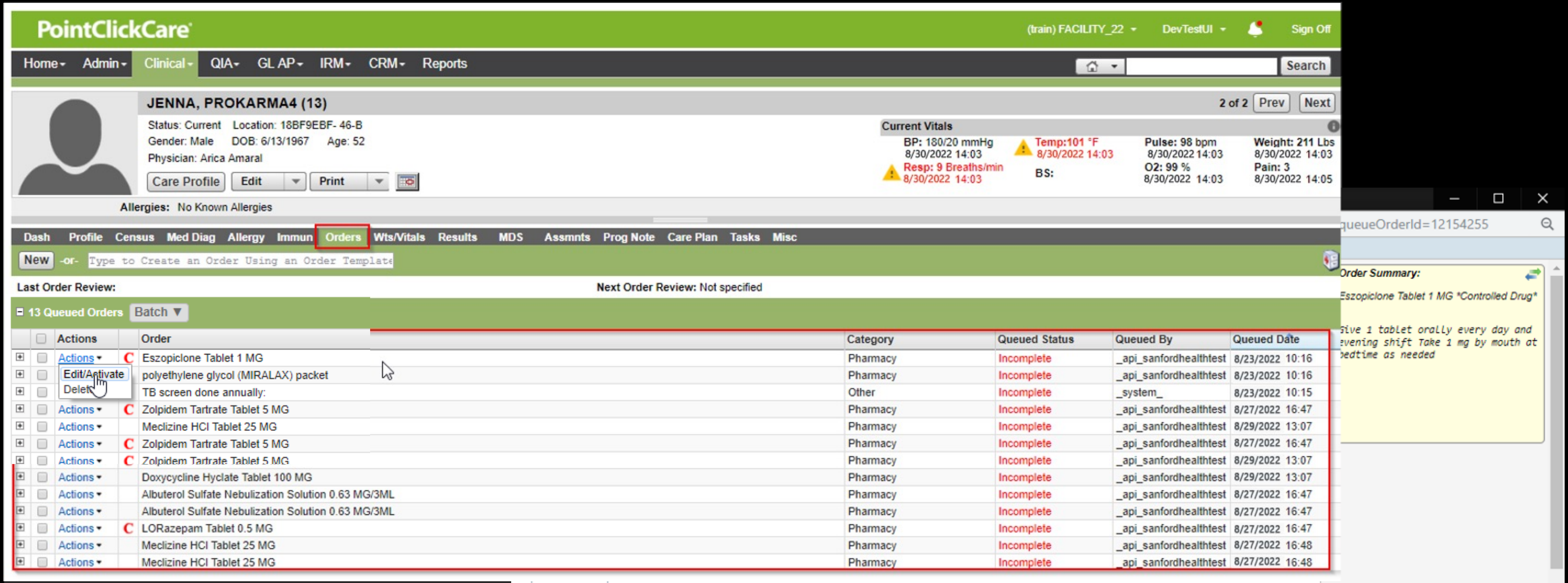

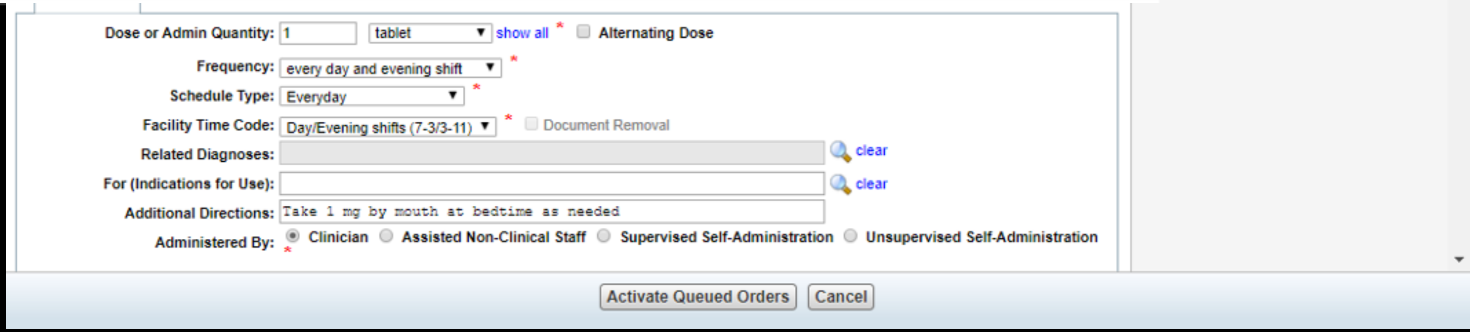

The medications can then be edited and activated, and added to the patient's chart.

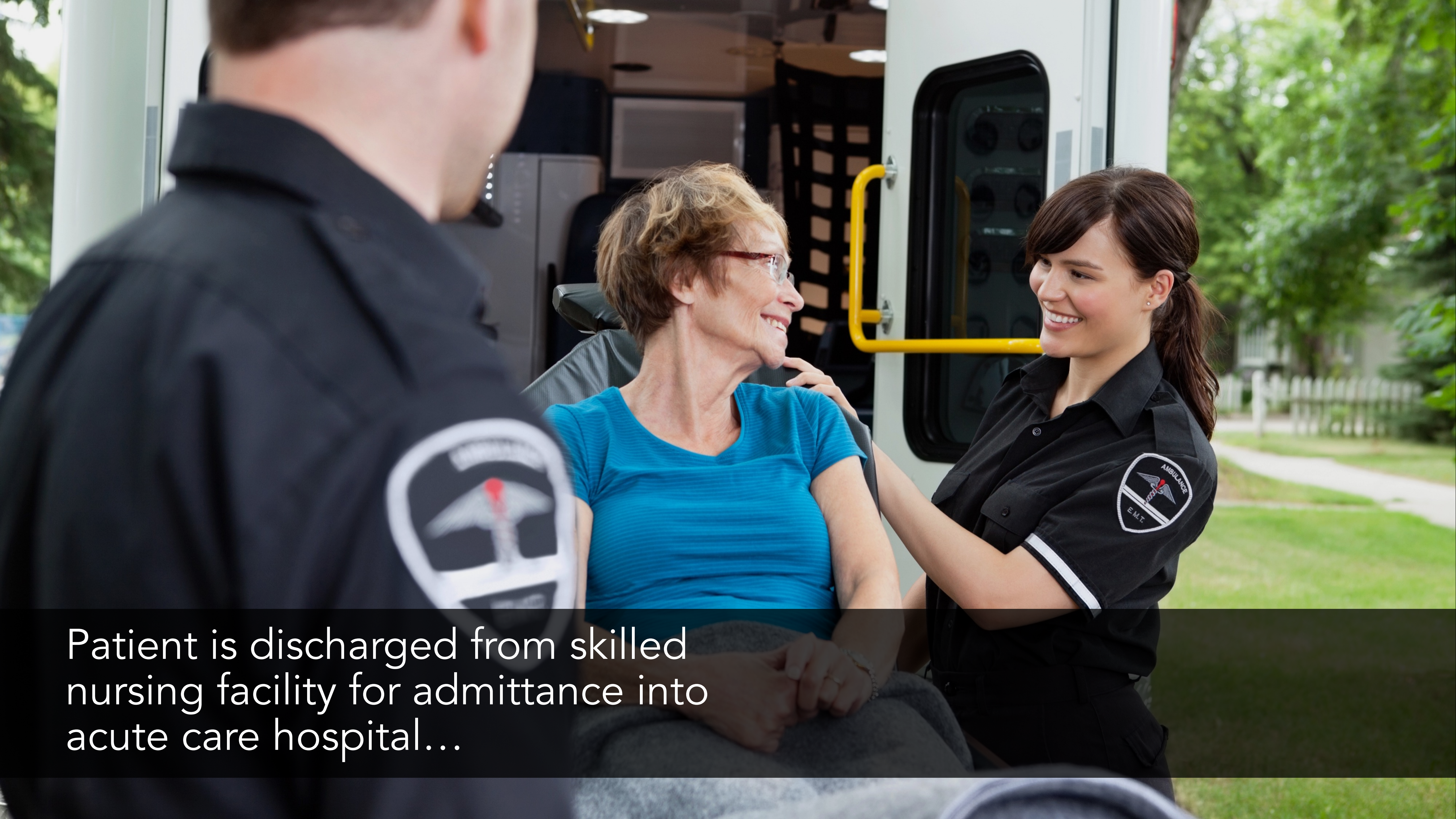
Patient is discharged from skilled nursing facility for admittance into acute care hospital…

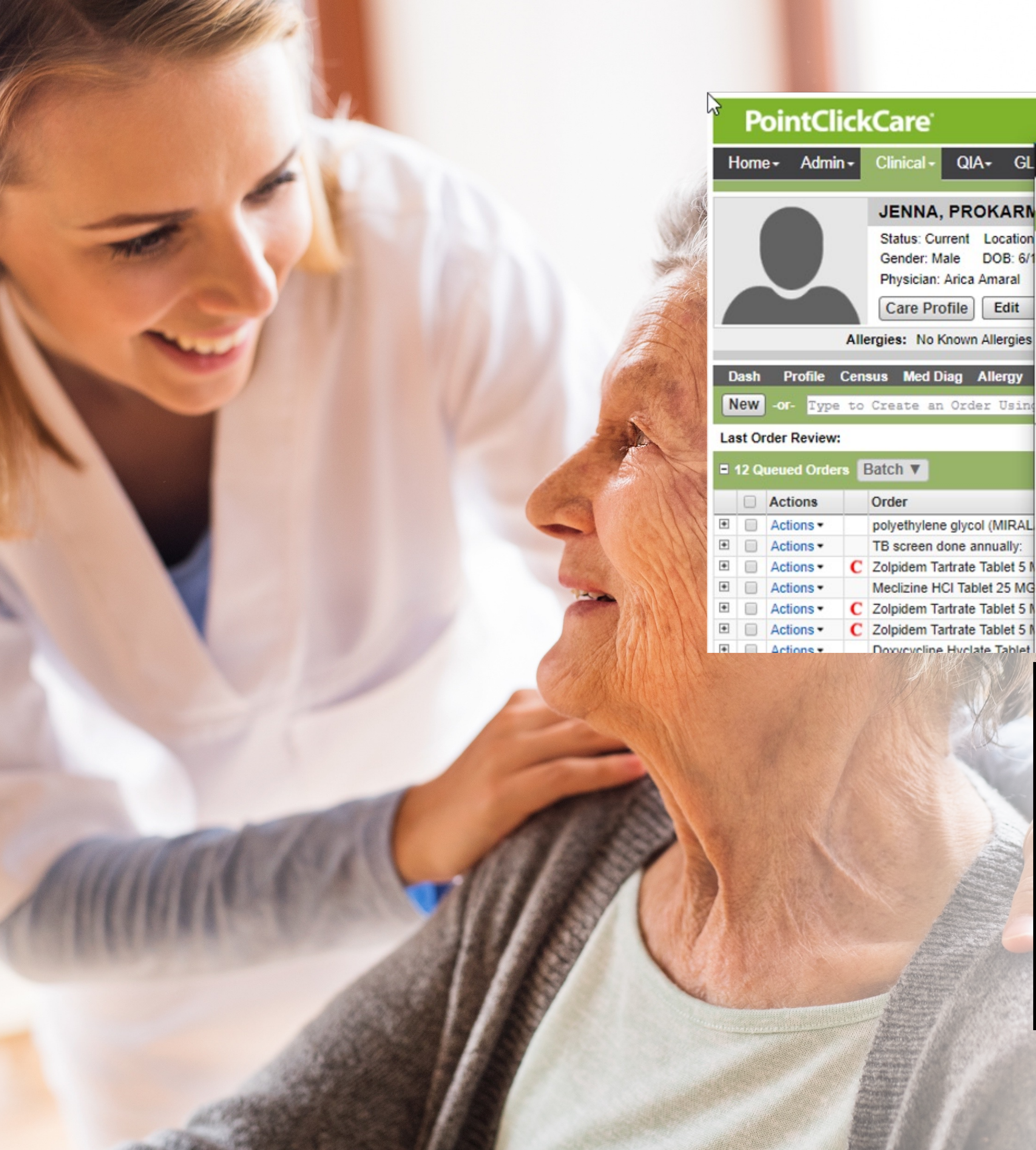

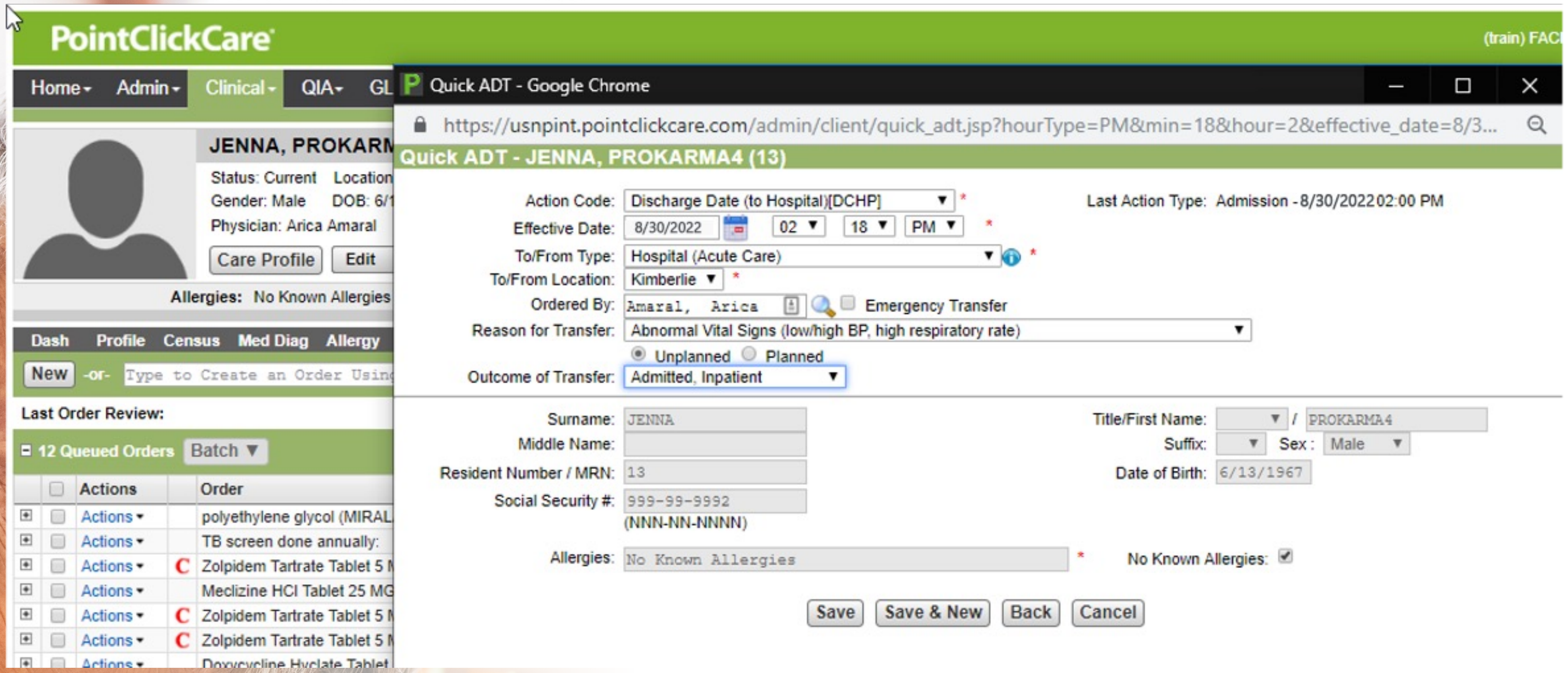

In the other direction, if a patient is discharged from a SNF to the acute care hospital a webhook message will notify Mirth Connect of the discharge.

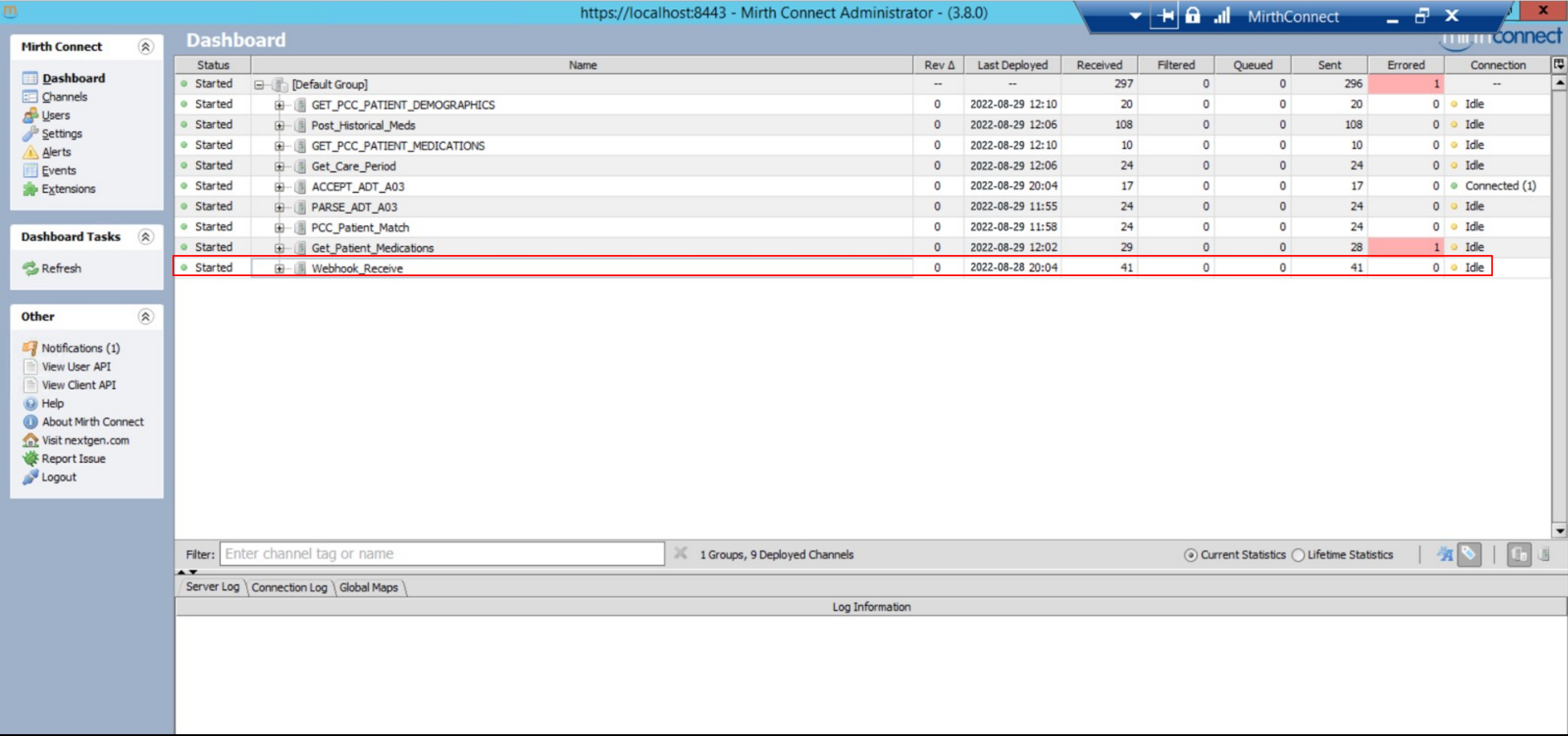

Upon discharge, Mirth connect receives the discharge notification on the Webhook_Recieve channel.

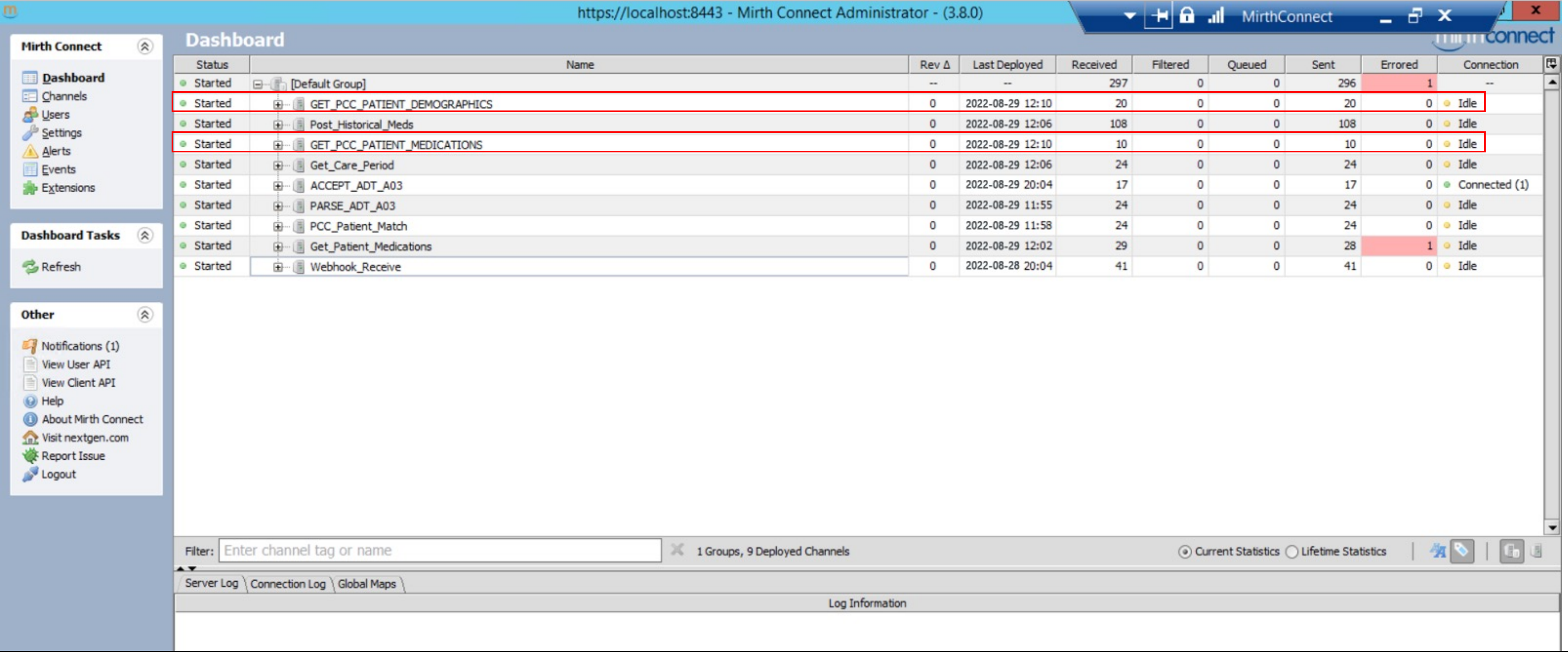

Mirth connect will then use the GET_PCC_PATIENT_MEDICATIONS and GET_PCC_PATIENT_DEMOGRAPHICS to gather the patient’s medication list and demographics using the GET PatientMedications and Get Patient APIs from Point Click Care. This information will then be used to construct the CCD for the patient.

```
<?xml version="1.0" encoding="UTF-8"?>
- <ClinicalDocument xsi:schemaLocation="urn:hl7-org:v3 CDA.xsd" xmlns="urn:hl7-org:v3" xmlns:xsi="http://www.w3.org/2001/XMLSchema-instance">
    <realmCode code="US"/>
    <typeId extension="POCD_HD000040" root="2.16.840.1.113883.1.3"/>
    <templateId root="2.16.840.1.113883.10.20.22.1.2"/>
    <templateId root="2.16.840.1.113883.10.20.22.1.1"/>
    <id extension="PCC" root="1.19.840.1.113883.19.4"/>
    <code code="34133-9" displayName="Summarization of Episode Note" codeSystemName="LOINC" codeSystem="2.16.840.1.113883.6.1"/>
    <title>Clinical Summary</title>
    <effectiveTime value="20190820141005"/>
    <confidentialityCode code="N" displayName="Normal" codeSystemName="Normal" codeSystem="2.16.840.1.113883.5.25"/>
    <languageCode code="en-US"/>
  + <recordTarget>
  + <author>
  + <custodian>
  + <documentationOf>
  - <component>
    - <structuredBody>
      - <component>
        - <section>
            <templateId root="2.16.840.1.113883.10.20.22.2.1.1"/>
            <templateId root="2.16.840.1.113883.10.20.22.2.1"/>
            <id extension="PCC" root="9505E912-B21C-11E9-9B65-5804E5EE60C2"/>
            <code code="10160-0" displayName="History of medication use" codeSystemName="LOINC" codeSystem="2.16.840.1.113883.6.1"/>
            <title>Medications</title>
          + <text>
          - <entry>
            - <substanceAdministration classCode="SBADM" moodCode="EVN">
                <templateId root="2.16.840.1.113883.10.20.22.4.16"/>
                <id extension="PCC" root="9505E912-B21C-11E9-9B65-5804E5E650C2"/>
                <text>Aspirin 81 MG Oral Tablet</text>
                <statusCode code="ACTIVE"/>
              - <effectiveTime xsi:type="IVL_TS">
                  <low value="20151024"/>
                  <high value=""/>
                </effectiveTime>
              - <routeCode code="C38288" displayName="ORAL" codeSystemName="NCI Thesaurus" codeSystem="2.16.840.1.113883.3.88.12.3221.8.7">
                  <originalText>ORAL</originalText>
                </routeCode>
                <approachSiteCode codeSystemName="SNOMEDCT" codeSystem="2.16.840.1.113883.6.96"/>
                <doseQuantity value="81.0" unit="MG"/>
              - <consumable>
                - <manufacturedProduct classCode="MANU">
                    <templateId root="2.16.840.1.113883.10.20.22.4.23"/>
                  - <manufacturedMaterial>
                      <code code="243670" displayName="Aspirin 81 MG Oral Tablet" codeSystemName="RxNorm" codeSystem="2.16.840.1.113883.6.88"/>
                    </manufacturedMaterial>
                  </manufacturedProduct>
                </consumable>
              - <entryRelationship typeCode="SUBJ">
                - <observation classCode="OBS" moodCode="EVN">
                    <templateId root="2.16.840.1.113883.10.20.1.18"/>
                    <code code="31" displayName="one time a day" codeSystemName="PCC" codeSystem="1.2.8.114350.1.13.138.3.7.2.697081"/>
                    <statusCode code="completed"/>
                    <value xsi:type="ST">one time a day</value>
                  </observation>
                </entryRelationship>
```


Sample CCD (Continuity of Care Document) that is generated for ingestion into Epic

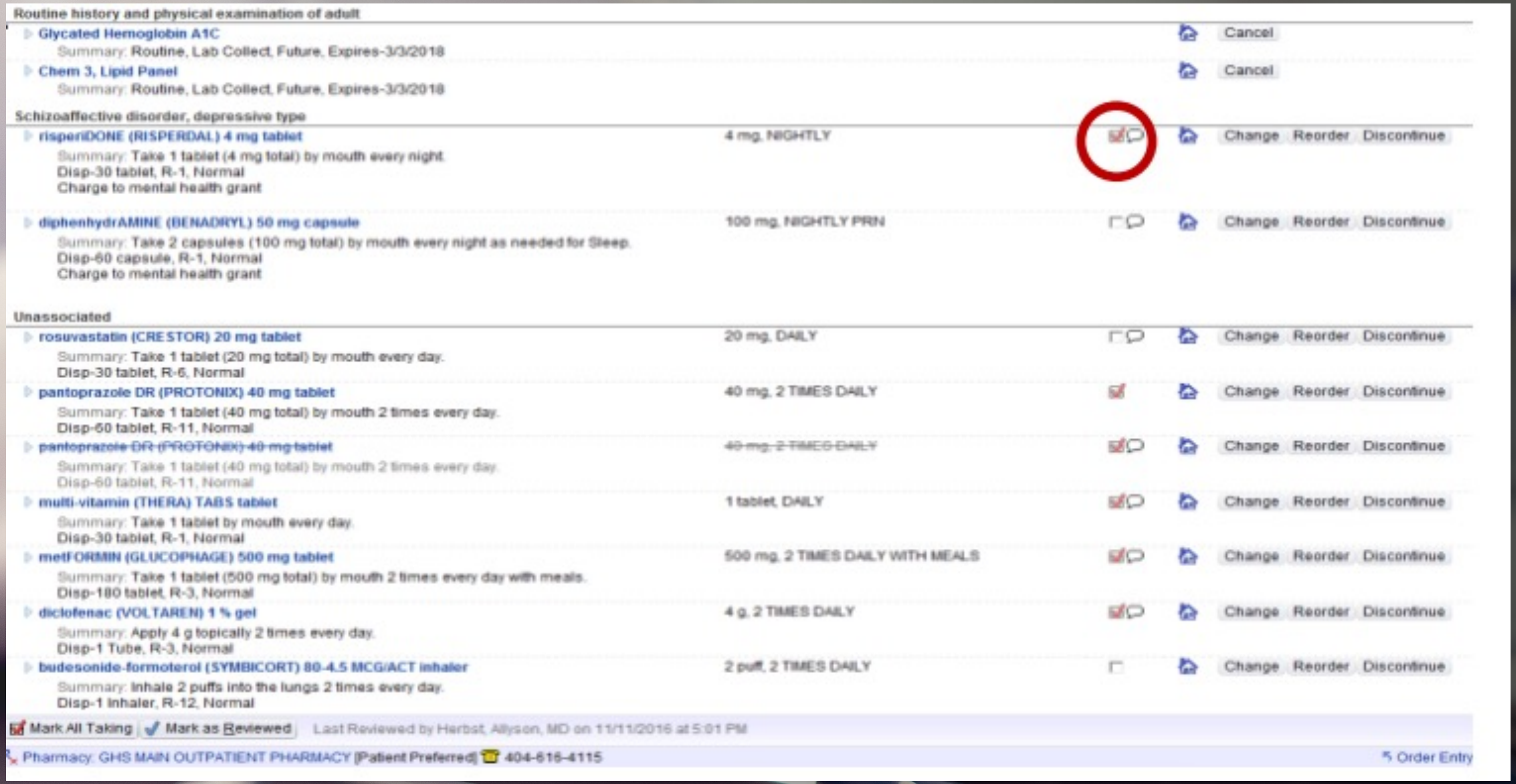

Mirth Connect will then push the CCD document to Epic. The medications will then be viewable through the Care Everywhere application and can be reconciled to the patient's chart

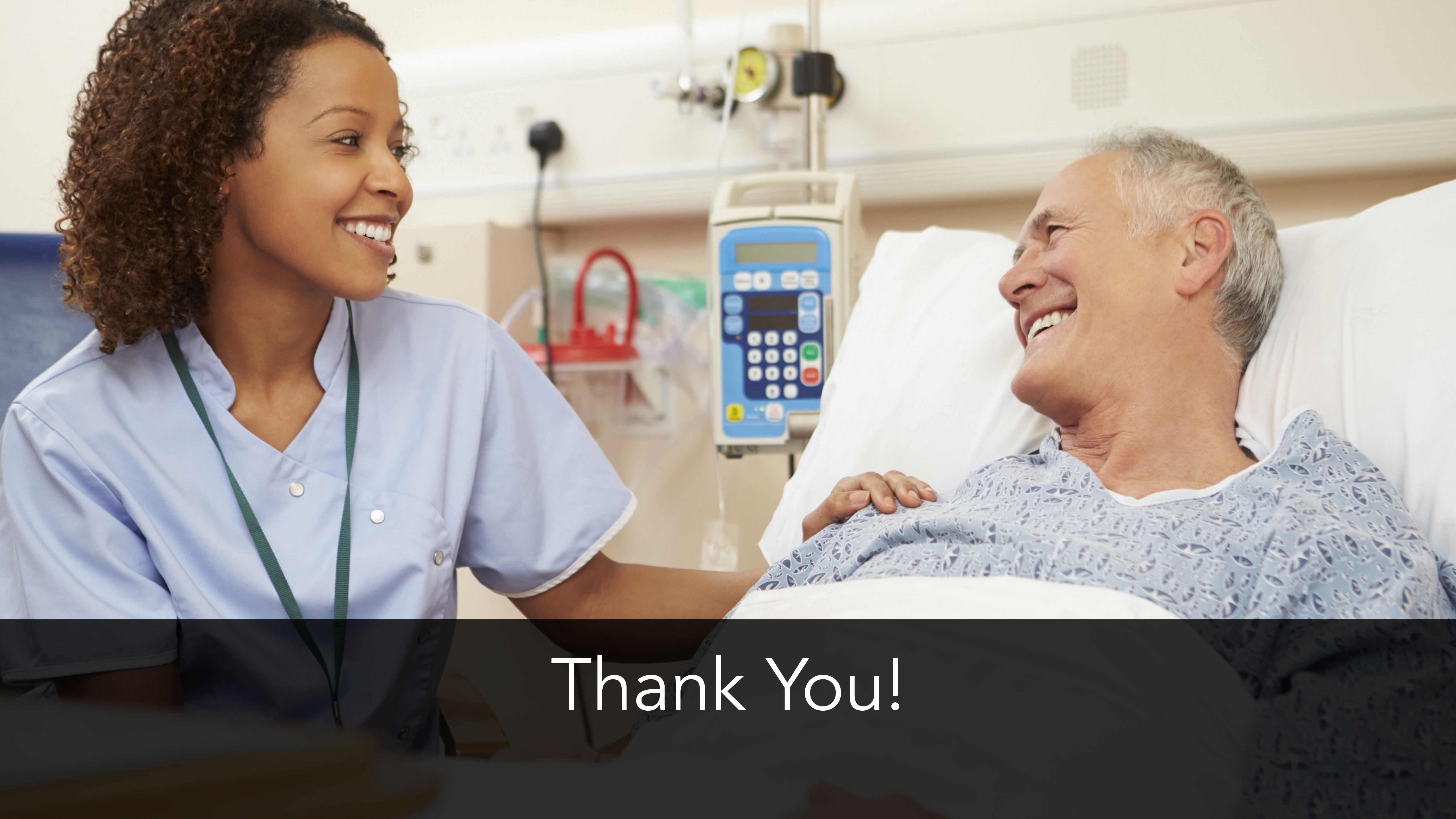
Thank You!
