# Supplementary material for: Design, Development, and Evaluation of an Automated Solution for Electronic Information Exchange Between Acute and Long-term Postacute Care Facilities: Design Science Research
Source: JMIR Form Res. 2023 Feb 17;7:e43758. doi: 10.2196/43758 (PMC9985001; doi:10.2196/43758)
Supplement: Multimedia Appendix 5 [file formative_v7i1e43758_app5.pdf]

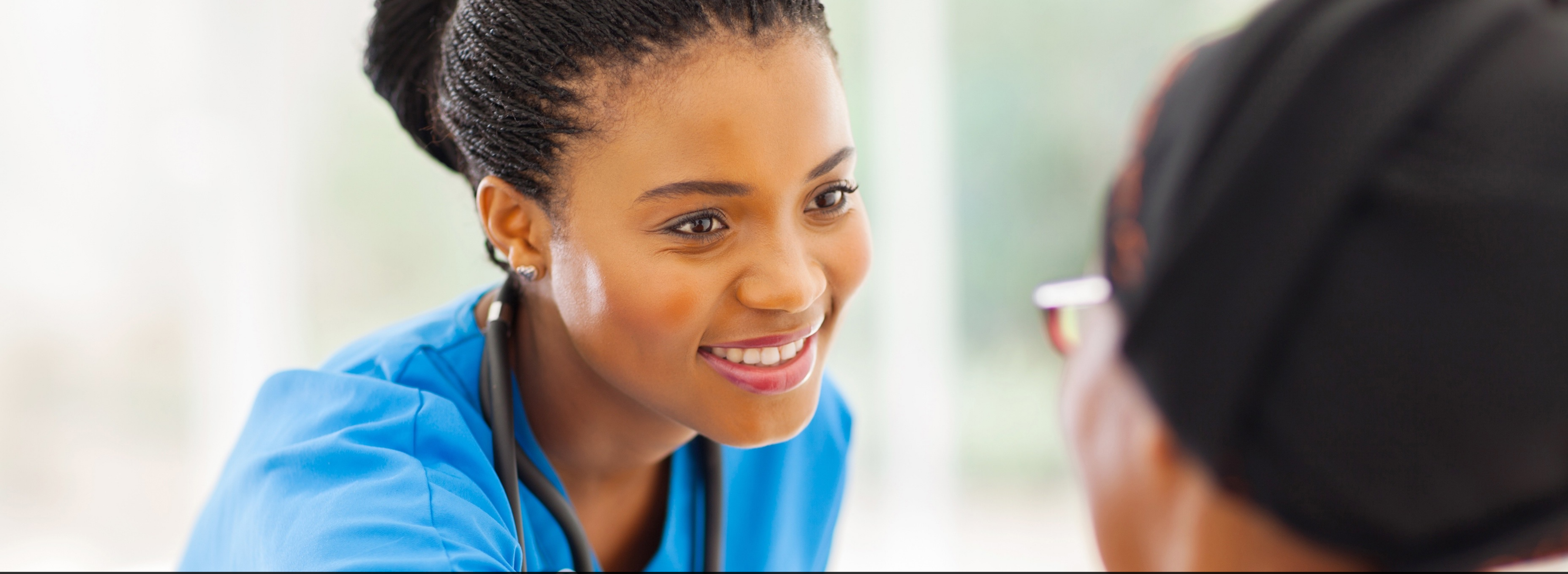

Epic Discharge Workflow

A patient at a acute care hospital is discharged to a skilled nursing home.

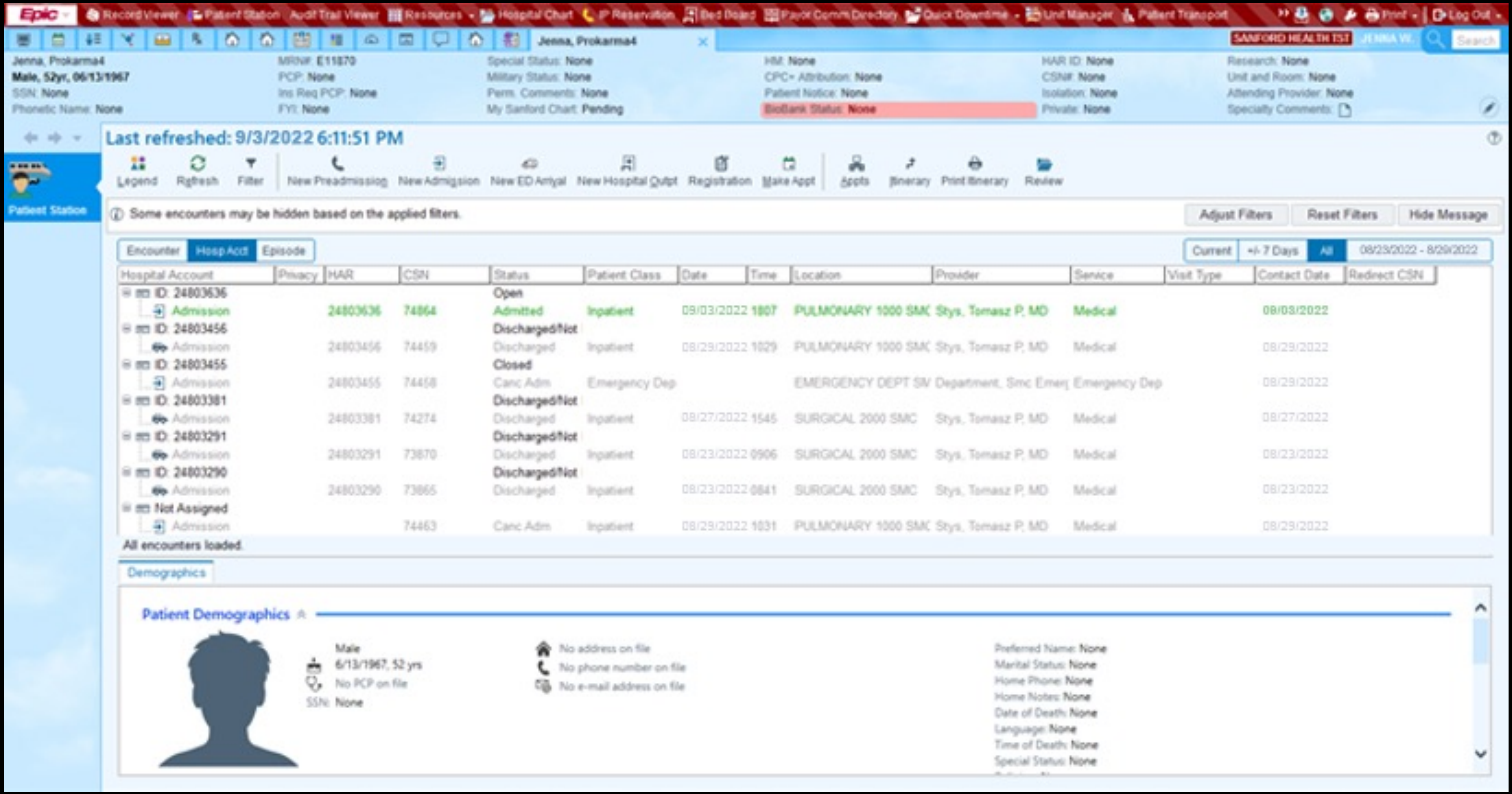

Patient is in Acute Care Facility

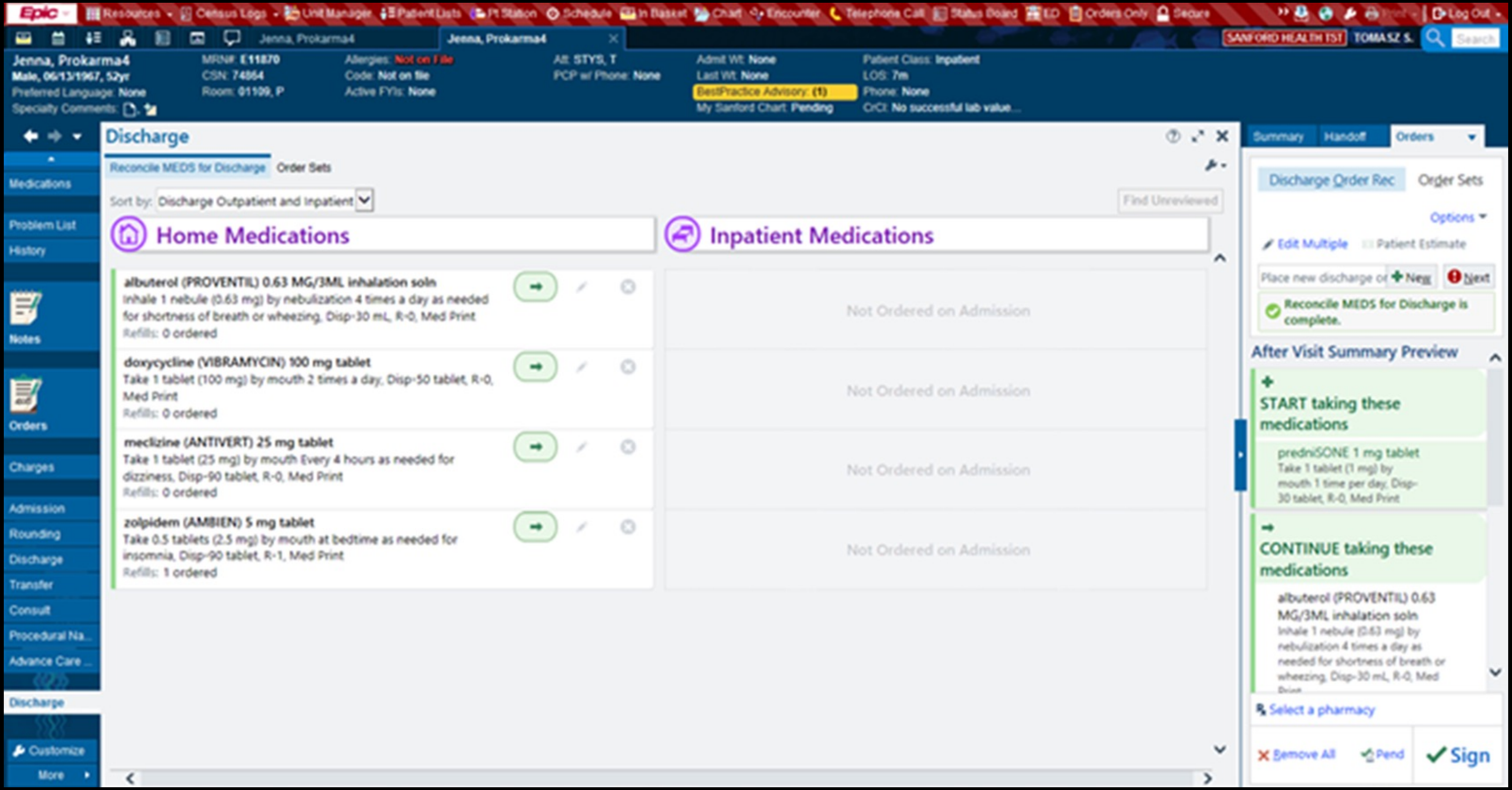

Before patient is discharged, continued all home meds, added a new discharge order for Prednisone, 1 mg medication

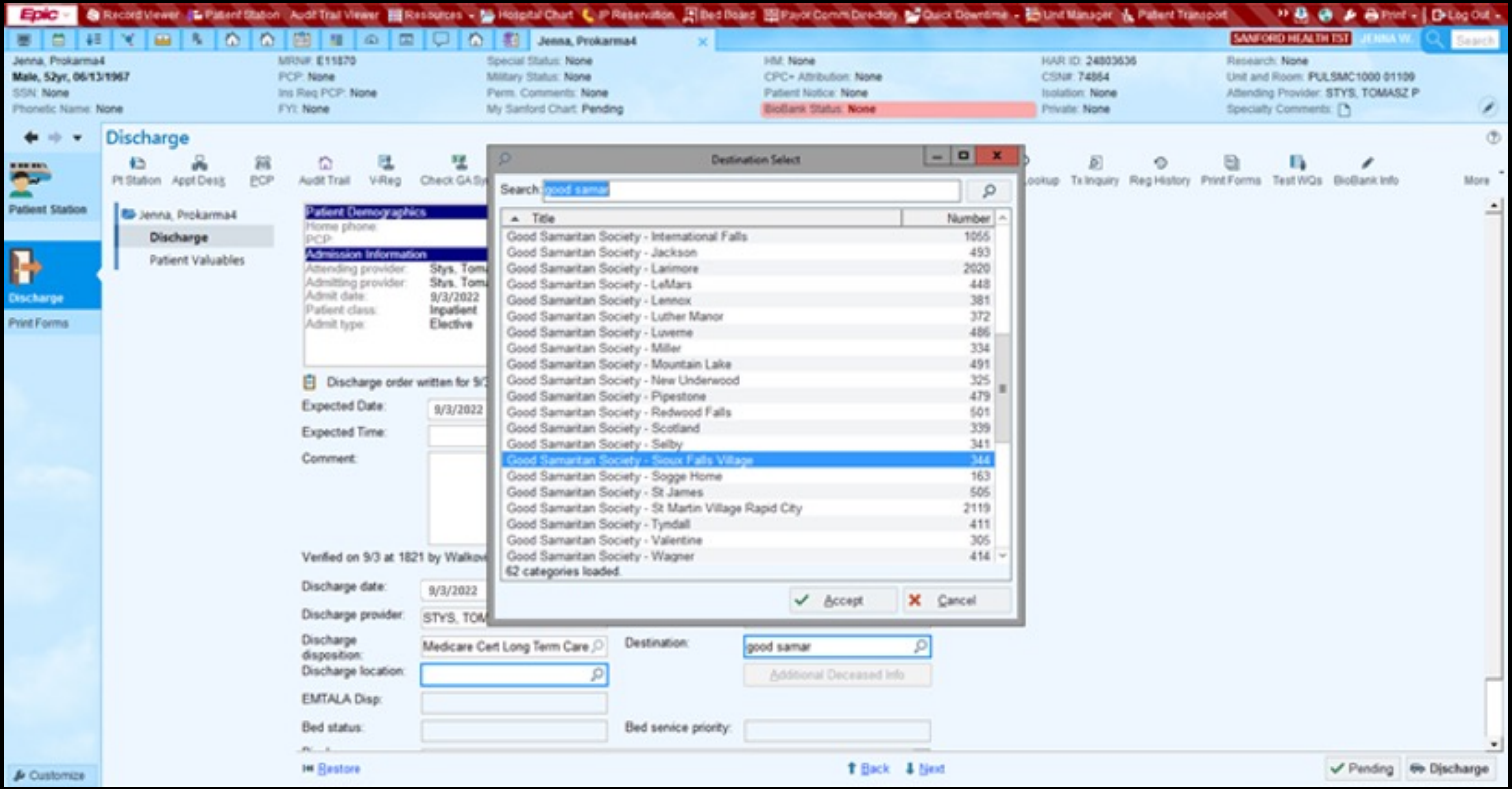

Discharge date, time, disposition, and destination are entered.

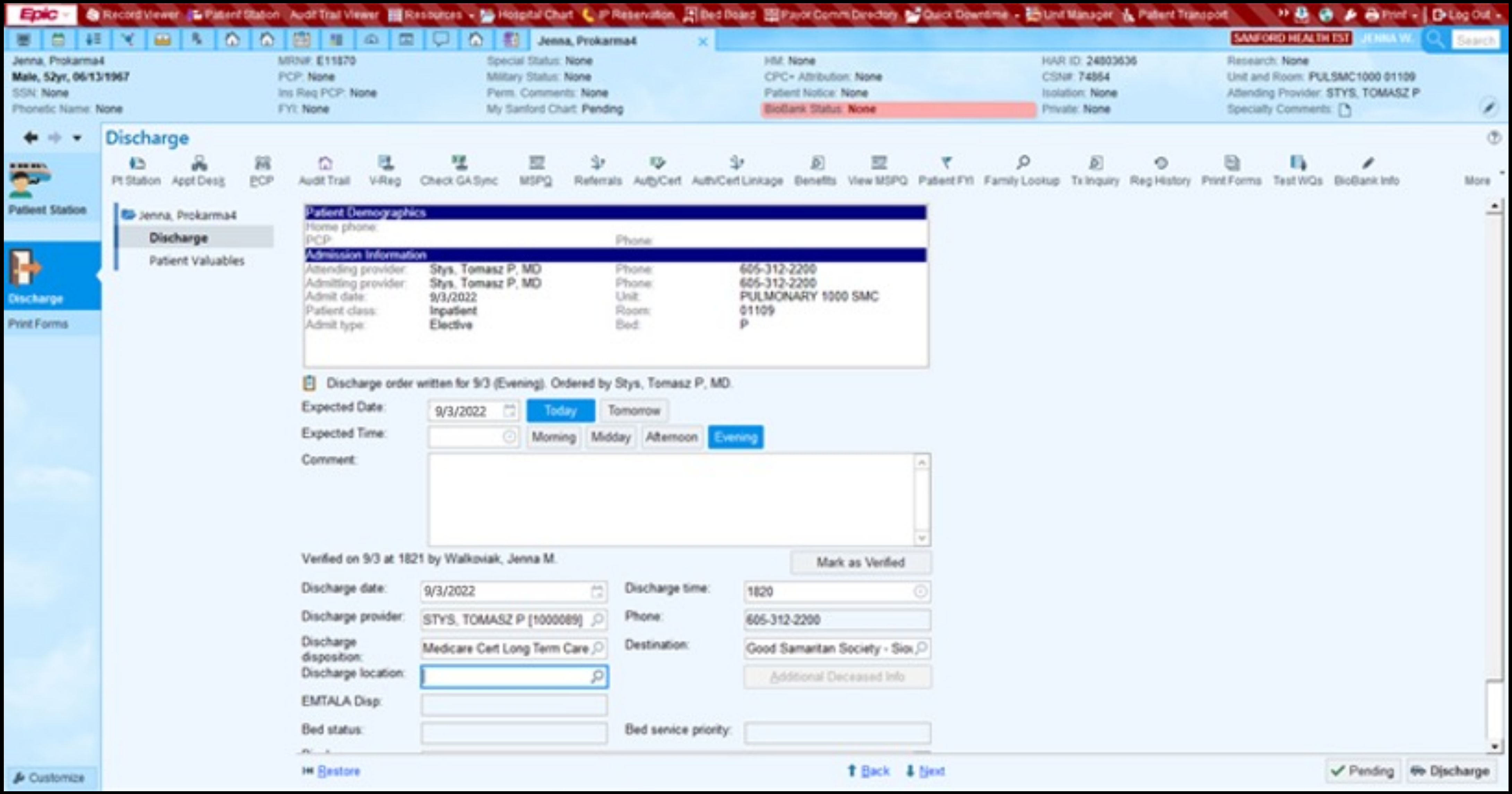

Patient has been discharged from acute care facility and is enroute to skilled nursing facility

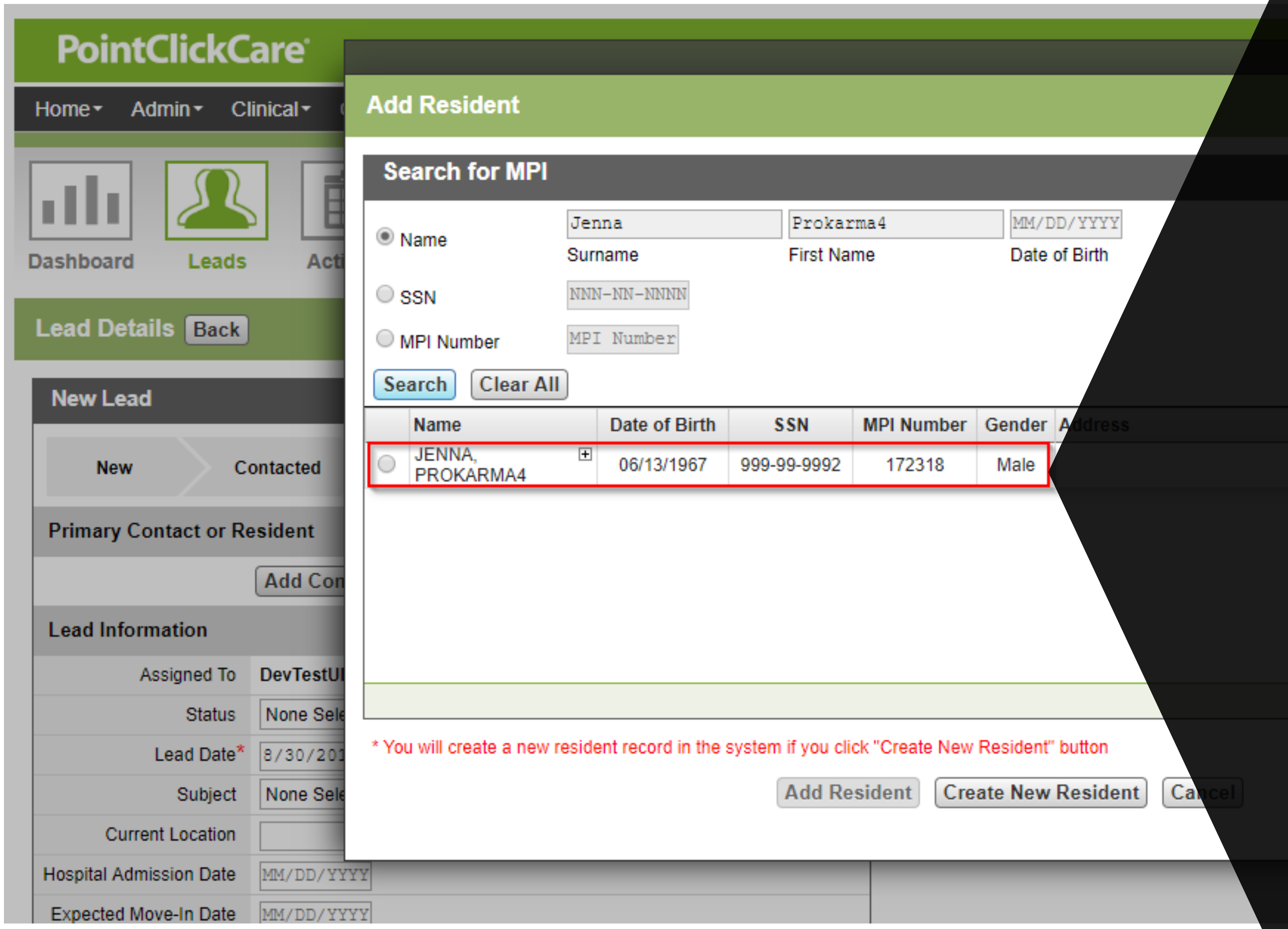

Now let's move onto User Experience in PointClickCare (PCC).
In PCC , the patient can be searched, selected, and waitlisted or admitted to a facility. End user workflow for a PCC user is identical, regardless of whether patient is new or existing.

In case of new patient, behind the scenes, a webhook message automatically notifies Mirth Connect with a patient identifier. For an existing patient, Mirth Connect will already have a Patient ID.

Mirth Connect then uses a FHIR query to gather the patient's medications from EPIC and sends the medication list to PCC

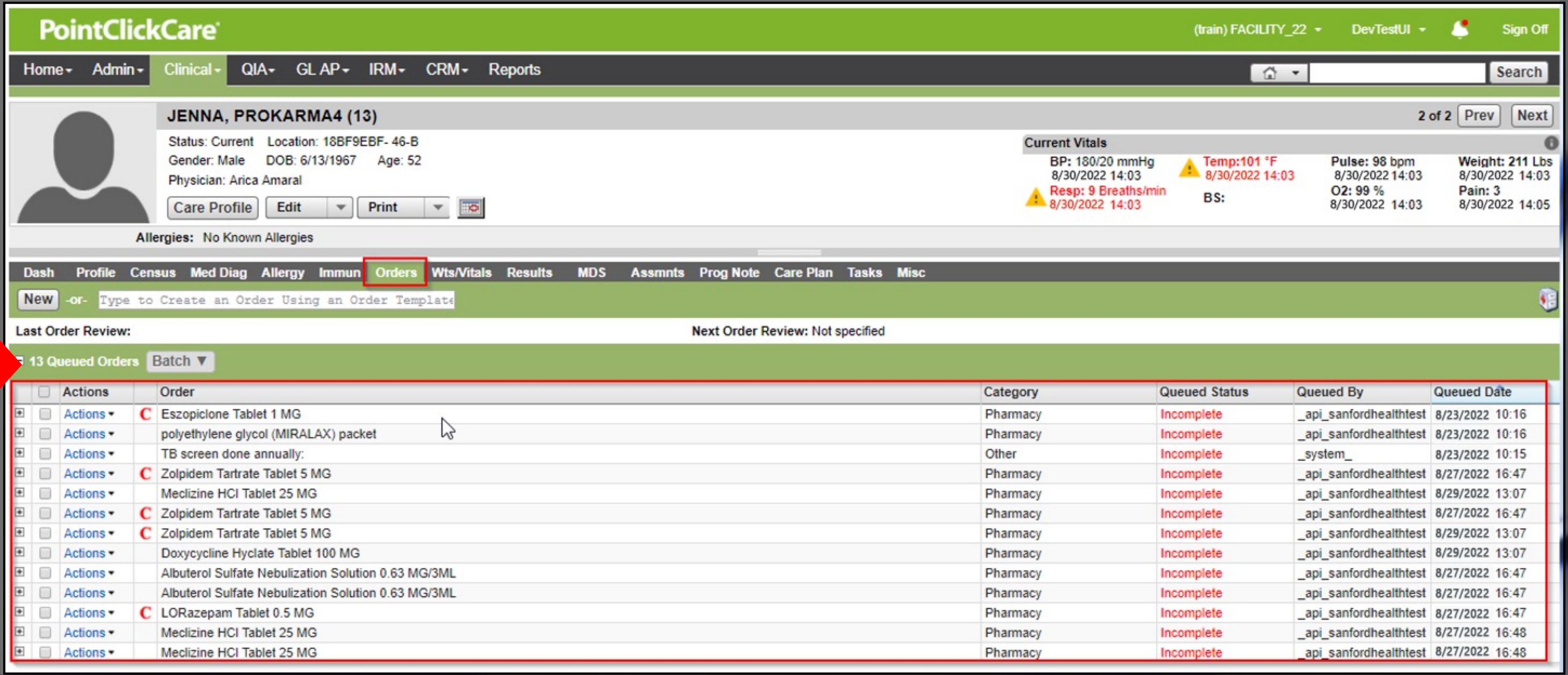

The medications gathered from Epic are then pushed into PCC (when the patient is found after the initial search) . They will show up under the orders tab as "queued orders"

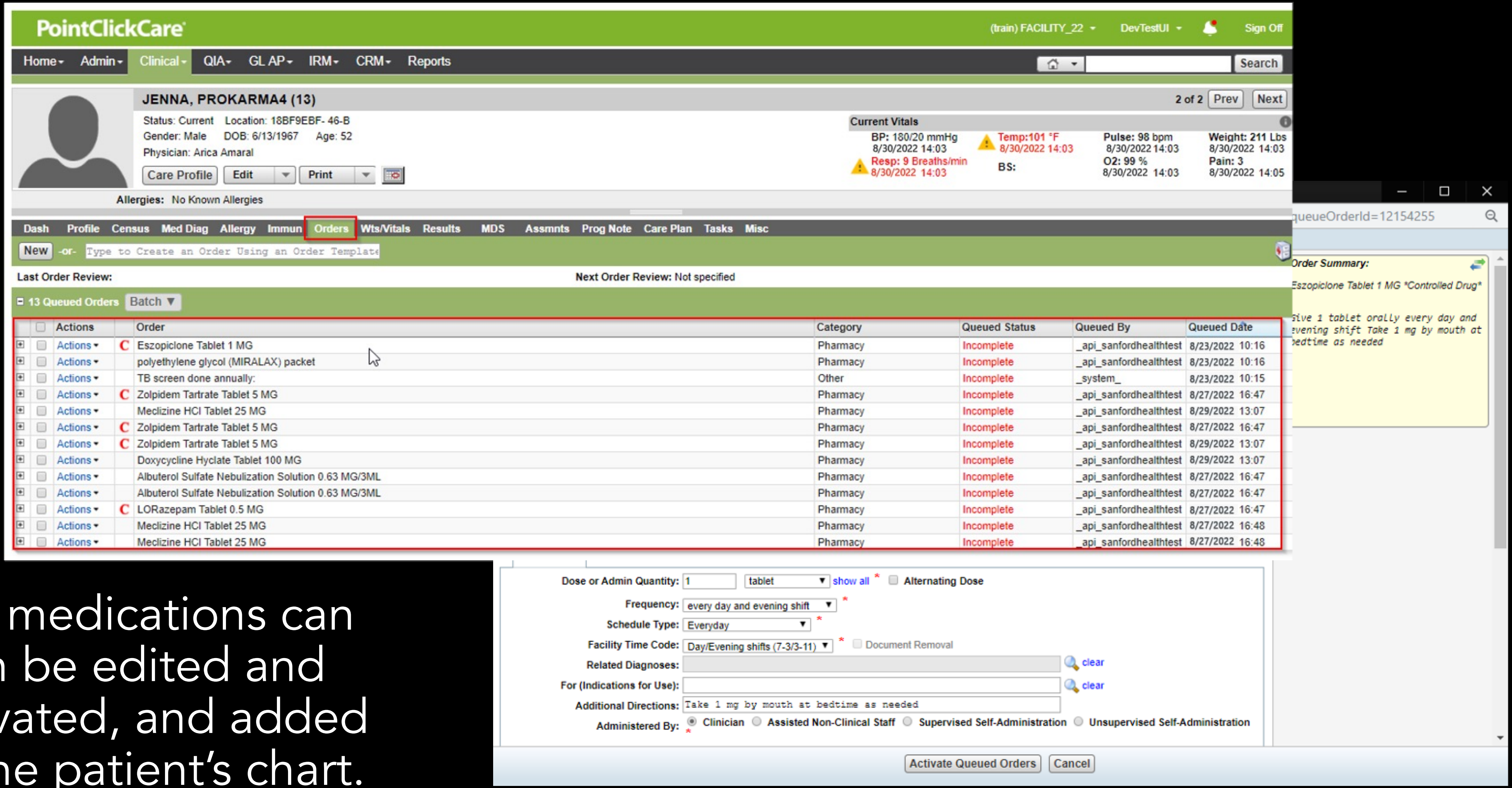

The medications can then be edited and activated, and added to the patient's chart.

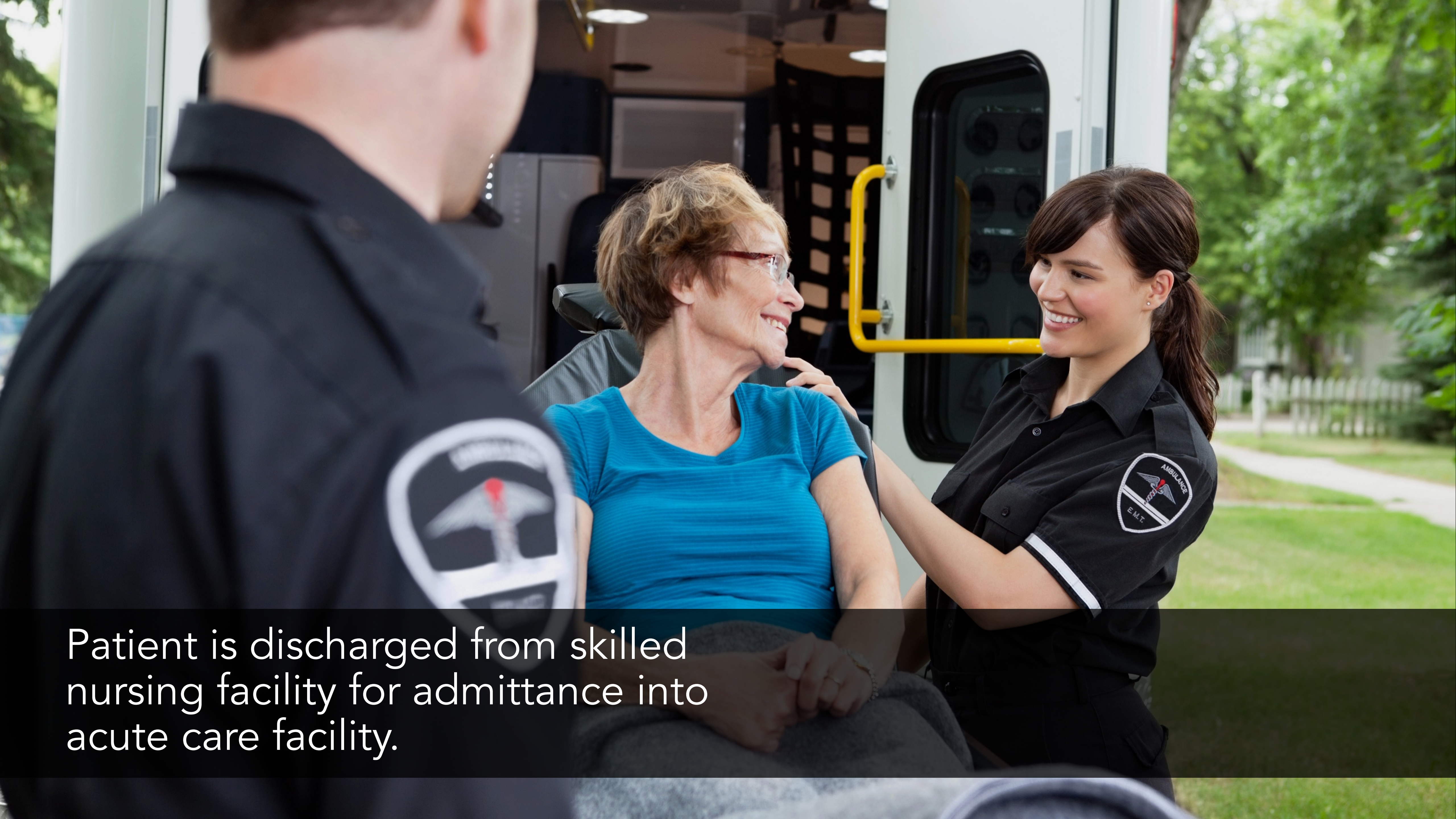
Patient is discharged from skilled nursing facility for admittance into acute care facility.
AMBULANCE
E.M.T.

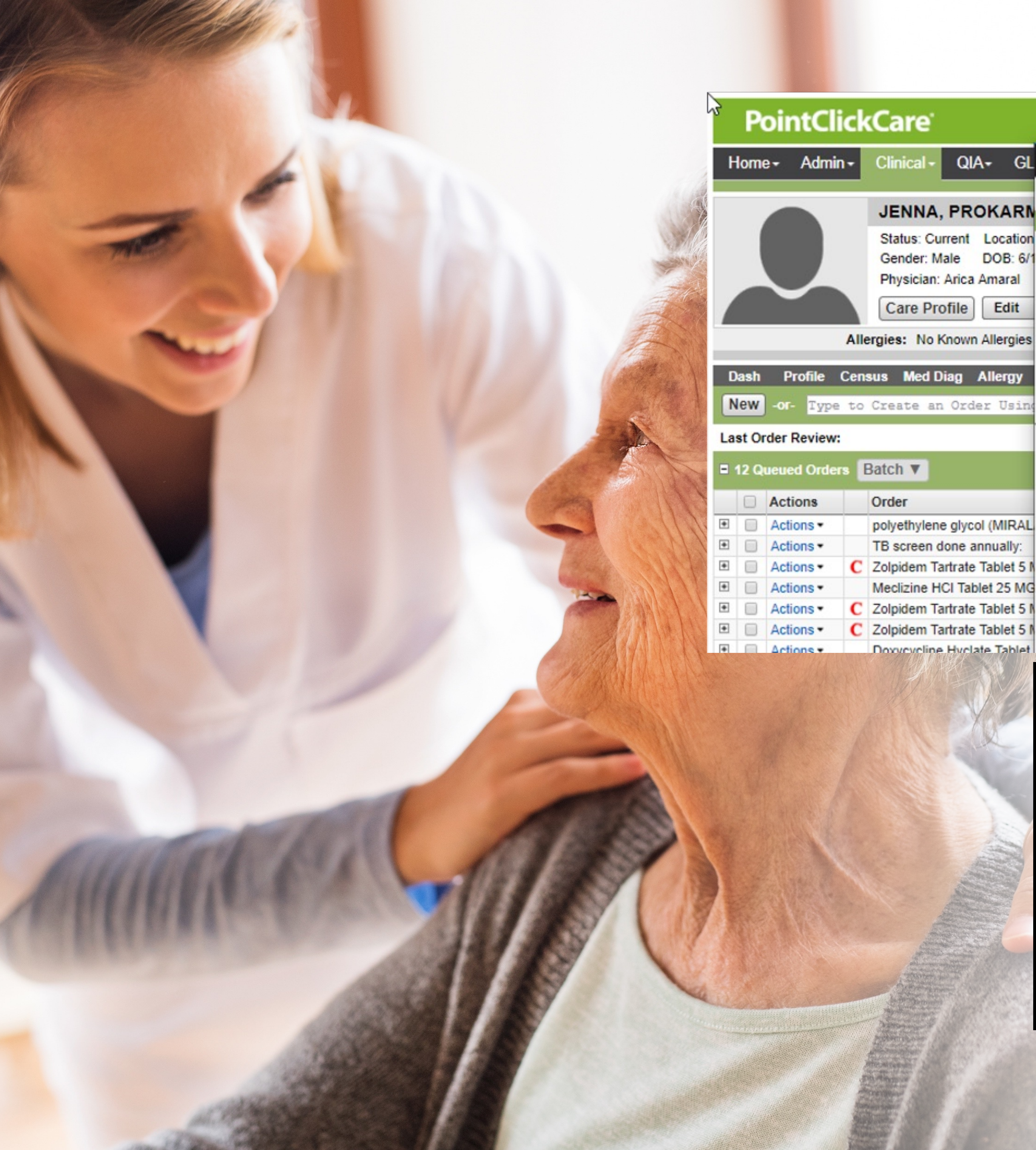

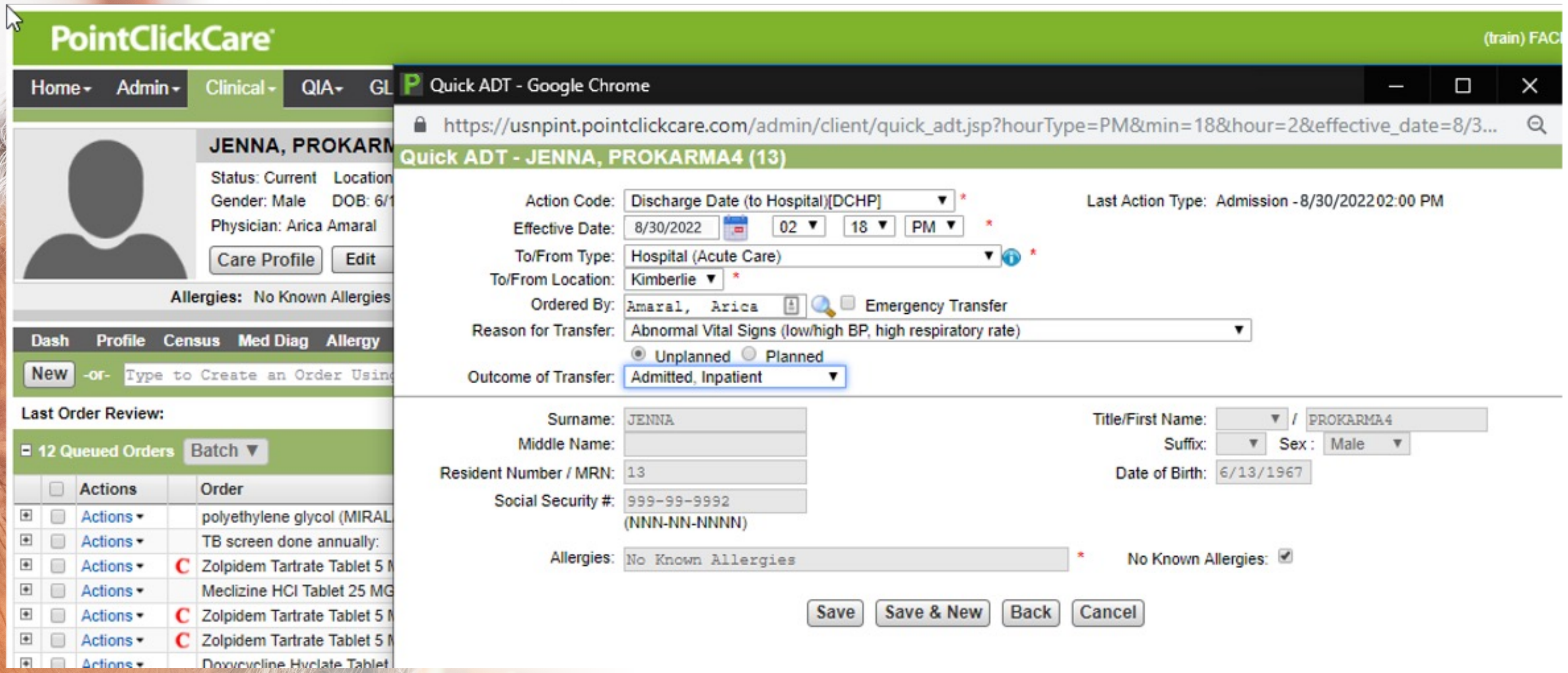

In the other direction, if a patient is discharged from a SNF to the acute care hospital a webhook message will notify Mirth Connect of the discharge.

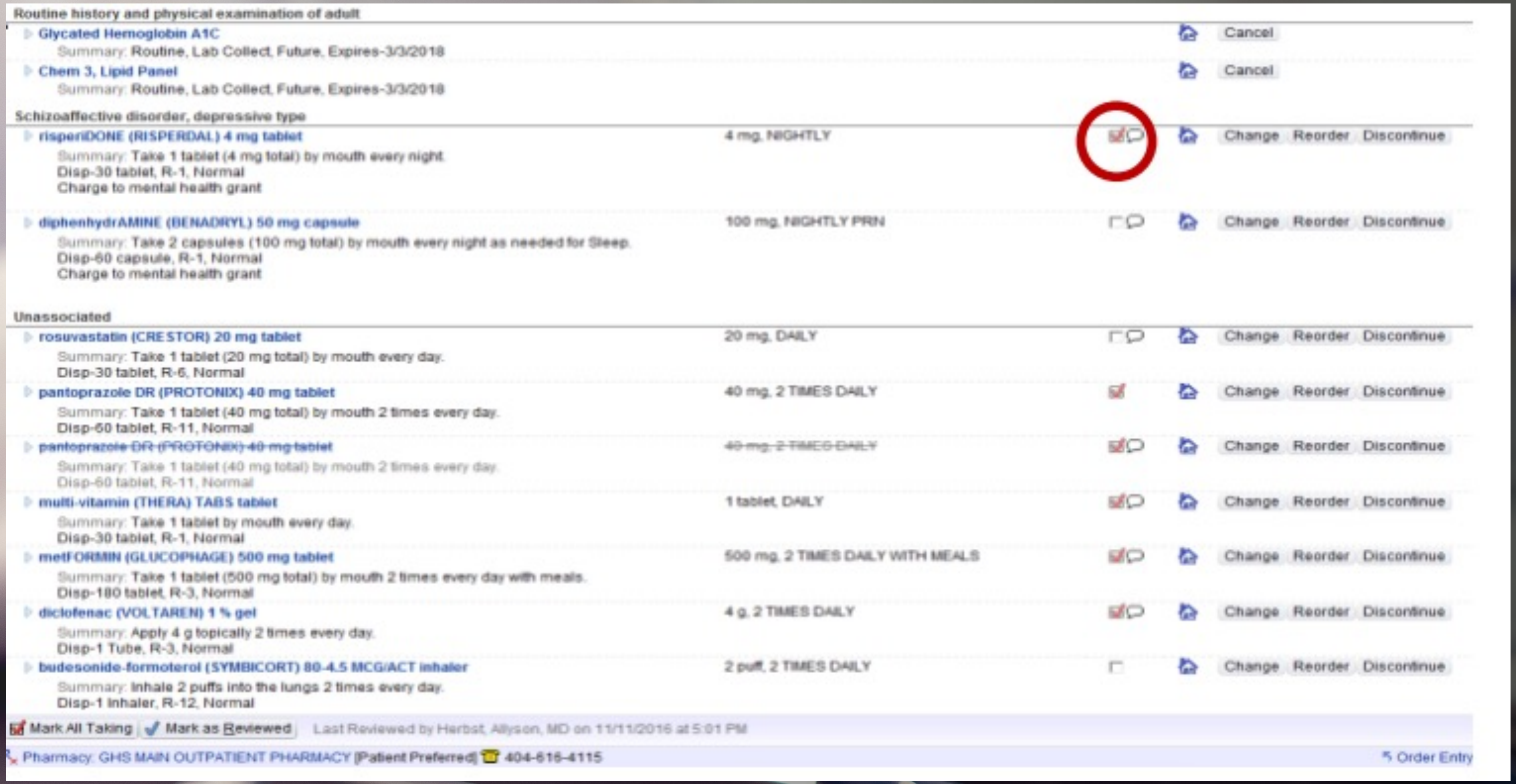

Mirth Connect will then push the CCD document to Epic. The medications will then be viewable through the Care Everywhere application and can be reconciled to the patient's chart

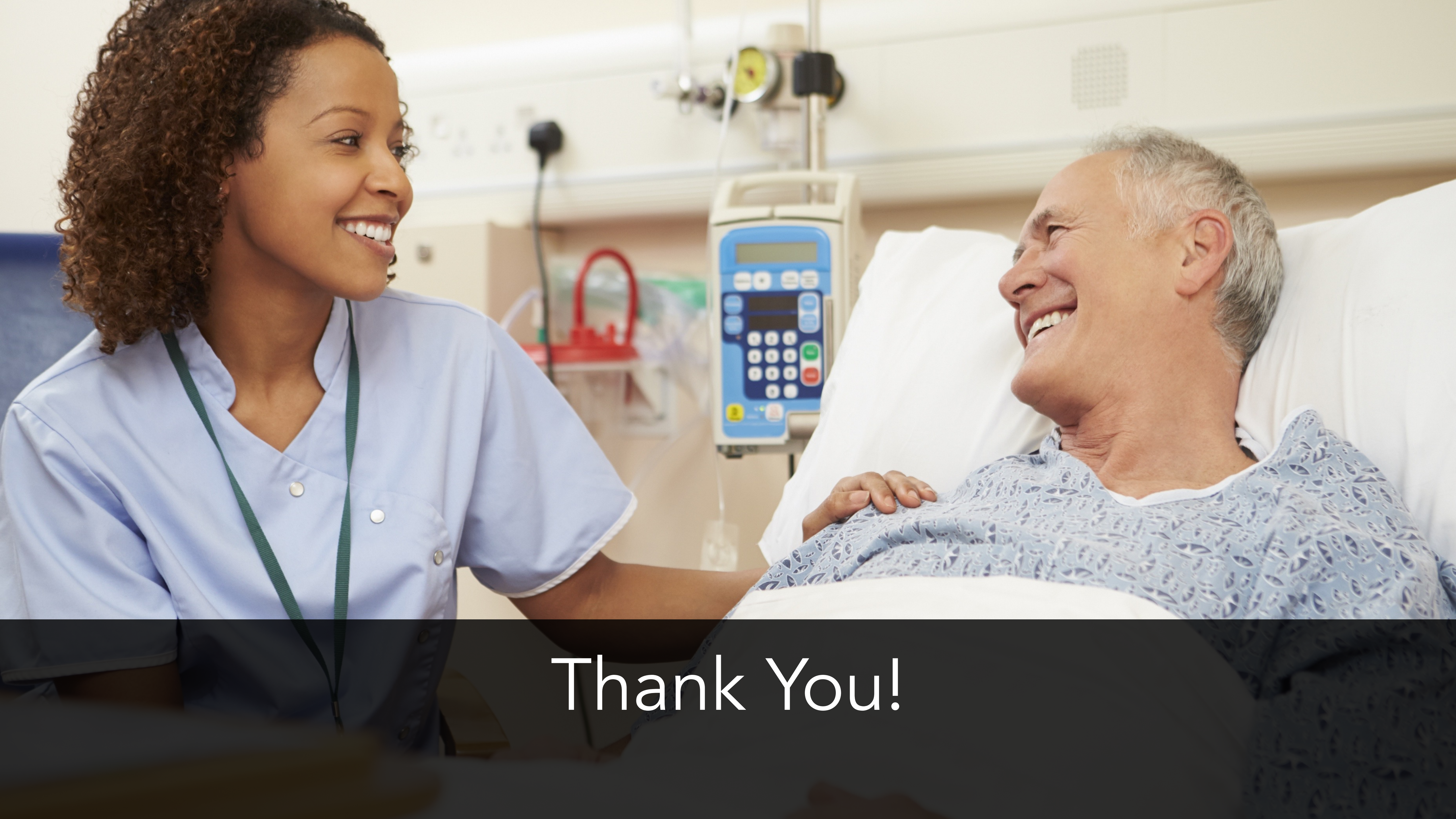
Thank You!
